# Lipidomics Reveals a Tissue-Specific Fingerprint

**DOI:** 10.3389/fphys.2018.01165

**Published:** 2018-08-28

**Authors:** Irene Pradas, Kevin Huynh, Rosanna Cabré, Victòria Ayala, Peter J. Meikle, Mariona Jové, Reinald Pamplona

**Affiliations:** ^1^Department of Experimental Medicine, Institute for Research in Biomedicine of Lleida, University of Lleida, Lleida, Spain; ^2^Baker Heart and Diabetes Institute, Melbourne, VIC, Australia

**Keywords:** adult rat tissues, lipidomics, glycerophospholipids, plasmalogens, glycerolipids, sphingolipids, cholesterol, lipid distribution

## Abstract

In biological systems lipids generate membranes and have a key role in cell signaling and energy storage. Therefore, there is a wide diversity of molecular lipid expressed at the compositional level in cell membranes and organelles, as well as in tissues, whose lipid distribution remains unclear. Here, we report a mass spectrometry study of lipid abundance across 7 rat tissues, detecting and quantifying 652 lipid molecular species from the glycerolipid, glycerophospholipid, fatty acyl, sphingolipid, sterol lipid and prenol lipid categories. Our results demonstrate that every tissue analyzed presents a specific lipid distribution and concentration. Thus, glycerophospholipids are the most abundant tissue lipid, they share a similar tissue distribution but differ in particular lipid species between tissues. Sphingolipids are more concentrated in the renal cortex and sterol lipids can be found mainly in both liver and kidney. Both types of white adipose tissue, visceral and subcutaneous, are rich in glycerolipids but differing the amount. Acylcarnitines are mainly in the skeletal muscle, gluteus and soleus, while heart presents higher levels of ubiquinone than other tissues. The present study demonstrates the existence of a rat tissue-specific fingerprint.

## Introduction

The origin and early evolution of life is closely linked to the emergence of a specific class of biomolecules called lipids (Segré et al., 2001; Paleos, 2015), and their inherent self-organization ability to form membranes (Tanford, 1978). In fact, all cells/organisms from the three domains of life (archaea, bacteria, and eukaryotes) have lipid membranes (Lombard et al., 2012). The unique trait of lipids to generate membranes was later during evolution extended to new functional properties such as cell signaling and energy storage (Hulbert et al., 2014). The evolution of early organisms toward complexity was also associated with an enlargement in the structural and functional diversity of lipid species. The result is the generation of thousands of different lipids, which require cells to invest approximately 5% of their genes for synthesis (Sud et al., 2007).

In accordance with the current classification systems, lipids are grouped into eight categories: fatty acyls (FA), glycerolipids (GL), glycerophospholipids (GP), sphingolipids (SP), saccharolipids (SL) and polyketides (PK), and sterol (ST) and prenol lipids (PR). Although there are no consistent assessments of the number of discrete lipid compounds in nature, likely due to the technical challenges of elucidating chemical structures, it is estimated that the cellular lipid profile comprises more than 1000 different molecular species (Van Meer, 2005). This diversity is also extended to cellular membrane from a compositional point of view. Thus, the lipid profile varies within the lateral plane of the membrane, between the two leaflets of the lipid bilayer, between territories of the membrane, between organelles, between tissues, and between animal species (Vereb et al., 2003; Mitchell et al., 2007; Van Meer et al., 2008; Fernández et al., 2011; Gode and Volmer, 2013; Klose et al., 2013; Naudí et al., 2013, 2017; Jain et al., 2014; Barceló-Coblijn and Fernández, 2015; Cortie et al., 2015; Zhang et al., 2015; Bozek et al., 2017; Choi et al., 2018; Khrameeva et al., 2018; Xu et al., 2018). To this diversity in spatial distribution must be added the temporal factor. Thus, the lipid profile varies in time according with a circadian rhythmicity (Aviram et al., 2016), as well as during the life cycle of an organism (Naudí et al., 2015; Jové et al., 2017).

This relevance of lipids in cell structure and physiology can also be stretched to the pathological condition. Thus, a large number of human pathologies are linked to alterations of lipid homeostasis including in addition to a number of genetic disorders (Hobbs et al., 1992), common diseases such as obesity and diabetes (Huynh et al., 2017), cardiovascular disease (Kolovou et al., 2015), cancer (Yang and Han, 2016), and neurodegenerative disease (Naudí et al., 2015; Huynh et al., 2017), among others.

From all these observations it can be inferred that the complete lipid profile (or lipidome) is a dynamic and flexible system, which requires functional plasticity, also requires a specific recognition according with the location, internal controls to detect changes and confer stability, and adaptive responses in order to maintain key membrane biological properties and cellular functions within physiological homeostatic limits (Hagen et al., 2010; Naudí et al., 2013). The result is the formation and maintenance of a specific membrane lipidome.

Recent developments in mass spectrometry (MS)-based lipidomics offer the means to accurately and unambiguously to detect and quantify a large number of lipid molecular species, but there is a lack of studies reporting on lipid abundance across multiple mammalian tissues, and it remains uncertain to what extent lipids exhibit tissue specificity. In this context, we have designed a study that represents the most detailed lipidomic analysis across seven different tissues [kidney, skeletal muscle (soleus and gluteus), heart, white adipose tissue (subcutaneous and visceral) and liver] from adult rats. We are aware that we omitted other interesting tissues but we chose these tissues because their important role in organism metabolism and physiological functions. We detected and quantified a panel of lipids including 652 molecular species belonging to fatty acyl carnitines (14); diacylglycerides (DAG, 20); triacylglycerides (TAG, 44); alkyl-TAGs (TAG(O), 3); lysophosphatidylcholine (LPC, 61); alkyl-lysophosphatidylcholine (LPC(O), 10); alkenyl-LPC (LPC(P), 6); lysophosphatidylethanolamine (LPE, 14); alkenyl-LPE (LPE(P), 4); lysophosphatidylinositol (LPI, 8); lysophosphatidylserine (LPS, 4); phosphatidylcholine (PC, 68); alkyl-phosphatidylcholine (PC(O), 22); alkenyl-phosphatidylcholine (PC(P), 26); phosphatidylethanolamine (PE, 37); alkyl-PE (PE(O), 14); alkenyl-PE (PE(P), 55); phosphatidylglycerol (PG,4); phosphatidylinositol (PI, 27); phosphatidylserine (PS, 7); sphingosine (Sph, 3); sphingosine phosphate (Sph1P, 5); ceramide (Cer, 54); ceramide phosphate (Cer1P, 1); dihydroceramide (dhCer, 6); sphingomyelin (SM, 44); monohexosylceramide (MHC, 14); dihexosylceramide (DHC, 10); trihexosylceramide (THC, 6); ganglioside (GM, 8); sulfatide (Sulfatides, 6); cholesterol (COH, 1); desmosterol (D, 6); hydroxycholesterol (OHC, 10); cholesteryl ester (CE, 27); oxidized cholesteryl ester (oxCE, 2); and ubiquinone (Ub, 1). Despite the wide range of lipid species analyzed we are aware that several species were not included in the present analysis. The tissue lipid profile was determined using a LC-QQQ-MS/MS platform to systematically define specific phenotypic patterns. Our results showed that lipidomics reveals a tissue-specific fingerprint.

## Materials and methods

### Tissue isolation and homogenization

Male Wistar rats of 468.9 ± 37.8 grams of body weight and 8 months old were caged individually and maintained in a 12:12 (light:dark) cycle at 22 ± 2°C and 50 ± 10% relative humidity. Animals were fed *ad libitum* with a semi purified diet prepared by MP biochemicals (Irvine, CA). The composition of the diet (in g/100 g of diet) was: L-arginine 1.12, L-lysine 1.44, L-histidine 0.33, L-leucine 1.11, L-isoleucine 0.82, L-valine 0.82, L-threonine 0.82, L-tryptophan 0.18, L-methionine 0.86, L-glutamic acid 2.70, L-phenylalanine 1.16, L-glycine 2.33, dextrine 5.0, corn starch 31.82, sucrose 31.79, cellulose 5.0, choline bitartrate 0.20, MP vitamin diet fortification mixture 1.0, mineral mix (AIN) 3.50 and corn oil 8.0. Animals were sacrificed by decapitation. Liver, heart, kidney, skeletal muscle (gluteus and soleus; gluteus with a predominance of type II fibers and soleus with a predominance of type I; Díaz-Herreral et al., 2001; Silva Cornachione et al., 2011) and adipose tissue (subcutaneous and visceral) samples were immediately processed and frozen at −80°C for later assays. All procedures followed the protocols approved by the Institutional Committee of Care and Use of Animals (Comitè Institucional de Cura i Ús d'Animals).

For untargeted lipidomic analysis, to 50 mg of tissue 20 volumes of cold homogenization buffer (180 mM of potassium chloride (KCl), 5 mM of 3-(N.morpholino)propanesulfonic acid (MOPS), 2 mM of ethylendiamine-tetraacetic acid (EDTA), 1 mM of dethyltriamine-penta acetic acid (DTPAC) and 1 μM of 2,6-Di-tert-butyl-4-methylphenol (BHT) adjusted to pH 7,4) were added. For the targeted lipidomic analysis, to 50 mg of tissue 10 volumes of cold phosphate buffered saline solution (pH 7.47) were added. All the samples were homogenized at 4°C with and Ultra-Turrax (3420000 IKA, Germany).

### Untargeted lipidomics

Untargeted lipidomic analysis was performed using a UPLC 1290 coupled to an ESI-QTOF MS/MS model 6520 (Agilent Technologies, Barcelona, Spain) as previously described (Jové et al., 2013). For the lipid extraction, 10 μL of the homogenized tissue were mixed with 5 μL of miliQ water and 20 μL of ice-cold methanol. Samples were vigorously shaken by vortexing for 2 min and then, 250 μL of methyl tert-butyl ether (MTBE), containing internal lipid standards, were added. Samples were immersed in a water bath (ATU Ultrasonidos, Valencia, Spain) with an ultrasound frequency and power of 40 kHz and 100 W, respectively, at 10°C for 30 min. Then, 25 μL of miliQ water were added to the mixture, and organic phase was separated by centrifugation (1,400 g) at 10°C for 10 min (Pizarro et al., 2013). Lipid extracts, contained in the upper phase, were collected and subjected to mass spectrometry. A pool of all lipid extracts was prepared and used as quality controls. Internal lipid standards used were isotopically labeled lipids (Table S1). Stock solutions were prepared by dissolving lipid standards in MTBE at a concentration of 1 mg/mL, and working solutions were diluted to 2.5 μg/mL in MTBE. Lipid extracts were analyzed following a previously published method (Castro-Perez et al., 2010). Sample compartment of the UHPLC was refrigerated at 4°C and for each sample, 10 μl of lipid extract was applied onto 1,8 μm particle 100 × 2,1 mm id Waters Acquity HSS T3 column (Waters, Mildord, MA) heated at 55°C. The flow rate was 400 μL/min with solvent A composed of 10 mM ammonium acetate in acetonitrile-water (40:60, v/v) and solvent B composed of 10 mM ammonium acetate in acetonitrile-isopropanol (10:90, v/v). The gradient started at 40% of mobile phase B and reached 100% B in 10 min and held for 2 min. Finally, the system was switched back to 60% of mobile phase B and was equilibrated for 3 min. Duplicate runs of the samples were performed to collect positive and negative electrospray ionized lipid species in a TOF mode, operated in full-scan mode at 100 to 3000 m/z in an extended dynamic range (2 GHz), using N2 as nebulizer gas (5 L/min, 350°C). The capillary voltage was set at 3,500 V with a scan rate of 1 scan/s. Continuous infusion using a double spray with masses 121.050873, 922.009798 (positive ion mode) and 119.036320, 966.000725 (negative ion mode) was used for in-run calibration of the mass spectrometer.

### Targeted lipidomics

Targeted lipidomic analysis was performed using a LC ESI-QQQ MS/MS model 6490 (Agilent Technologies, Melbourne, Australia). 652 lipid species were detected, 14 were acylcarnitines of the fatty acyl (FA) category; 46 were cholesterol derivatives of the category of sterol lipids (ST) and 157 were sphingolipids (SP) including ceramides, gangliosides, sphingomyelin and sulfatides. Of the glycerolipids (GL) category 67 lipids were detected, 20 of them diacylglycerols and the rest of them triacylglycerols, mostly unsaturated. Most of the lipids were part of the glycerophospholipid (GP) category; concretely 367 lipid species were detected; 124 with ethanolamine (being 73 ether lipids, 14 of them with an alkyl ether bond and 59 plasmalogens); 193 with choline (being 62 ether lipids, 32 of them plasmalogens as well and 32 plasmanyl species); 11 with serine; 35 with inositol and only 4 lipid species conjugated with another molecule of glycerol. The only lipid detected in the prenol (PR) category was ubiquinone. Sample tissues were randomized prior to lipid extraction. Lipids were extracted in a single phase chloroform:methanol (2:1) procedure as previously described (Meikle et al., 2011). Briefly, to 10 μL of the stock tissue homogenates, 200 μL of chloroform/methanol (2:1, v/v) were added together with 10 μL of internal standards in chloroform/methanol (1:1, v/v). Lipid standards stock solutions were prepared by dissolving lipid standards (Table S1) in chloroform:methanol (1:1,v/v) at 100 pmol except for cholesterol that was 10,000 pmol, cholesteryl ester 1,000 pmol, and sphingomyelin and diacylglycerol that were at 200 pmol. Finally, for the dihydroceramides and hexosylceramides the working solutions were at 50 pmol. The mixture was mixed for 10 min on a rotary mixer, sonicated in a water bath (18–24°C) for 30 min, left to stand on the bench for 20 min and then centrifuged at 16,000 × g at 20°C for 10 min. The supernatant was transferred to a 96-well plate and dried under a stream of nitrogen gas at 40°C. Samples were reconstituted with 50 μL H_2_O-saturated 1-butanol and sonicated for 10 min. Then, 50 μL of 10 mM ammonium formate in methanol was added. The extract was centrifuged at 1,700 × g at 20°C for 5 min. The recovery efficiencies of the lipid extraction method for each lipid subclass were previously published in other work (Alshehry et al., 2015). Finally, supernatant was transferred into a 0.2 mL glass insert with Teflon insert cap for analysis by LC ESI-MS/MS. Technical quality controls (internal lipid standard mix solution) were injected every 10 samples as well as lipid extraction quality controls (plasma samples with internal lipid standard mix solution) every 15 samples. One microliter of lipid extract was applied onto ZORBAX eclipse plus C18 column, 2.1 × 100 mm 1.8 μm, (Agilent Technologies) heated to 60°C and the auto-sampler regulated to 25°C. Flow rate was 400 μL/min with solvent A composed of 10 mM ammonium formate in acetonitrile-water-isopropanol (50:30:20, v/v) and solvent B composed of 10 mM ammonium formate in acetonitrile-water-isopropanol (9:1:90, v/v). The gradient started at 10% of mobile phase B, eached 100% B in 11 min and held for 1 min. Finally, the system was switched back to 10% of mobile phase B and was equilibrated for 3 min. Data was collected in the multiple reaction monitoring scan type and the capillary voltage was set at 3,500 V. Positive polarity of electrospray ionization was set using N2 at 20 psi as nebulizer gas (17 L/min, 150°C) and the sheath gas parameters were flow at 10 L/min and temperature at 200°C. For all the standard lipid species the cell accelerator voltage was 5 volts, except for the Sph(d17:1) that was 4 volts and fragmentor was 380 volts. The conditions for tandem mass spectrometry quantification of the 652 lipid species detected by the targeted lipidomic analysis are reported in Table S2. To determine the acyl composition for each glycerophospholipid species, we performed MS2 experiments in pooled plasma samples on our chromatography set-up (Supplementary Figure 1). Phosphatidylethanolamine and phosphatidylinositol species were fragmented in negative ionization mode and product ions corresponding to their acyl chains were examined. Phosphatidylcholine and sphingomyelin species were examined in positive ion mode in the presence of lithium acetate, and the product ions of the [M+Li]+ precursor was used to determine their acyl composition (Hsu and Turk, 2003). Moreover, to determine whether observed peaks were either ether lipids or plasmalogens, we utilized our quality control plasma extracts. Plasma extracts were dried down under nitrogen and exposed to HCl vapor for 5 min. Sample was then reconstituted in the same solvent (butanol:methanol, 1:1). Injection of quality control samples with and without exposure to HCl was used to determine peaks corresponding to plasmalogens, where the susceptibility of the vinyl-ether bond to acid results in complete hydrolysis (Supplementary Figure 2). Technical quality control coefficient variation (CV) was 7.6% across measured lipids suggesting a good technical reproducibility.

### Data analysis

For both untargeted and targeted analysis, the MassHunter Data Analysis Software (Agilent Technologies) was used to collect the results. In the untargeted approach the MassHunter Qualitative Analysis Software (Agilent Technologies, Barcelona, Spain) to obtain the molecular features of the samples, representing different, co-migrating ionic species of a given molecular entity using the Molecular Feature Extractor (MFE) algorithm (Agilent Technologies, Barcelona, Spain) (Jové et al., 2013). MassHunter Mass Profiler Professional Software (Agilent Technologies, Barcelona, Spain) was used to perform a non-targeted lipidomic analysis over the extracted features. Only those features with a minimum abundance of 5000 counts and 2 ions as a minimum were selected. After that, the molecular characteristics in the samples were aligned using a retention time window of 0,1% ± 0,25 min and 20,0 ppm ± 2,0 mDa. To avoid background, only common features (found in at least 50% of the samples of the same condition) were taken into account to correct for individual bias. The features defined by exact mass and retention time were searched against the LIPID MAPS database (accuracy < 20 ppm) (Fahy et al., 2007). The identities obtained were compared to retention time of the authentic standards added. Finally, identities were confirmed by MS/MS by checking the MS/MS spectrums using LipidBlast software (Kind et al., 2013) and LipidMatch, a R-based tool for lipid identification (Koelmel et al., 2016).

In the targeted analysis, the software MassHunter Quantitative Analysis (Agilent Technologies) was used to quantify each lipid species. Concentrations were obtained first in pmol/ml and then normalize to micrograms of tissue. Multivariate statistics (hierarchical clustering and principal component analysis), Variable importance in projection score and one-way anova were done using Metaboanalyst software (Xia and Wishart, 2016).

## Results

### Clustering mammalian tissues based on their lipidome

**Figure 1:**
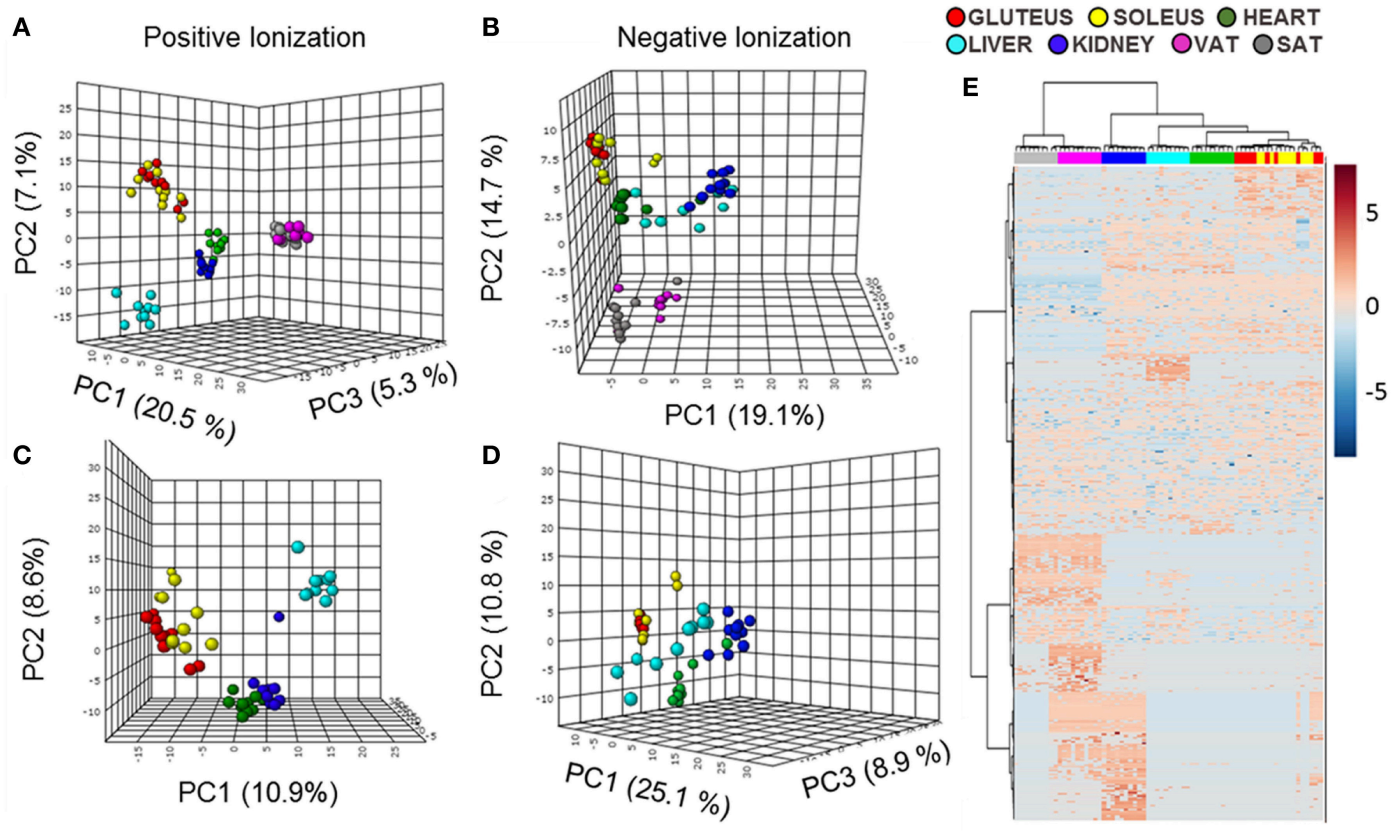
Multivariate statistics reveal a specific lipidomic profile for each tissue. **(A,B)** Principal component analysis (PCA) representation of the lipidome of all the tissues in positive and negative ionization polarities. **(C,D)** PCA representation of the lipidomic profiles of all the tissues except for both type of adipose tissues in positive and negative ionization polarities. X: Principal component 1 (PC1), Y: Principal component 2 (PC2), Z: Principal component 3 (PC3). **(E)** Hierarchical clustering algorithm represented by a heat map of the lipid species detected in both polarities of ionization in all the tissues.

In a first approach to study the distribution of lipids across tissues an untargeted analysis in a LC-ESI-QTOF mass spectrometer was performed. The samples selected for this study were heart, liver, kidney (represented by renal cortex), gluteus (as a representation of glycolytic fibers of skeletal muscle), soleus (as a representation of oxidative fibers of skeletal muscle), visceral adipose tissue (VAT) and subcutaneous adipose tissue (SAT) from adult male Wistar rats. After data filtering, the number of lipid species detected was 1,264 (970 in positive and 294 in negative ionization mode) where multivariate statistics were applied. In the principal component analysis (PCA) representations of every type of sample analyzed (Figures 1A,B) most of the tissues were well separated based on their lipidome and those tissues with similar functions or common developmental origin were clustered together. On this wise, first division between samples separated both types of adipose tissue from the rest of the tissues. Furthermore, as the lipidomic profile of adipose tissue was completely different of the other samples, a second PCA approach without white adipose tissue was performed (Figures 1C,D). The lipidome detected from positive and negative polarities clustered separately for all the tissues. Gluteus and soleus were together and in hierarchical clustering analysis represented by a heat map they appeared evenly mixed (Figure 1E), being that their lipid profile/composition was very similar. The tissue most closely clustering with skeletal muscle was, as expected, cardiac muscle. The PCA results from lipids detected with positive polarity showed that kidney and heart were closely clustered relative to hepatic tissue. This situation change for those lipids detected with negative polarity or in the global analysis with the hierarchical clustering algorithm, where kidney and liver were closely aligned.

### Assessing lipid across mammalian tissues

After assessing the lipidome characteristics in each tissue using an untargeted analysis, a more specific approach was performed using a targeted analysis. As described in the Materials and Methods section we were able to quantify 652 lipid species. Concentration of each lipid species was represented in nmol/g of tissue and the values of the concentration of all the species detected are pictured in Dataset 1 (Supplementary Information), while the concentrations of each subclass analyzed are represented in Table 1.

**Table 1:** Lipid concentration in nmol/g of tissue organized according LIPIDMAPS classification and analyzed by targeted lipidomics.

**Lipid Class**	**N° of species**	**Liver**	**Kidney**	**Heart**	**Gluteus**	**Soleus**	**VAT**	**SAT**
**FATTY ACYLS**								
FAC	14	2.30 ± 0.18^d***^; c^*^	13.21 ± 1.04^d***^; c^*^	4.66 ± 0.64^d***^; c^*^	151.78 ± 45.99^b, e, f, g***^; a^**^	119.34 ± 29.46^a, b, e, f, g^*^^	2.88 ± 0.37^d***^; c^*^	6.61 ± 1.03^d**^, c^*^
Subtotal FA	14	2.30 ± 0.18^d***^; c^*^	13.2 ± 1.04^d***^; c^*^	4.66 ± 0.64^d***^; c^*^	152 ± 46^b, e, f, g***^; a^**^	119 ± 29^a, b, e, f, g^*^^	2.88 ± 0.37^d***^; c^*^	6.61 ± 1.03^d**, c^*^^
**GLYCEROLIPIDS**								
DAG	20	14027.71 ± 858.67^a***^	4910.17 ± 324.22^a***^; b^*^	3781.92 ± 422.55^a***^; b^*^	1456.87 ± 70.87^a***^; b^**^	2519.70 ± 325.54^a***;b^*^^	18415.56 ± 3120.27^a***^; d^**^;c, e, f^*^	55008.16 ± 6999.58^τ^^***^
TAG	44	4585.44 ± 240^a, b, d, e, f***^; c^**^	4910.17 ± 76.92^a, b, g***^	1011.49 ± 124.02^a, b, g***^	1391.58 ± 1456.87^a, b, g***^	2435.74 ± 2519.70^a, b***^; g^**^	15656.76 ± 466.5^τ^^***^	38037.93 ± 519.71^τ^^***^
TAG(O-)	3	2.42 ± 0.19^a, b***^	1.48 ± 0.22^a, b***^	3.08 ± 0.39^a, b***^	1.19 ± 0.09^a, b***^	2.65 ± 0.46^a***^; b^**^	46.83 ± 8.32^a, d, e, f, g***^; c^**^	133.93 ± 14.87^τ^^***^
Subtotal GL	67	18, 616 ± 1, 038^a***^; b^**^;d, e^*^	5, 892 ± 358^a, b***^	4, 796 ± 512^a, b***;g^*^^	2, 850 ± 241^a, b***^; g^*^	4, 958 ± 494^a, b***^	34, 119 ± 3, 394^a, c, d, e, f***^; g^**^	93, 180 ± 6, 763^τ^^***^
**STEROL LIPIDS**								
COH	1	5912.37 ± 297.33^a, b, c, d***^; e^**^;f^*^	8819.47 ± 542.59^a, b, c, d, e***^; g^*^	3481.39 ± 349.42^f***^; g^**^;b^*^	1171.76 ± 69.38^f, g***^	1818.28 ± 230.43^f, g***^	808.01 ± 169.07^f, g***^; e^*^	2540.08 ± 325.85^f, g***^
Desmosterol	6	18.84 ± 0.88^a, b, c, d, e***^; f^**^	13.45 ± 0.71^g**^	12.01 ± 0.40^g***^	9.36 ± 0.11^g***^	9.21 ± 0.08^g***^	12.35 ± 1.62^g***^	11.27 ± 1.01^g***^
OHC	10	15.95 ± 1.14^τ^^***^	10.33 ± 0.38^a, b, g***;c**^	9.70 ± 0.42^g***;a, b**^	8.22 ± 0.78^g***;a, b^*^^	6.10 ± 0.33^g***;f**^	4.50 ± 0.72^f, g***;e**;d^*^^	4.17 ± 0.27^f, g***;e**;d^*^^
CE	27	3925.98 ± 236.69^τ^^***^	818.25 ± 167.96^g***;b, d^*^^	498.76 ± 49.63^g***^	123.59 ± 11.11^g***^	125.52 ± 12.68^g***^	69.39 ± 6.85^g***^	117.9 ± 14.28^g***^
oxCE	2	71.34 ± 4.97^τ^^***^	33.52 ± 2.34^g***^	24.13 ± 0.82^g***^	31.09 ± 0.65^g***^	29.40 ± 0.93^g***^	42.15 ± 6.86^g***^	39.62 ± 3.56^g***^
Subtotal ST	46	317 ± 413^a, b, c, d, e, ***^	447 ± 620^a, b, c, d, e, ***^	178 ± 251^b, f, g***;d**;c^*^^	61.4 ± 82.1^f, g***;e**;^	93.5 ± 134^f, g***;e^*^^	42.4 ± 66.1^e, f, g***^	130 ± 188^f, g***^
**PRENOL LIPIDS**								
Ubiquinone	1	238.41 ± 25.87^e, f***^	621.84 ± 36.66^τ^^***^	1165.46 ± 67.35^τ^^***^	55.49 ± 5.78^e, f***^	103.36 ± 15.10^e, f***^	5.82 ± 0.37^e, f***^	6.13 ± 0.40^e, f***^
Subtotal Pr	1	238.41 ± 25.87^e, f***^	621.84 ± 36.66^τ^^***^	1165.46 ± 67.35^τ^^***^	55.49 ± 5.78^e, f***^	103.36 ± 15.10^e, f***^	5.82 ± 0.37^e, f***^	6.13 ± 0.40^e, f***^
**GLYCEROPHOSPHOLIPIDS**								
LPC	61	469.32 ± 75.39^a, b, c, d***;e, f**^	181.22 ± 17.31^g**;b, d^*^^	248.78 ± 41.13^g**;b^*^^	27.19 ± 1.50^g**;f^*^^	62.66 ± 7.68^g***^	15.83 ± 2.25^g***;e, f^*^^	60.62 ± 6.15^g***^
LPC(O-)	10	0.86 ± 0.11^f***;b**^	2.25 ± 0.14^τ^^***^	0.49 ± 0.07^f***^	0.28 ± 0.03^f***^	0.49 ± 0.05^f***^	0.17 ± 0.02^f***^	0.65 ± 0.08^f***^
LPC(P-)	6	0.07 ± 0.007^c, d, e, f***^	0.99 ± 0.06^a, b, d, g***;c^*^^	0.83 ± 0.09^a, b, g***^	0.60 ± 0.05^b, f, g***;a**^	0.71 ± 0.09^a, b, f, g***^	0.05 ± 0.01^c, d, e, f***^	0.18 ± 0.03^c, e, f***d**^
LPE	14	513.26 ± 46.99^τ^^***^	140.99 ± 10.00^g***;b, d**;a^*^^	142.41 ± 15.62^g***;b^*^^	21.27 ± 1.28^g***;f**^	46.98 ± 6.09^g***^	12.13 ± 1.82^g***;f**;e^*^^	25.37 ± 2.34^g***;f^*^^
LPE(P-)	4	0.57 ± 0.07^e, f***;c**^	11.99 ± 0.88^τ^^***^	7.98 ± 0.88^a, b, d, f, g***;c^*^^	1.94 ± 0.16^e, f, g***^	4.05 ± 0.45^f***;b, g**;e^*^^	0.46 ± 0.05^e, f***;c**^	1.73 ± 0.25^e, f***^
LPI	8	17.89 ± 1.46^τ^^***^	3.74 ± 0.27^g***;b^*^^	1.90 ± 0.16^g***^	0.50 ± 0.03^g***^	0.67 ± 0.05^g***^	0.31 ± 0.04^g***;f^*^^	0.58 ± 0.06^g***^
LPS	4	0.37 ± 0.036^b, d***;c**^	0.33 ± 0.03^b, d***;c^*^^	0.30 ± 0.02^b**^	0.17 ± 0.02^f, g***^	0.21 ± 0.02^g**;f^*^^	0.12 ± 0.02^f, g***;e**;a^*^^	0.27 ± 0.02^b^*^^
PC	68	9216.98 ± 587.49^a, b, c, d***;e, f**^	5909.85 ± 260.94^a, b, d***;c, g**^	7041.58 ± 663.25^a, b, d***;g**;c^*^^	3033.14 ± 98.35^e, f, g***;b**^	3910.61 ± 524.04^b, g***;f**;a, e^*^^	297.12 ± 46.39^c, e, f, g***;d**^	948.94 ± 106.52^e, f, g***;c^*^^
PC(O-)	22	50.78 ± 3.98^a, b, f***^	93.52 ± 5.32^τ^^***^	36.10 ± 4.02^f***;b**^	33.50 ± 0.97^f***;b**^	34.98 ± 6.29^f***;b**^	3.12 ± 0.42^f***;c, d**^	15.37 ± 2.51^f***^
PC(P-)	26	25.24 ± 1.59^d, e***;f**;c^*^^	101.52 ± 7.28^b***;a, g**^	230.80 ± 25.31^a, b, g***^	159.85 ± 7.76^a, b, g***^	139.06 ± 30.22^a, b**, g^*^^	3.07 ± 0.46^d, e, f***;c**^	12.10 ± 1.94^d, e***;c, f**^
PE	37	8768.60 ± 578.37^τ^^***^	5082.89 ± 235.50^a, b, c, d, g***^	5383.97 ± 538.30^a, b, c, d, g***^	726.62 ± 36.58^e, f, g***^	1093.26 ± 186.78^e, f, g***^	78.81 ± 11.04^e, f, g***^	278.35 ± 31.27^e, f, g***^
PE(O-)	14	14.20 ± 0.87^e, f***^	72.24 ± 3.61^a, b, c, d, g***^	72.79 ± 6.93^a, b, c, d, g***^	17.00 ± 1.01^e, f, g***^	32.84 ± 6.43^b, e, f, g***;a**^	0.81 ± 0.10^c, e, f, g***^	3.48 ± 0.53^e, f, g***;c**^
PE(P-)	55	478.16 ± 29.06^e, f***^	2292.72 ± 135.62^a, b, g***;d**;c^*^^	4610.95 ± 452.93^a, b, c, d, g***^	1087.56 ± 49.52^e***;f**^	1383.14 ± 233.21^e***;f^*^^	54.96 ± 7.64^e, f***^	262.24 ± 49.14^e, f***^
PG	4	94.59 ± 6.03^e***^	80.71 ± 4.70^b, e**;a^*^^	449.30 ± 43.05^a, b, c, d, g***;f**^	52.52 ± 3.15^e***^	66.37 ± 8.47^e***^	1.37 ± 0.17^e***;f**^	3.78 ± 0.31^e***;f^*^^
PI	27	3151.23 ± 220.17^τ^^***^	1467.80 ± 68.51^a, b, d, g***;c**^	1072.04 ± 118.61^b, g***;a**^	499.78 ± 23.44^f, g***^	661.05 ± 102.45^g***;f**^	36.13 ± 4.95^e, f, g***^	111.45 ± 13.45^f, g***;e**^
PS	7	537.89 ± 34.65^a, b, d, f***;c**^	1147.16 ± 46.57^τ^^***^	396.24 ± 36.33^f, g***;b1**^	138.72 ± 5.93^f, g***^	207.00 ± 27.26^f***;g**^	37.58 ± 5.48^f, g***;e**^	134.36 ± 18.32^f, g***^
Subtotal GP	367	23, 340 ± 1, 398^a, b, c, d***;f**^	16, 590 ± 723^a, b, c, d***;g**^	19, 696 ± 1, 903^a, b, c, d***^	5, 787 ± 216^e, f, g***^	7, 626 ± 1, 122^e, f, g***;b**^	542 ± 79^e, f, g***;c**^	1, 859 ± 224^e, f, g***^
**SPHINGOLIPIDS**								
Sph	3	75.88 ± 4.14^a, b, c, d***;e^*^^	89.82 ± 4.50^a, b, c, d, e***^	61.38 ± 4.12^b, c, d, f***;a**;g^*^^	29.42 ± 0.51^e, f, g***^	31.94 ± 1.49^e, f, g***^	29.55 ± 0.93^e, f, g***^	35.46 ± 2.42^f, g***;e**^
Sph1P	5	2.34 ± 0.27	2.55 ± 0.21	2.54 ± 0.25	2.19 ± 0.13	2.26 ± 0.15	2.16 ± 0.23	2.72 ± 0.23
Cer	54	801.58 ± 40.03^τ^^***^	498.95 ± 35.17^τ***^	139.45 ± 12.28^f, g***^	66.66 ± 1.98^f, g***^	85.42 ± 10.38^f, g***^	83.02 ± 10.60^f, g***^	176.34 ± 17.53^f, g***^
Cer1P	1	0.05 ± 0.007	0.06 ± 0.005	0.05 ± 0.007	0.04 ± 0.003	0.04 ± 0.004	0.04 ± 0.005	0.05 ± 0.005
dhCer	6	12.37 ± 0.53^a, b, c, d, e, f***^	6.55 ± 0.37^a, b, c, d, g***;e**^	3.80 ± 0.21^g***;f**;b^*^^	2.58 ± 0.13^f, g***^	2.78 ± 0.14^f, g***^	1.85 ± 0.20^f, g***;e^*^^	2.83 ± 0.14^f, g***^
SM	44	1067.38 ± 65.02^f***;b**;d^*^^	3340.50 ± 186.96^a, b, c, d, e, g***^	598.39 ± 60.38^f***^	216.23 ± 6.77^f***;g^*^^	301.59 ± 34.75^f***^	88.56 ± 13.47^f***;g**^	284.94 ± 36.42^f***^
MHC	14	113.79 ± 9.33^b, d***;e**^	89.00 ± 7.87^b, c^*^^	16.30 ± 2.18^c***;g**^	7.97 ± 0.68^c, g***^	146.99 ± 29.12^b, d, e***;a**;f^*^^	5.89 ± 0.52^c, g***;f^*^^	45.63 ± 11.70^c**^
DHC	10	17.43 ± 1.65^a, b, d***^	12.64 ± 0.66^b***;a**;d*^	20.21 ± 1.89^a, b, d***^	6.87 ± 0.34^c, e, f, g***^	20.79 ± 1.77^a, b, d***^	1.69 ± 0.21^c, e, f, g***^	4.80 ± 0.73^c, e, g***;f**^
THC	6	1.07 ± 0.13	1.32 ± 0.12	1.17 ± 0.11	0.98 ± 0.13^a^*^^	0.79 ± 0.08^a**^	1.03 ± 0.06	1.59 ± 0.11^c**;d^*^^
GM	8	17.45 ± 0.89^b***;d, e**;a^*^^	17.01 ± 0.89^a, b, d***^	31.53 ± 2.43^a, b, c, d***;g**^	6.75 ± 0.55^e, f***;g**^	13.91 ± 1.40^f***;b**^	2.42 ± 0.28^e, f, g***;c**^	7.44 ± 0.98^e, f***;g^*^^
Sulfatides	6	0.33 ± 0.03^c***;f**^	3.47 ± 0.68^b, d, e, g****^	0.30 ± 0.05^c***;f**^	0.27 ± 0.03^c***;f**^	4.36 ± 0.96^a, b, d, e, g***^	0.12 ± 0.02^c***;f**^	0.72 ± 0.23^c***^
Subtotal SP	157	2, 110 ± 102^τ^^***^	4, 062 ± 216^τ^^***^	875 ± 81.4^f, g***;b**;d*^	340 ± 8.16^f, g***;e^*^^	608 ± 68.9^f, g***^	216 ± 24.4^f, g***;e**^	563 ± 59.9^f, g***^
**TOTAL LIPIDS DETECTED**								
Total	652	54, 250 ± 2, 462^a, c, d, e***;b, f^*^^	36, 874 ± 1, 833^a, d***;c, g^*^^	30, 564 ± 2, 836^a, g***;d**^	10, 528 ± 365^a, b, f, g***^	15, 404 ± 1, 681^a, g***;b, d**^	35, 823 ± 3, 658^a, d***;c, g^*^^	98, 328 ± 7, 321^τ***^

Values are expressed as mean ± SEM from 8 to 10 animals. Statistical analysis of each subclass concentrations across tissues analyzed by one-way ANOVA had a p < 0.001 in every subclass except for those species conjugated with phosphate. Sphingosine phosphate (Sph1P) p**-**value was 0.7105 and for ceramide phosphate (Cer1P) p**-**value was 0.2303. Post**-**hoc Tukey multiple test was performed for each tissue and organ. Statistical significance is represented in the table by letters: a means is significantly different respect to subcutaneous adipose tissue (SAT), b respect to visceral adipose tissue (VAT), c respect to soleus, d respect to gluteus, e respect to heart and f respect to kidney, g respect to liver, τ respect to all.

* p < 0.05,

** p < 0.01,

and

*** p < 0.001.

**Figure 2:**
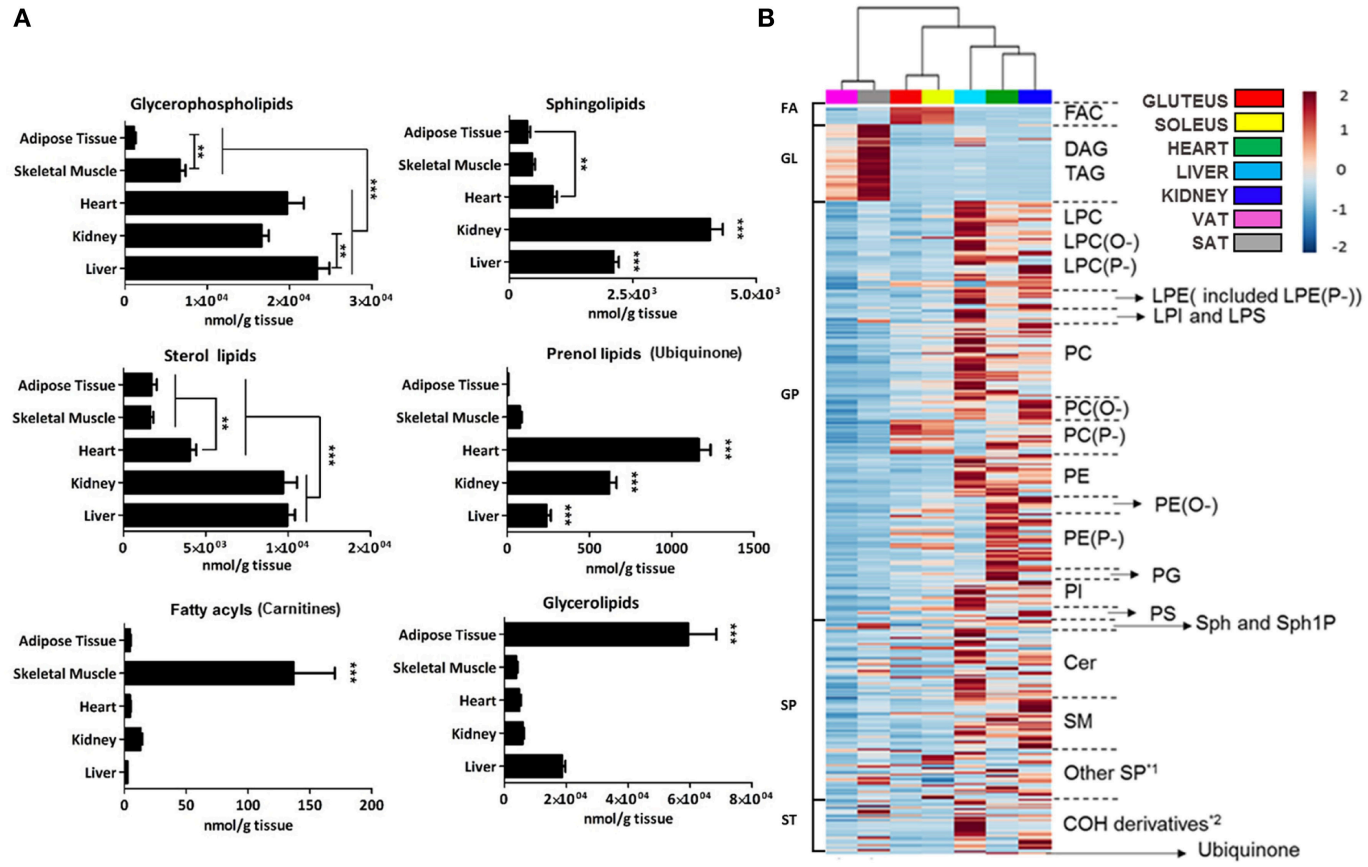
Lipid species concentration and distribution across the tissues analyzed by a targeted lipidomic approach. **(A)** Graph representation of average concentration in nmol/g of tissue of lipid species detected by targeted lipidomics grouped by families. Error bars denote SEM across 7–9 animals. Adipose tissue is comprised of visceral and subcutaneous tissue while gluteus and soleus comprised skeletal muscle. Kidney is represented only by the renal cortex. Statistical analysis performed was one-way ANOVA and *post-hoc* Tukey multiple test. **p < 0.01, and ***p < 0.001. **(B)** Heat map of abundance of the detected lipid species across samples. Each line represents one compound colored by its normalized abundance to internal standard, baseline and mean across samples. The scale from 2 to −2 represents the normalized abundance of each lipid in arbitrary units. *1 gangliosides and sulfatides; *2 hydroxycholesterol, desmosterol and cholesteryl esters.

To first obtain a high-level view of lipid tissue distribution, the concentration of each lipid category was represented for every mammalian tissue analyzed (Figure 2A). Further, in order to have a more graphical view a heat map clustering analysis, where both adipose tissues and skeletal muscles were treated as different tissues, was applied (Figure 2B).

**Figure 3:**
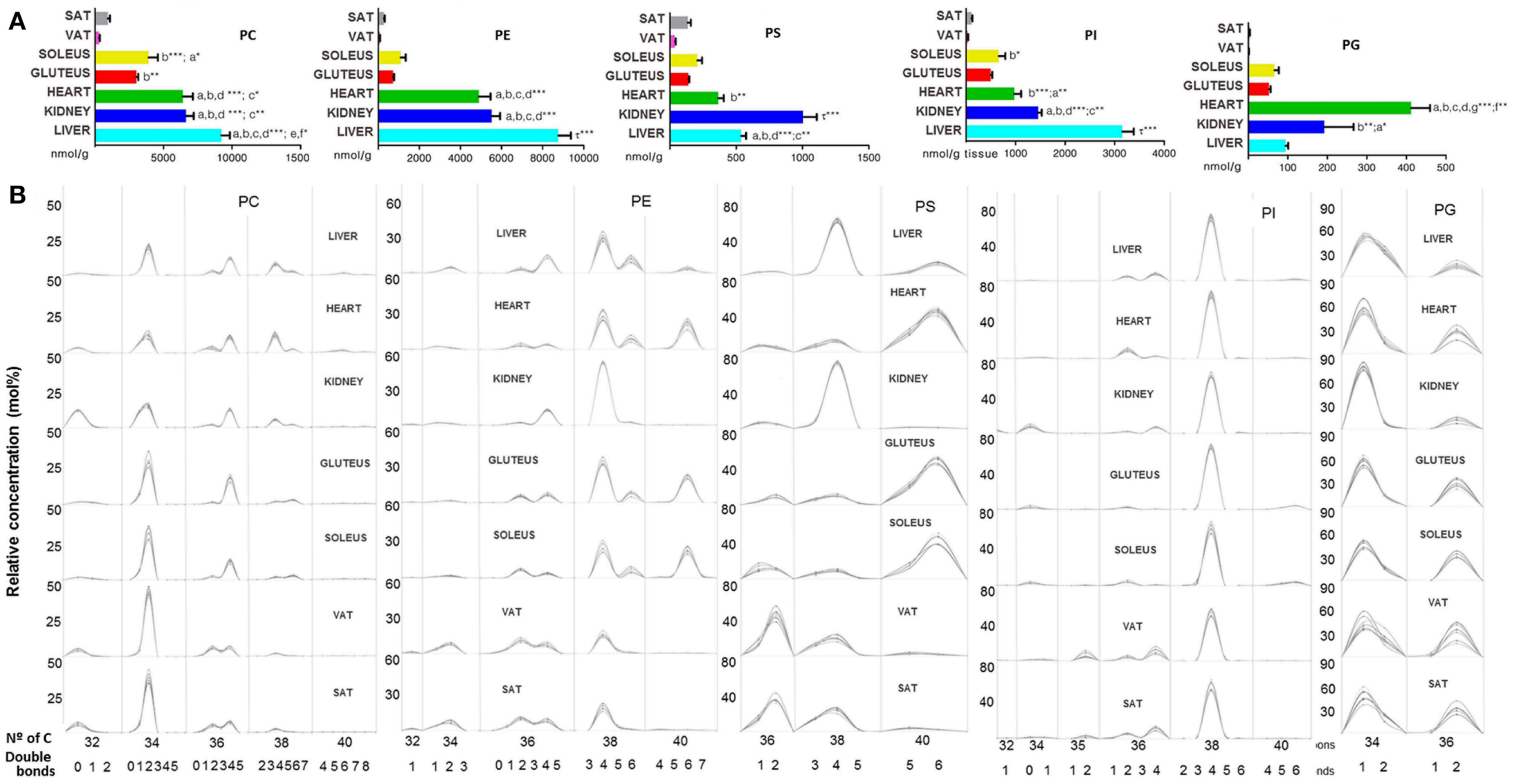
Glycerophospholipids within mammalian tissues detected by targeted lipidomic analysis. **(A)** Concentration of PC, PE, PS, PI, and PG subclasses. Values are expressed as mean ± SEM from 8 to 10 animals. Statistical analysis was one-way ANOVA and *post-hoc* Tukey significance is represented in the bar chart, meaning a significantly different respect to SAT, b respect to VAT, c respect to soleus, d respect to gluteus, e respect to heart and f respect to kidney, g respect to liver, τ respect to all. *p < 0.05, **p < 0.01, and ***p < 0.001. **(B)** Relative concentration of lipids normalized per sample to the total abundance within this lipid class to obtain molar fractions. Each solid line indicated tissue from an individual rat. Gray vertical lines separate lipids by total number of acyl chain carbons. The number of double bonds is indicated below within each group.

In the GP category, it can be seen a difference between on one hand heart, liver and kidney and on the other hand skeletal muscle and adipose tissue. Among heart, liver and kidney, the concentration of total GP detected only differed between liver and kidney. Moreover, skeletal muscle had a higher concentration of GP than adipose tissue (Figures 2A,B). By tissues, the total concentration of GP was: liver > heart > kidney > soleus > gluteus > SAT > VAT. Moreover, the concentration values shared by all tissues followed the next order: PC > PE > PI > PS. (Table 1). Glycerophosphocholines (PC) and ethanolamines (PE) were higher in liver, glycerophosphoglycerol species (PG) were more concentrated in heart, glycerophosphoinositol (PI) in liver and glycerophosphoserine (PS) in kidney (Figure 3A). Regarding the number of carbon atoms and unsaturation pattern of GPs, PC, PI, and PG showed a similar pattern for all the tissues while the pattern of PE and PS showed the maximum diversity amongst tissues (Figure 3B). The predominant PC molecular species among tissues were PC(16:0/18:2) and PC(16:0/18:1); for PE, PE(18:0/20:4) and PE(16:0/22:6); for PI, PI(18:0/20:4); and for PS, there were differences across tissues. PS(36:2) was for adipose tissue, PS(38:4) for liver and kidney and PS(40:6) for muscle tissues (heart, gluteus and soleus) (Supplementary Dataset 1).

**Figure 4:**
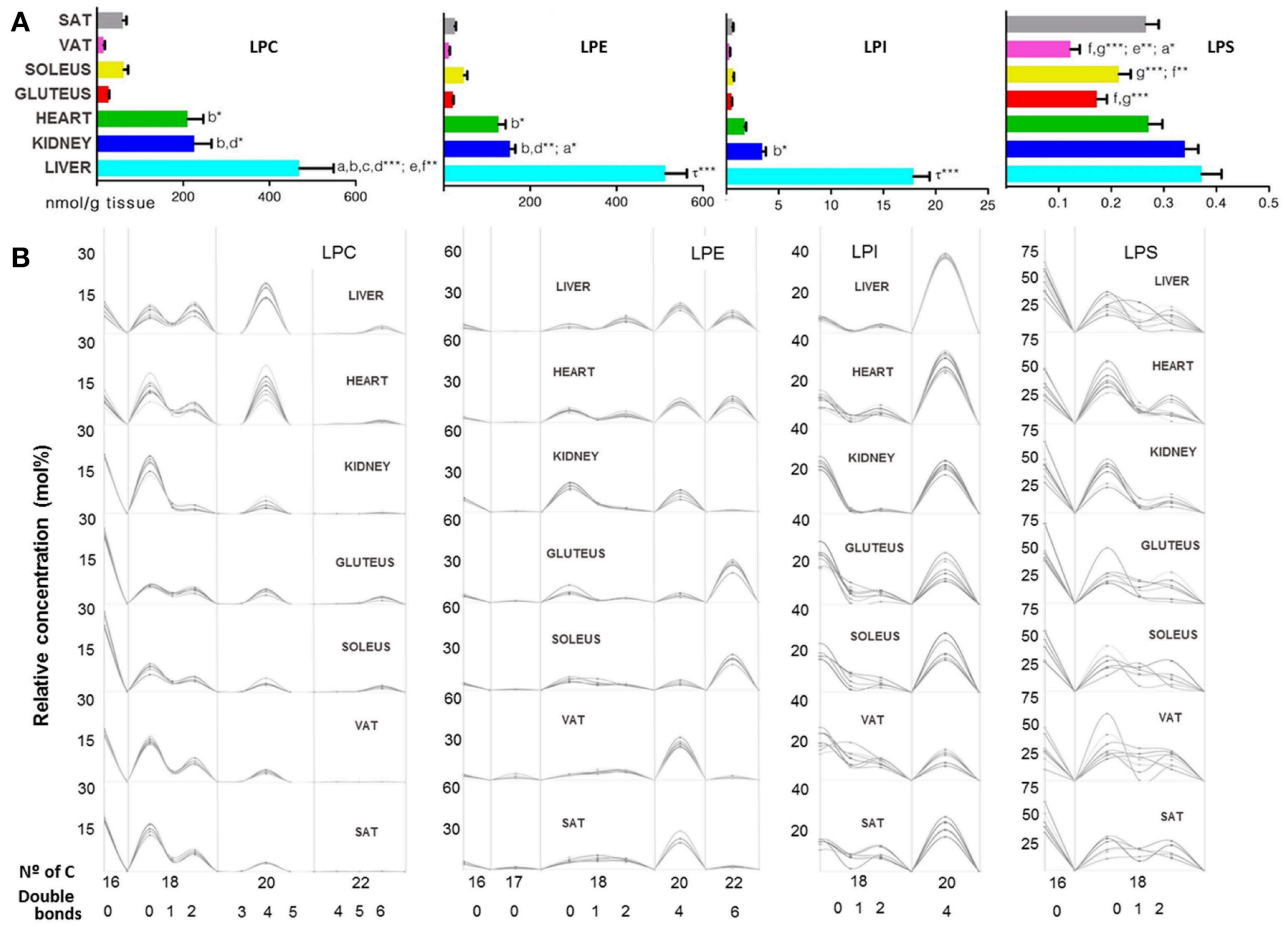
Lysoglycerophospholipids within mammalian tissues detected by targeted lipidomic analysis. **(A)** Concentration of LPC, LPE, LPS, and LPI subclasses. Values are expressed as mean ± SEM from 8 to 10 animals. Statistical analysis was one-way anova and *post-hoc* Tukey significance is represented in the bar chart, meaning a significantly different respect to SAT, b respect to VAT, c respect to soleus, d respect to gluteus, e respect to heart and f respect to kidney, g respect to liver, τ respect to all. *p < 0.05, **p < 0.01, and ***p < 0.001. **(B)** Relative concentration of lipids normalized per sample to the total abundance within this lipid class to obtain molar fractions. Each solid line indicated tissue from an individual rat. Gray vertical lines separate lipids by total number of acyl chain carbons. The number of double bonds is indicated below within each group.

Inside the GP, lysoglycerophospholipid species (LGP) of choline, ethanolamine, inositol and serine were detected and the concentration values shared by all tissues followed the next order: LPC > LPE > LPI > LPS; while by tissues, the total concentration of LGP was: liver > heart > kidney > soleus > gluteus > SAT > VAT (Table 1). Lysophosphatidylcholine (LPC) species were more concentrated in the heart, liver and kidney compared to the other tissues, in turn liver showed the highest concentration of LPC, except for LCP(P-) that was significantly higher in kidney. The rest of the lysophosphatidyl species were more concentrated in liver and kidney. SAT and VAT presented a different lysophosphoserine (LPS) concentration being higher in SAT along with the other tissues compared to VAT and skeletal muscle (Figure 4A). Focusing on liver, LPC, LPE, LPI concentrations were significantly higher compared to the other tissues followed by kidney and heart (Figure 4A). The (20:4) were the most common structural form of lysophospholipids in liver except for LPS, where the most abundant lipid was LPS(16:0). The LPG pattern of number of carbon and unsaturation was similar in every subclass between all tissues except for the LPE(22:6) higher in skeletal muscle, heart and liver respect to kidney and adipose tissue (Figure 4B).

**Figure 5:**
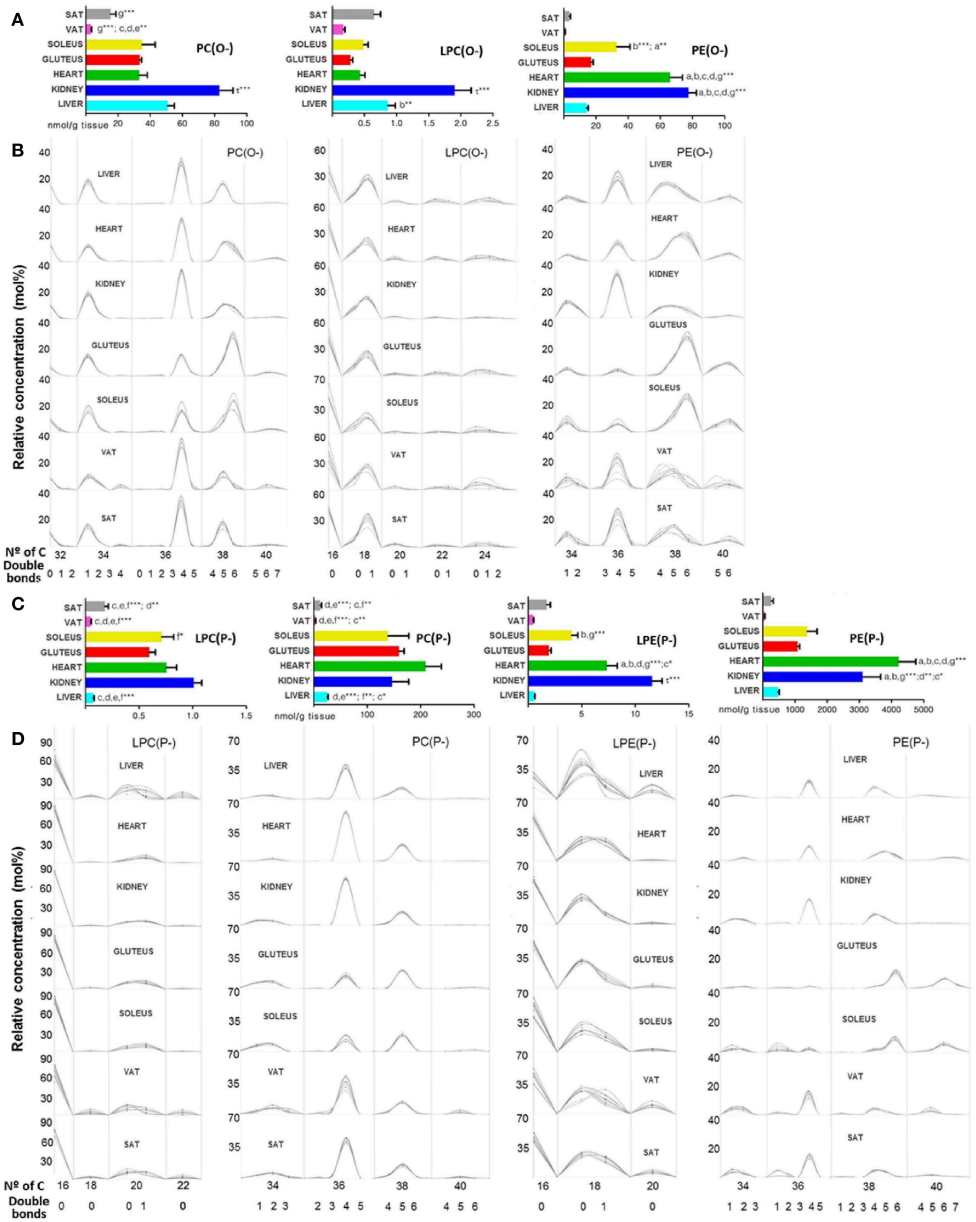
Ether lipid species within mammalian tissues detected by targeted lipidomic analysis. **(A,C)** Concentration of alkyl and alkenyl, respectively. Values are expressed as mean ± SEM from 8 to 10 animals. Statistical analysis was one-way anova and *post-hoc* Tukey significance is represented in the bar chart, meaning a significantly different respect to SAT, b respect to VAT, c respect to soleus, d respect to gluteus, e respect to heart and f respect to kidney, g respect to liver, τ respect to all. *p < 0.05, **p < 0.01, and ***p < 0.001. **(B,D)** Relative concentration of lipids normalized per sample to the total abundance within this lipid class to obtain molar fractions. Each solid line indicated tissue from an individual rat. Gray vertical lines separate lipids by total number of acyl chain carbons. The number of double bonds is indicated below within each group.

A great amount of ether lipids were detected inside the GP category as it can be seen in Figure 2B. In the present work, all ether lipids analyzed at tissue levels were present as PC and PE species. The 137 ether lipids detected were present in different concentration across tissues depending on their GP type or ether bond (alkyl or alkenyl) (Figure 5). Thus, the highest content was present in heart, where ether lipids represented 25.18% of the total GPs, followed by skeletal muscle (22.42% for gluteus and 20.86% for soleus), adipose tissue (15.90% for SAT and 11.55% for VAT), kidney (15.48%), and finally with the lowest content in liver where ether lipid represented 2.5% of total GPs (Table 1). The main chemical form is represented by ether lipids of PE with a content ranging from 80% in liver to 94% in heart of total ether lipids. According to the GP type, ether lipids of PE percentage of total amount of PE, show a relevant presence for all tissue (60.3% gluteus, 56.4% soleus, 48.8% SAT, 46.5% heart, 41.4% VAT, and 31.7% kidney), with the exception of liver where ether lipids of PE only represent about 5.4% of total PE in each tissue (Table 1). The most concentrated individual lipid species in all tissues were PE(P-16:0/20:4), PE(P-16:0/22:5n3), PE(P-16:0/22:6), PE(P-18:0/20:4), PE(P-18:0/22:6), and PE(P-18:1/20:4) (Supplementary Dataset 1). Regarding ether lipids of PC, their concentration of the total amount of ether lipids in each tissue was 0.3% in liver, 1.17% kidney, 1.35% heart, 3.33% gluteus, 2.27% soleus, 1.14% VAT, and 1.47% SAT (Table 1). And PC(P-16:0/20:4) and PC(P-16:0/22:6), followed by PC(P-16:0/16:0), PC(P-16:0/18:2), and PC(P-16:0/18:1) were the predominant molecular species (Supplementary Dataset 1). According to the ether bond, alkenyl species were more concentrated in skeletal muscle, especially those with choline. PE(P-) were more concentrated in heart and kidney, being LPE(P-) levels significantly higher in the renal cortex compared to the other tissues (Figures 5A–C). PC(P-36:4) and PE(P-36:4) were present in every tissue but less abundant in skeletal muscle, while PE(P-38:5) and PE(P-40:6) were the most abundant species in this tissue (Figure 5B). Regarding alkyl species detected, renal cortex presented significantly higher levels compared to the other tissues for those species with choline (Figures 5A–C). In the case of PE(O-), heart and kidney levels were significantly higher compared to the other tissues (Figure 5A). Inside both subclass, PC(O-) and PE(O-), different patterns across tissues were found. PC(O-36:4), PE(O-36:4), and PC(O-38:5) were present in every tissue but less abundant in skeletal muscle, while PC(O-38:6) levels were higher in skeletal muscle compared to the other tissues (dataset 1). PE(O-38:4) was abundant in liver and kidney, PE(O-38:5) in both types of adipose tissues, PE(O-38:6) in heart and skeletal muscle and the lack of PE(O-) with 40 carbon atoms was characteristics of kidney (Figure 5B). The forms alkyl- and alkenyl- lysoPC and lysoPE were minorities and all of them together represents the 0.006% in liver, 0.056% in kidney, 0.047% in heart, 0.048% in gluteus, 0.068% in soleus, 0.125% in VAT, and 0.137% in SAT respect the total amount of ether lipids in each tissue (Table 1). LPC(P-16:0) and LPE(P-16:0) were the most abundant species in every tissue except for liver, where LPE(P-18:0) was more abundant (Figure 5D).

**Figure 6:**
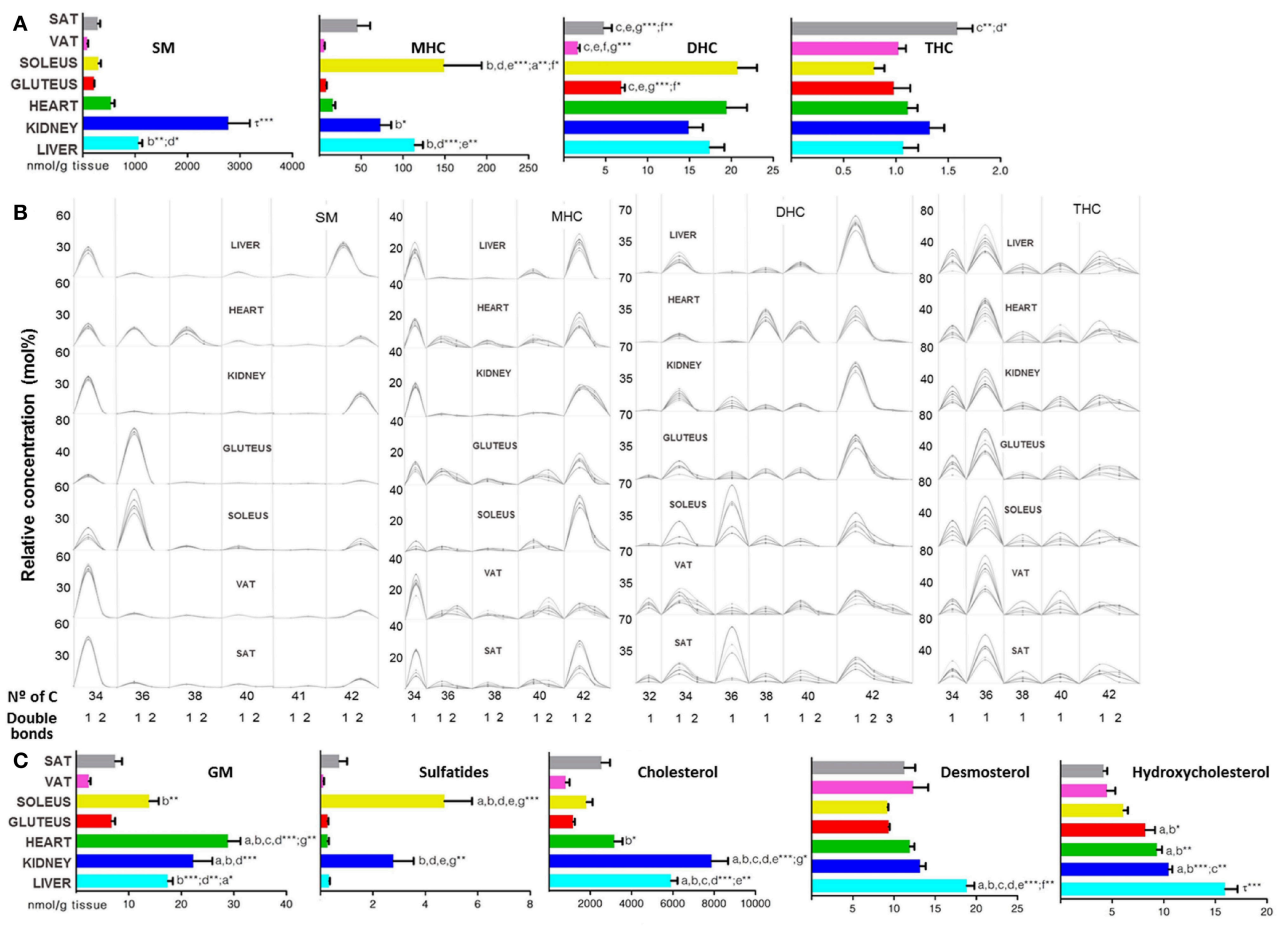
Structural sphingolipids and sterol lipids within mammalian tissues detected by targeted lipidomic analysis. **(A,C)** Concentration of SP and ST subclasses. Values are expressed as mean ± SEM from 8 to 10 animals. Statistical analysis was one-way anova and *post-hoc* Tukey significance is represented in the bar chart, meaning a significantly different respect to SAT, b respect to VAT, c respect to soleus, d respect to gluteus, e respect to heart and f respect to kidney, g respect to liver, τ respect to all. *p < 0.05, **p < 0.01, and ***p < 0.001. **(B)** Relative concentration of lipids normalized per sample to the total abundance within this lipid class to obtain molar fractions. Each solid line indicated tissue from an individual rat. Gray vertical lines separate lipids by total number of acyl chain carbons. The number of double bonds is indicated below within each group.

Cholesterol and other structural lipid from the SP catergory are represented in Figure 6. The total concentration of the lipid species analyzed from the SP category followed the next order by tissues: kidney>liver>heart>soleus>SAT>gluteus>VAT; being ceramides (Cer) more concentrated in liver and sphingomyelins (SM) in kidney (Table 1). The major sphingolipid in mammalian tissues was sphingomyelin (SM), with a range between 62 and 96% of total SP, followed by glycosphingolipids (monohexosylceramide, dihexosylceramide, and trihexosylceramide) (between 3 and 35%), gangliosides (GM) (0.5–5%), and finally sulfatides (0.03–0.9% of total SP) (Figure 6B and Supplementary Dataset 1). The percentage of SM concentration respect of the total SP concentration was higher in kidney (96.49% of total SP) followed by gluteus (90.44%), heart (89.59%) and VAT (88.81%), liver (87.67%), SAT(82.56%) and finally soleus (61.74%) (Table 1). The pattern of number of carbon atoms and unsaturation degree in kidney and liver was the same except for 42 carbon atoms species, being SM(42:1) characteristic of liver and SM(42:2) of kidney. The same pattern of number of carbon and unsaturation was found in both types of adipose tissue, being SM(34:1) the most abundant lipid species. The skeletal and cardiac muscle showed similar patterns although SM(38:1) was more concentrated in heart and SM(36:1) in skeletal muscle.

Inside the subclass of neutral glycosphingolipids, the concentrations across tissues changed depending on how many conjugated hexoses molecules the lipid contains. Monohexosylceramide (MHC) were significantly higher in soleus compared to the other tissues except for the hepatic tissue. The concentration of dihexosylceramide species (DHC) was significantly lower in both types of adipose tissue and gluteus while trihexosylceramide species (THC) were significantly more abundant in SAT compared only to both types of skeletal muscle (Figure 6A). Neutral glycosphingolipid composition seemed to be specific of each individual because of the heterogeneity on the abundance of the different lipid species across the animals. The patterns of number of carbon atoms and unsaturation degree for MHC and DHC species were different for each type of tissue while THC pattern was similar across tissues (Figure 6B). Regarding acidic glycosphingolipids, soleus and kidney presented the highest concentration of sulfatides, while gangliosides (GM) were more abundant in heart followed by kidney and liver (Figure 6C). However, the concentration of GM respect total SP was higher in all the muscle cells (heart, 4.72%; soleus, 2.84%; gluteus, 2.82%), followed by adipose tissue (2.42% for VAT, and 2.15% for SAT) than in liver (1.43%) and kidney (0.49%). This pattern changed for sulfatides being the percentage of this subclass respect to total SP concentration higher in soleus followed by adipose tissue (SAT>VAT) then gluteus, kidney, heart and finally liver (Table 1).

Cholesterol concentration was higher in kidney along with liver compared to the other tissues (from more to less, heart>SAT>soleus>gluteus>VAT). The cholesterol precursors and derivatives (desmosterol and hydroxycholesterol) were significantly higher in liver compared to the other tissues (Table 1 and Figure 6C).

**Figure 7:**
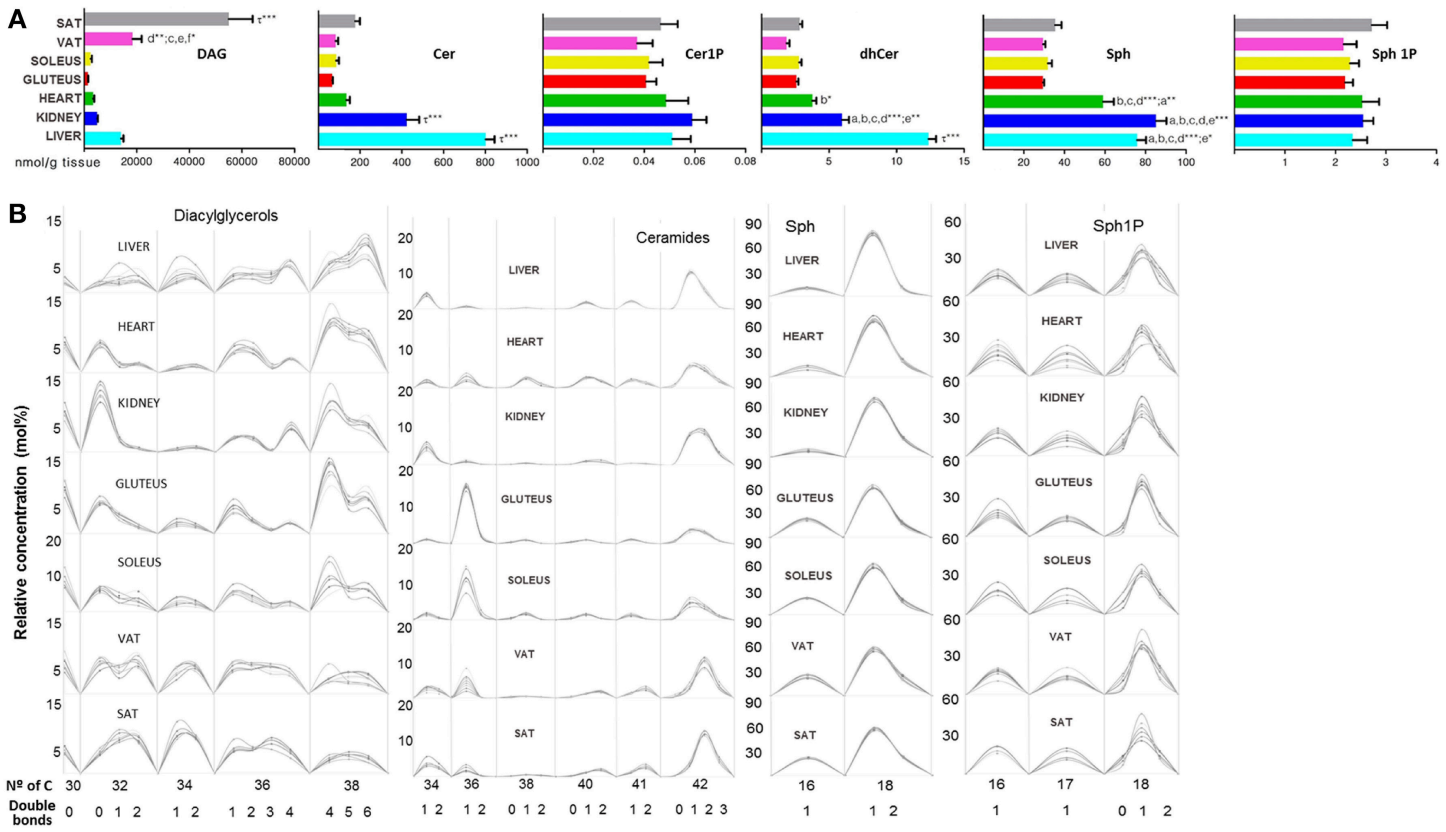
Second messenger lipid species within mammalian tissues detected by targeted lipidomic analysis. **(A)** Concentration of DAG, Cer, Cer1P, dhCer, Sph, and Sp1P. Values are expressed as mean ± SEM from 8 to 10 animals. Statistical analysis was one-way anova and *post-hoc* Tukey significance is represented in the bar chart, meaning a significantly different respect to SAT, b respect to VAT, c respect to soleus, d respect to gluteus, e respect to heart and f respect to kidney, g respect to liver, τ respect to all. *p < 0.05, **p < 0.01, and ***p < 0.001. **(B)** Relative concentration of lipids normalized per sample to the total abundance within this lipid class to obtain molar fractions. Each solid line indicated tissue from an individual rat. Gray vertical lines separate lipids by total number of acyl chain carbons. The number of double bonds is indicated below within each group.

Regarding lipid subclasses involved in signaling, DAG, Cer and other SP species are represented in Figure 7. DAG species were more concentrated in SAT than the other tissues while Cer were more concentrated in liver followed by kidney (Figure 7A). DAG from 30 carbons to 38 were equally distributed in both types of adipose tissue, while both DAG(38:6), DAG(18:2/20:4) and DAG(18:0/22:6), were particularly abundant in liver, DAG(16:0/16:0) in kidney as well as DAG(18:0/20:4), which also had high levels in cardiac and skeletal muscle (Figure 7B and Table 1). Ceramides, including dihydroceramides, were significantly higher in liver followed by kidney compared to the other tissues (Figure 7A). The Cer subclass showed the same pattern of number of carbon and unsaturation for VAT and SAT, whereas, skeletal muscle presented higher levels of Cer(36:1) and along with heart both presented lower levels of Cer(38:1) (Figure 7B). The species conjugated with phosphate did not show differences across tissues while sphingosine (Sph) levels were significantly higher in the heart, liver and kidney compared to the adipose tissue and skeletal muscle (Figure 7A). In both, Sph and Sph1P, the 18:1 species were the most abundant and the pattern composition was the same in all the tissues (Figure 7B).

**Figure 8:**
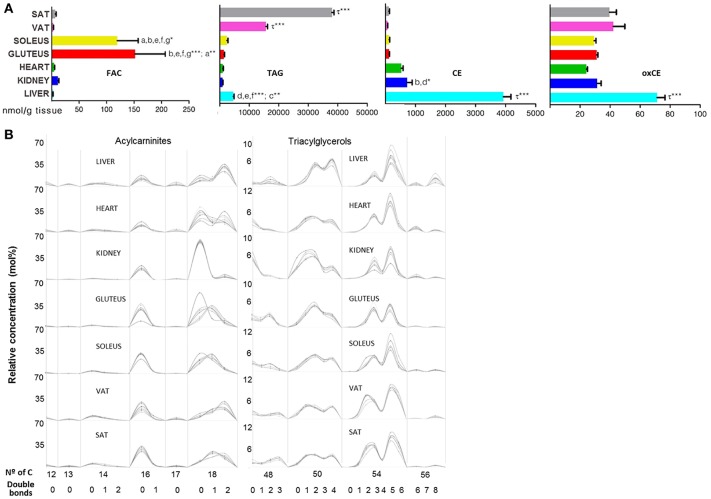
Bioenergetic lipid species within mammalian tissues detected by targeted lipidomic analysis. **(A)** Concentration of FAC, TAG, CE, and oxCE. Values are expressed as mean ± SEM from 8 to 10 animals. Statistical analysis was one-way anova and *post-hoc* Tukey significance is represented in the bar chart, meaning a significantly different respect to SAT, b respect to VAT, c respect to soleus, d respect to gluteus, e respect to heart and f respect to kidney, g respect to liver, τ respect to all. *p < 0.05, **p < 0.01, and ***p < 0.001. **(B)** Relative concentration of lipids normalized per sample to the total abundance within this lipid class to obtain molar fractions. Each solid line indicated tissue from an individual rat. Gray vertical lines separate lipids by total number of acyl chain carbons. The number of double bonds is indicated below within each group.

To end with lipid species involved in energy storage, acylcarnitines (FAC), TAG and CE across mammalian tissues are represented in Figure 8. The 14 species of FAC that represent the FA category were present at higher concentrations in skeletal muscle compared with the other tissues (Figure 8A). The number of carbon atoms and the degree of unsaturation of the FAC was similar between tissues, with the long chain species being more abundant in particular (18:0) in the kidney (Figure 8B and Supplementary Dataset 1). A similar situation was observed in the TAG subclass, in which adipose tissue presented higher concentration compared with the other tissues (Figure 8A). Further, it can be seen how TAG abundance was higher in SAT rather than VAT (Figure 8A). Polyunsaturated TAG with 50 and 54 carbons were abundant in every tissue analyzed. The configuration of number of carbon atoms and unsaturation degree was similar in every tissue, especially between both types of adipose tissue (Figure 8B). Regarding CE and oxCE species, the highest concentration was found at hepatic level, followed by kidney for the CE species while the oxidized forms of CE were higher in liver compared to the rest of the tissues where the concentration of oxCE was similar (Figure 8A).

### Distribution of lipid species within tissues

**Figure 9:**
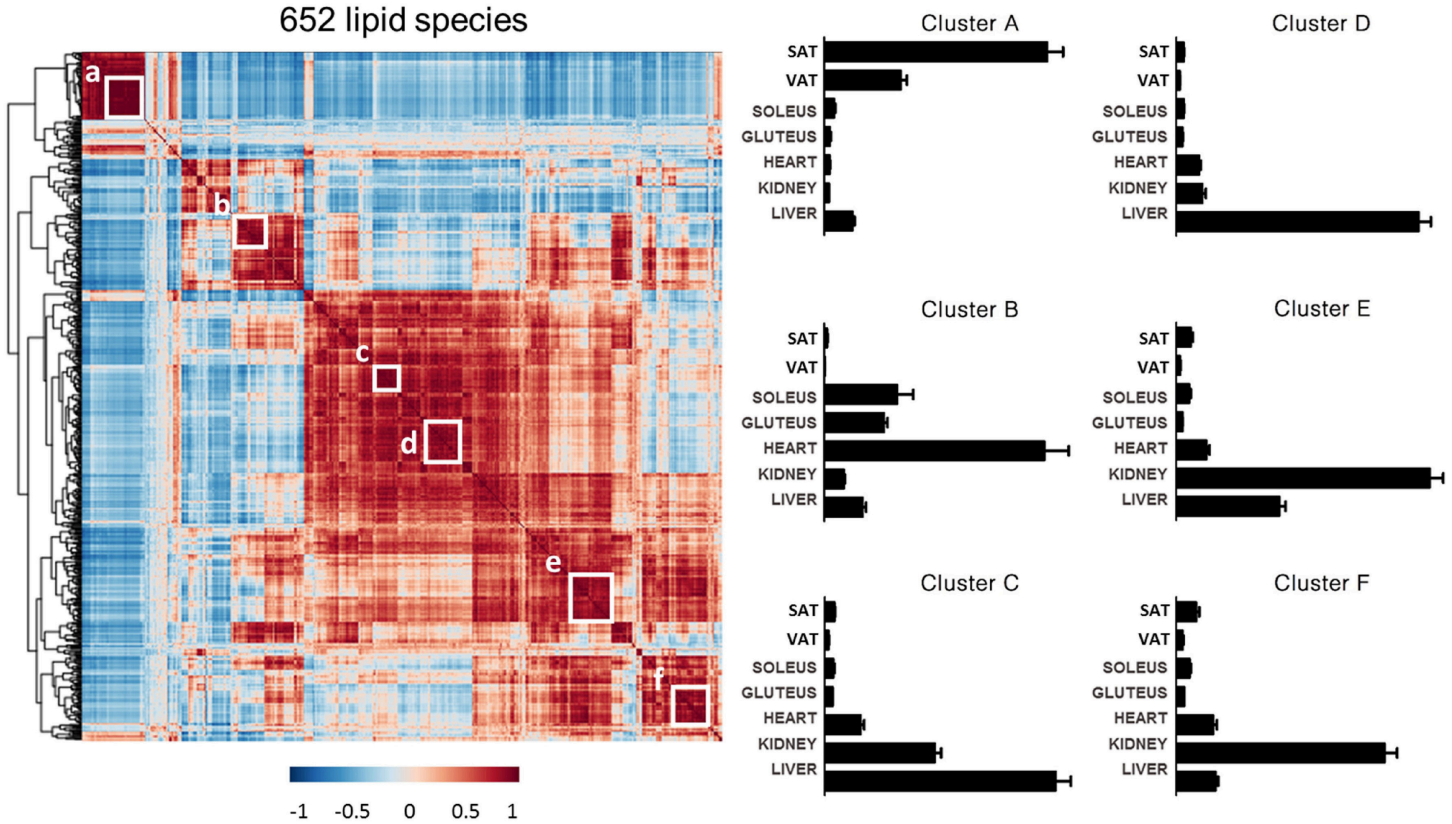
Cluster analysis of lipid profiles. **Left:** heat map of Pearson correlation matrix across the 652 targeted lipids with corresponding hierarchical tree. White boxes indicate lipid clusters chosen for further analysis. **Right:** Concentration of lipid clusters across all the tissues analyzed. For the information about the lipid species in each lipid cluster see Table S3.

Complementary to the information above, the lipid distribution within each tissue has been analyzed. All of the species detected in the targeted lipidomic analysis (652 species) were jointly analyzed by a Pearson correlation and the resulting matrix was organized by hierarchical clustering (Figure 9). This analysis revealed several clusters of lipids with similar tissue abundance patterns and six clusters have been deeper studied. First cluster, named (a) was consisted of GL, mostly TAG, and one oxidized cholesteryl ester. This cluster was almost exclusively present in adipose tissue, especially in SAT. Cluster (b) was formed by 27 species where the 66.66% of them were ether lipids, in particular PE(P-). These species were particularly abundant in cardiac muscle as well as in the skeletal muscle although not exclusively. The other clusters were mostly comprised by SP species. Cluster (c) and (d) had species with higher concentration in liver. SM was the most abundant lipid subclass in cluster (c) although PE, PI, Cer and CE were present. On the other hand, Cer was the most abundant subclass in cluster (d) followed by LPE, LPI, PE, PI and SM. Both clusters seem to contain lipid species important for signaling, physiological functions and metabolism of the liver. To end with, similarly to this previous cluster, (e) and (f) clusters could be combined as a renal cortex lipid species representation. In both of them, SM was the most abundant subclass followed by several alkylglycerophospholipids, mostly from choline but also with ethanolamine. Composition of each cluster is specified in Table S3.

## Discussion

In biological systems, the primary function of lipids is to generate membranes. It was later during evolution that this property was extended to new functions such as cell signaling and energy storage. To do this, a wide diversity of molecular lipid species emerged and their biosynthesis pathways incorporated to the general cell metabolism. This lipid diversity was also expressed at the compositional level in cell membranes and organelles. The present study demonstrates the existence of a tissue-specific fingerprint, which may be because of the specific metabolic adaptations of each tissue. All the tissues analyzed are predominantly (about 70–80%) composed by one cell type (cardiomyocyte, adipocyte, hepatocyte, etc.) but the potential effect of the other cell populations in the whole lipidome should not be dismissed.

The major structural lipids in membranes from eukaryotic cells are GPs: PC, PE, PS, and PI (Van Meer et al., 2008). In accordance with this general idea, our results confirm their quantitative relevance in all the tissues. By tissues, the relative abundance is: liver > heart > kidney > soleus > gluteus > SAT > VAT, probably as expression of differences in the complexity of cellular metabolism and the relative abundance and traffic of organelles and membranes. Besides, the relative abundance shared by all tissues is: PC > PE > PI > PS. PC accounts for >50% of the membrane glycerophospholipids for all tissues, analogously to most eukaryotic membranes. Although the percentage of PI and PS in the different tissues is low it is important to emphasize the relevance of these species because their important role acting as signaling molecules via interactions with specific proteins. Additionally, it is interesting to verify the significant differences that exist between the different types of skeletal muscle (gluteus vs. soleus), as well as adipose tissue (visceral vs. subcutaneous).

The most abundant PC and PE molecular species shared by all tissues are or primary molecular species in the *de novo* synthesis of PC and PE [PC(16:0/18:1), PC(16:0/18:2), PE(16:0/18:2) and PE(16:0/22:6)] (Schmid et al., 1995a); or are produced by a remodeling pathway at both sn-1 and sn-2 positions [PC(16:0/16:0), PC(18:0/18.2), PC(16:0/20:4), PE(16:0/20:4) and PE(18:0/20:4)] (Schmid et al., 1995b; Hishikawa et al., 2014). For PI and PS, the most significant molecular species [PI(16:0/20:4), PI(18:0/20:4) and PS(38:4)] are also resulting from a remodeling activity and with arachidonic acid (20:4) as the most predominant acyl chain found in the sn-2 position. The remaining molecular species are the result of remodeling processes and their concentration is in a lower range showing a wide diversity of incorporated polyunsaturated fatty acids.

In this sense, there are differences among GPs in terms of chain length (number of carbon atoms) and degree of unsaturation (number of double bonds) in their fatty acid composition, the major factors determining the geometric properties of lipids which, in turn, have major consequences on the membrane functional properties (Piomelli et al., 2007). Thus, the predominant PC molecular species among tissues show a chain length (as sum of both fatty acids) of 34 carbon atoms, with an average in the unsaturation degree around 2; for PE, 38 carbon atoms and unsaturation degree of 4–6; for PI, 38 carbon atoms, and 4 double bonds; and for PS, 36–40 carbon atoms and 4–6 double bonds. The major diversity in number of carbon atoms and unsaturation degree among tissues was observed for PE and PS, both subclasses showed the greatest diversity in the polyunsaturated fatty acid composition and clear differences among tissues.

The GP phosphatidylglycerol (PG) is a precursor of cardiolipin, a very special and unique glycerophospholipid which is found at mitochondrial level (Van Meer, 2005; Van Meer et al., 2008). In our study, PG is detected in all tissues, being its relative abundance especially lower in both adipose tissues, slightly higher in skeletal muscle tissues, kidney and liver, and showing the highest level in heart, clearly expressing the mitochondrial enrichment of this tissue. In all tissues, PG(16:0/18:1) and PG(16:0/18:2) predominates, probably expressing molecular species from *de novo* synthesis; followed by PG(18:1/18:1), resulting from remodeling. This lipid specie is the chemical form used for cardiolipin synthesis since it has been demonstrated that only fatty acids with 18 carbon atoms, and low unsaturation degrees (1 or 2 double bonds) are present in mitochondrial cardiolipin from rat tissues (Schlame et al., 2000; Horvath and Daum, 2013).

An especial mention is deserved for the prenol lipid ubiquinone (also named coenzyme Q) which is primarily present also in mitochondria as component of the electron transport chain. Ubiquinone participates in aerobic cellular respiration and it seems that also have a potential antioxidant role (Wang and Hekimi, 2016). Similarly to PG content, ubiquinone show the highest concentrations in those tissues with the highest energy requirements —such as heart, kidney, and liver—, decreasing their content in one order of magnitude in skeletal muscle, and 2 orders for adipose tissue.

Glycerophospholipid acyl chains are remodeled by the regulated activity of different enzymes like phospholipase As, acyl-CoA synthases, transacylases, and lysophospholipid acyltransferases (Hishikawa et al., 2014). The result is the generation of a pool of lysoglycerophospholipids (LGPs). The relative abundance of LGPs is, as expected, lower than GPs in at least 1.5 orders of magnitude, and maintain the relation among them in an identical way to GPs. Thus, LPC > LPE > LPI > LPS; and by tissues: liver > heart > kidney > soleus > gluteus > SAT > VAT, that express tissue-specific differences is the rate of remodeling according to the GP classes, but also the diversity of molecular GPs species generate, being highest for LPC, then LPE, and finally LPI and LPS.

Focusing on the LGPs species with the highest concentrations in the tissues, two groups can be discerned. The first one is made up by LGPs with the fatty acid in sn-1 position, generated by the activity of a phospholipase A2 [LPC(16:0), LPE(16:0), LPE(18:0) and LPS(16:0)]. This group share the common trait of having a saturated fatty acid in their structure. They are likely generated within the remodeling process of GPs. In contrast, the second group is made up by LGPs with the fatty acid in sn-2 position [LPC(18:2), LPC(20:4), LPE(18:2), LPE(20:4), LPE(22:6), and LPI(20:4)]. Interestingly, the systematic presence of highly unsaturated fatty acids suggests that all of them are generated from remodeled GPs previously obtained by *de novo* synthesis. Furthermore, their generation implies the activity of a phospholipase A1, which has to be present in all tissues, suggesting that the resulting LGPs are not transition species in the remodeling process, but the result of a specific pathway to generate a new subclass of compounds. In fact, recently it has been described that LGPs sn-2 are substrates for a new lipid signaling pathway based on the generation and activity of specific lipid species named eicosanoids-lysolipids (Liu et al., 2016) and, we additionally also propose, docosanoids-lysolipids.

Ether lipids are a subclass of GPs that have an alkyl chain attached by an ether bond at the sn-1 position of the glycerol backbone (Dean and Lodhi, 2018). Most ether lipids are presented as PC and PE molecular species. In the present work, all ether lipids analyzed at tissue levels are present as PC and PE species. Our data demonstrate that ether lipids have a heterogeneous distribution depending on the tissue. Thus, the highest content is present in heart, followed by skeletal muscle, adipose tissue, kidney, and finally liver, being the main chemical form PE(P-). The main molecular species of PE(P-) shared by all tissues [PE(P-16:0/20:4), PE(P-16:0/22:5n3), PE(P-16:0/22:6), PE(P-18:0/20:4), PE(P-18:0/22:6) and PE(P-18:1/20:4)] confirm that in PE(P-) the long chain fatty acid in sn-1 consists exclusively of saturated and monounsaturated groups, while sn-2 position is esterified predominantly with n-6 and n-3 polyunsaturated fatty acids. Interestingly, this fatty acid profile confers a higher unsaturation degree and average chain length to ether lipids present as PE than as PC. In contrast to PE(P-), the PC(P-) percentage of total amount of PC is in a low range for all the tissue. Finally, the forms alkyl- and alkenyl- lysoPC and lysoPE are quantitatives minorities.

The physiological role of plasmalogens are essentially linked to their function as membrane components contributing to important properties such as fluidity, formation of lipid raft microdomains, and source of second messengers. Other specific functions where plasmalogens are involved are transmembrane protein function, cholesterol transport, vesicular function, membrane fusion events, and G-protein mediated signal transduction (Dean and Lodhi, 2018). Interestingly, an antioxidant effect has also been ascribed to plasmalogens that, like a scavenger, could protect unsaturated membrane lipids. Consequently, it is proposed that the heterogeneous presence of plasmalogens in tissues is an adaptive response to offer stability and protection against oxidative stress conditions to lipid membranes, and particularly lipid rafts, in a tissue-dependent way.

The sphingolipids and cholesterol constitute other classes of structural lipids (Van Meer et al., 2008). Our data confirm that the major sphingolipid in mammalian tissues is sphingomyelin (SM), followed by glycosphingolipids, gangliosides (GM) and finally sulfatides. For all of them, the distribution follows a heterogeneous and non-shared pattern. Thus, SP total amount is highest in kidney, followed by liver, heart, soleus, SAT, gluteus, and finally VAT. The predominant lipid species are SM(d18:1/16:0) and SM(d18:1/24:0). For glycosphingolipids, the tissue amount expresses, for all tissues, a gradient being highest for the MHC forms, followed by DHC, and finally THC with the lowest content. Among tissues, it is remarkable the exceptional amount of MHC showed by soleus (30%). For GM, the relative abundance predominates in muscle cells, followed by adipose tissue, and then liver and kidney. Finally, sulfatides show the lowest relative abundance and, for tissues (from more to less): soleus > SAT > VAT > gluteus > kidney > heart > liver.

Globally, all SP are formed by saturated and monounsatureated fatty acids that confer to sphingolipids a geometry that contributes, jointly with cholesterol, to lipid microdomain formation probably affecting the membrane biophysical properties such as microviscosity in a tissue-specific way. Otherwise, further analyses will be performed to establish the link between lipid profile and biophysical properties for each tissue.

For tissue cholesterol, the highest content is present in kidney, followed by liver, heart, SAT, soleus, gluteus, and VAT. Interestingly, this gradient is shared for total amount of SP, likely as expression of the close interaction between cholesterol and SP species to maintain the optimal properties of the membrane. The precursor in the cholesterol biosynthesis desmosterol shows the highest concentration in liver, being practically identical for the other tissues; and hydroxycholesterol, a cholesterol metabolite, shows a homogeneous distribution across tissues, although the highest concentration is again found at hepatic level.

The hydrolysis of glycerolipids and sphingolipids produces a series of messenger lipids which play a key role in cell signaling such as lysophosphatidylcholines (LPC), diacyglycerols (DAGs), sphingosines (Sph), sphingosine-1-phosphate (Sph1P), ceramide-1-phosphate (Cer1P), dihydroceramides (dhCer) and ceramides (Cer), analyzed in the present study. It is important to mention, however, that other functional properties ascribed to these lipids (i.e., precursor of other lipid classes and/or structural role in membranes) cannot be discarded. In any case, for all tissues, in terms of relative abundance, the highest amounts correspond to DAGs, followed by ceramides, LPCs, sphingosines, dihydroceramides, Sph1Ps and Cer1P. More in detail, DAGs are particularly relevant in adipose tissue (SAT > VAT) where there are the highest amount along with liver, then kidney, and finally the muscular tissues (heart > soleus > gluteus). For Cer, and their precursor dihydroceramides, the highest amount is present in liver and kidney, being their amount lower and identical for the rest of tissues. In a similar way, LPCs are especially present in liver, kidney and also heart, sharing skeletal muscle and adipose tissues the lowest content. Sph were higher in heart, kidney and liver while no differences across tissues were observed for Sph1P and Cer1P.

Finally, lipids are used for energy storage as TAGs and CEs, in lipid droplets, and energy source principally as FACs. For FACs, the highest levels were detected in skeletal muscle (both gluteus and soleus), and the lowest, and in a similar range, in the other tissues (sorted by relative abundance: kidney > SAT > heart > VAT > liver). TAGs are the main form of energy storage. As expected, the highest content is present in adipose tissues (SAT > VAT), followed by kidney, liver, soleus, gluteus and finally heart which present a content significantly lower in an order of magnitude. In line with this distribution, and described for the first time, we have detected the presence in significant amounts of ether lipids-TAGs [TAG(O-)]. Considering that ether lipids have, among others, antioxidants properties, we propose that the present of this form of TAGs and their distribution across tissues is a molecular adaption to protect lipid droplets from cellular oxidative conditions. In addition to TAG, the other chemical form of energy storage is as CEs. Liver is, by far, the tissue with the highest content in CE, followed by kidney and heart, and showing the lowest content and with a similar amount the skeletal muscle (both gluteus and soleus) and adipose (both SAV and VAT) tissues. Interestingly, the content of oxidized forms of cholesteryl esters (oxCE) are also highest in liver, and lower and not different in the other tissues.

All in all, our findings suggest the presence of general rules and patterns shared by rat tissues about lipid distribution as a result of an evolutionary and developmental process to cover three basic cellular processes: formation of membranes, cell signaling and bioenergetics. The first rule is the preferential structural presence of GPs with a specific weight for PCs and PEs followed by PI and PS; and, in a minor amount, the presence of SP with a predominant use of SMs. Interestingly, the important presence of ether lipid forms and fatty acids with a low unsaturation degree, both traits conferring antioxidant and protective properties to membranes, could be the expression of an evolutionary adaption to oxidative stress to confer a resistance to damage in order to maintain membrane integrity (Pamplona, 2008). A second general rule is the predominance of molecular species obtained by *de novo* synthesis along with a minor presence of a wide variety of other lipid species which offer diversity in the fatty acid profile (particularly to ensure a diversity in the polyunsaturated fatty acid content). It is suggested that lipid species are built on the basis of a resistance to oxidative stress since saturated and monounsaturated fatty acids are the primary lipid used for their synthesis. It is a remodeling process who generates a diversity of lipid species and, consequently, changes in the susceptibility to oxidative damage. And as a third rule, the differences among tissues can be ascribed to quantitative rather than qualitative differences in the lipid species used that could be interpreted as an adaption to the specific metabolic and physiological needs which are tissue-dependent. In conclusion, our lipidomic approach demonstrates the existence of a specific lipid distribution among tissues. However, with the present data we cannot draw conclusions about the metabolic differences between tissues. Further fluxomic analyses should be addressed to describe the metabolic rates of lipid metabolism for each tissue. Moreover, additional “omics” studies such as proteomics and transcriptomics should be done for a more complete metabolic network description which support the differential lipid distribution among tissues.

## Author contributions

PM and RP designed the study. IP, KH, RC, VA, and MJ performed experimental work. IP, MJ, and RP analyzed the data. RP supervised the design and data interpretation. The manuscript was written by IP, PM, MJ, and RP and edited by RP. All authors discussed the results and commented on the manuscript.

### Conflict of interest statement

The authors declare that the research was conducted in the absence of any commercial or financial relationships that could be construed as a potential conflict of interest.
