# Supplementary material for: Lipidomics Reveals a Tissue-Specific Fingerprint
Source: Front Physiol. 2018 Aug 28;9:1165. doi: 10.3389/fphys.2018.01165 (PMC6121266; doi:10.3389/fphys.2018.01165)
Supplement: Supplementary file 1 [file Table_1.docx]

Supplementary Material

**Lipidomics reveals tissue-specific organization of lipids**

Irene Pradas,^1^ Kevin Huynh,^2^ Rosanna Cabré,^1^ Victòria Ayala,^1^ Peter J Meikle,^2^ Mariona Jové,^1^* and Reinald Pamplona^1^*

^1^Department of Experimental Medicine, University of Lleida-Institute for Research in Biomedicine of Lleida (UdL-IRBLleida), E-25198 Lleida, Spain

^2^Baker Heart and Diabetes Institute, Melbourne VIC 3004, Australia

*** Correspondence:**Dr. Mariona Jové, Departament de Medicina Experimental, Universitat de Lleida-Institut de Recerca Biomedica de Lleida (IRBLleida), Edifici Biomedicina 1, Av. Alcalde Rovira Roure-80, Lleida 25198, Catalonia, Spain. Phone: (+34)973702442

[mariona.jove@udl.cat](mailto:mariona.jove@udl.cat)

Prof. Dr. Reinald Pamplona, Departament de Medicina Experimental, Universitat de Lleida-Institut de Recerca Biomedica de Lleida (IRBLleida), Edifici Biomedicina 1, Av. Alcalde Rovira Roure-80, Lleida 25198, Catalonia, Spain. Phone: (+34)973702442

[reinald.pamplona@mex.udl.cat](mailto:reinald.pamplona@mex.udl.cat)

# Supplementary Tables

**Table S1.** Class representative and extraction internal standards added to the samples in untargeted and targeted lipidomics analysis.

| COMPOUND | SOURCE | IDENTIFIER |
| --- | --- | --- |
| Untargeted Lipidomic Analysis | | |
| 1,3(d5)-dihexadecanoyl-glycerol | Avanti Polar Lipids | 110537 |
| 1,3(d5)-dihexadecanoyl-2-octadecanoyl-glycerol | Avanti Polar Lipids | 110543 |
| 1-hexadecanoyl(d31)-2-(9Z-octadecenoyl)-sn-glycero-3-phosphate | Avanti Polar Lipids | 110920 |
| 1-hexadecanoyl(d31)-2-(9Z-octadecenoyl)-sn-glycero-3-phosphocholine | Avanti Polar Lipids | 110918 |
| 1-hexadecanoyl(d31)-2-(9Z-octadecenoyl)-sn-glycero-3-phosphoethanolamine | Avanti Polar Lipids | 110921 |
| 1-hexadecanoyl-2-(9Z-octadecenoyl)-sn-glycero-3-phospho-(1'-rac-glycerol-1',1',2',3',3'-d5) | Avanti Polar Lipids | 110899 |
| 1-hexadecanoyl(d31)-2-(9Z-octadecenoyl)-sn-glycero-3-phospho-myo-inositol | Avanti Polar Lipids | 110923 |
| 1-hexadecanoyl(d31)-2-(9Z-octadecenoyl)-sn-glycero-3-[phospho-L-serine] | Avanti Polar Lipids | 110922 |
| 26:0-d4 Lyso PC | Avanti Polar Lipids | 860389 |
| 18:1 Chol (D7) ester | Avanti Polar Lipids | 111015 |
| cholest-5-en-3ß-ol (d7) | Avanti Polar Lipids | LM-4100 |
| D-erythro-sphingosine-d7 | Avanti Polar Lipids | 860657 |
| D-erythro-sphingosine-d7-1-phosphate | Avanti Polar Lipids | 860659 |
| N-palmitoyl-d31-D-erythro-sphingosine | Avanti Polar Lipids | 868516 |
| N-palmitoyl-d31-D-erythro-sphingosylphosphorylcholine | Avanti Polar Lipids | 868584 |
| Octadecanoic acid-2,2-d2 | Sigma Aldrich | 19905-58-9 |
| Targeted Lipidomic Analysis | | |
| N-heptadecanoyl-D-erythro-sphingosine | Avanti Polar Lipids | 860517 |
| Cholesteryl-2,2,3,4,4,6-d6 Octadecanoate | CDN Isotopes | D-5823 |
| N-octanoyl-D-erythro-sphinganine | Avanti Polar Lipids | 860626 |
| 1-tridecanoyl-2-hydroxy-sn-glycero-3-phosphocholine | Avanti Polar Lipids | 855476 |
| 1,2-ditridecanoyl-sn-glycero-3-phosphocholine | Avanti Polar Lipids | 850340 |
| 1,2-diheptadecanoyl-sn-glycero-3-phosphoethanolamine | Avanti Polar Lipids | 830756 |
| 1-myristoyl-2-hydroxy-sn-glycero-3-phosphoethanolamine | Avanti Polar Lipids | 856735 |
| 1,2-diheptadecanoyl-sn-glycero-3-phospho-(1'-rac-glycerol) | Avanti Polar Lipids | 830456 |
| 1,2-diheptadecanoyl-sn-glycero-3-phospho-L-serine | Avanti Polar Lipids | 840028 |
| 1-tridecanoyl-2-hydroxy-sn-glycero-3-phospho-(1'-myo-inositol) | Avanti Polar Lipids | 850101 |
| N-(dodecanoyl)-sphing-4-enine-1-phosphocholine | Avanti Polar Lipids | LM-2312 |
| D-erythro-sphingosine (C17 base) | Avanti Polar Lipids | 860640 |
| Glyceryl triheptadecanoate | Sigma Aldrich | T2151 |
| 1,2-dipentadecanoyl-sn-glycerol | Sigma Aldrich | P7285 |
| Cholest-5-en-3ß-ol(d7) | Avanti Polar Lipids | LM-4100 |
| D-glucosyl-ß-1,1'-N-palmitoyl-D-erythro-sphingosine | Avanti Polar Lipids | 860539 |
| D-lactosyl-ß-1,1' N-palmitoyl-D-erythro-sphingosine | Avanti Polar Lipids | 860576 |
| Trihexosylceramide 17:0 | Avanti Polar Lipids | 860646 |
