# Supplementary material for: Lipidomics Reveals a Tissue-Specific Fingerprint
Source: Front Physiol. 2018 Aug 28;9:1165. doi: 10.3389/fphys.2018.01165 (PMC6121266; doi:10.3389/fphys.2018.01165)
Supplement: Supplementary file 2 [file Table_2.docx]

Supplementary Material

**Lipidomics reveals tissue-specific organization of lipids**

Irene Pradas,^1^ Kevin Huynh,^2^ Rosanna Cabré,^1^ Victòria Ayala,^1^ Peter J Meikle,^2^ Mariona Jové,^1^* and Reinald Pamplona^1^*

^1^Department of Experimental Medicine, University of Lleida-Institute for Research in Biomedicine of Lleida (UdL-IRBLleida), E-25198 Lleida, Spain

^2^Baker Heart and Diabetes Institute, Melbourne VIC 3004, Australia

*** Correspondence:**Dr. Mariona Jové, Departament de Medicina Experimental, Universitat de Lleida-Institut de Recerca Biomedica de Lleida (IRBLleida), Edifici Biomedicina 1, Av. Alcalde Rovira Roure-80, Lleida 25198, Catalonia, Spain. Phone: (+34)973702442

[mariona.jove@udl.cat](mailto:mariona.jove@udl.cat)

Prof. Dr. Reinald Pamplona, Departament de Medicina Experimental, Universitat de Lleida-Institut de Recerca Biomedica de Lleida (IRBLleida), Edifici Biomedicina 1, Av. Alcalde Rovira Roure-80, Lleida 25198, Catalonia, Spain. Phone: (+34)973702442

[reinald.pamplona@mex.udl.cat](mailto:reinald.pamplona@mex.udl.cat)

# Supplementary Tables

**Table S2.** Conditions for tandem mass spectrometry quantification of all the lipid species detected by targeted lipidomic analysis

| Lipid Name | Internal Standard | Transition | RT | TS | CE | CAV |
| --- | --- | --- | --- | --- | --- | --- |
| AcylCarnitine 12:0 | LPC 13:0 | 344.3 -> 85.1 | 1,28 | 1 | 30 | 5 |
| AcylCarnitine 13:0 | LPC 13:0 | 358.3 -> 85.1 | 1,459 | 1 | 30 | 5 |
| AcylCarnitine 14:0 | LPC 13:0 | 372.3 -> 85.1 | 1,871 | 1 | 30 | 5 |
| AcylCarnitine 14:1 | LPC 13:0 | 370.3 -> 85.1 | 1,489 | 1 | 30 | 5 |
| AcylCarnitine 14:2 | LPC 13:0 | 368.3 -> 85.1 | 1,235 | 1 | 30 | 5 |
| AcylCarnitine 15:0(a) | LPC 13:0 | 386.3 -> 85.1 | 1,94 | 1 | 30 | 5 |
| AcylCarnitine 15:0(b) | LPC 13:0 | 386.3 -> 85.1 | 2,12 | 1 | 30 | 5 |
| AcylCarnitine 16:0 | LPC 13:0 | 400.4 -> 85.1 | 2,562 | 1 | 30 | 5 |
| AcylCarnitine 16:1 | LPC 13:0 | 398.3 -> 85.1 | 2,054 | 1 | 30 | 5 |
| AcylCarnitine 17:0(a) | LPC 13:0 | 414.4 -> 85.1 | 2,64 | 1 | 30 | 5 |
| AcylCarnitine 17:0(b) | LPC 13:0 | 414.4 -> 85.1 | 2,78 | 1 | 30 | 5 |
| AcylCarnitine 18:0 | LPC 13:0 | 428.4 -> 85.1 | 3,224 | 1 | 30 | 5 |
| AcylCarnitine 18:1 | LPC 13:0 | 426.4 -> 85.1 | 2,702 | 1 | 30 | 5 |
| AcylCarnitine 18:2 | LPC 13:0 | 424.3 -> 85.1 | 2,291 | 1 | 30 | 5 |
| CE 14:0 | CE 18:0-d6 | 614.6 -> 369.3 | 11,447 | 1 | 10 | 5 |
| CE 15:0 | CE 18:0-d6 | 628.6 -> 369.3 | 11,678 | 1 | 10 | 5 |
| CE 16:0 | CE 18:0-d6 | 642.6 -> 369.3 | 11,76 | 1 | 10 | 5 |
| CE 16:1 | CE 18:0-d6 | 640.6 -> 369.3 | 11,52 | 1 | 10 | 5 |
| CE 16:2 | CE 18:0-d6 | 638.6 -> 369.3 | 11,373 | 1 | 10 | 5 |
| CE 17:0 | CE 18:0-d6 | 656.6 -> 369.3 | 11,873 | 1 | 10 | 5 |
| CE 17:1 | CE 18:0-d6 | 654.6 -> 369.3 | 11,656 | 1 | 10 | 5 |
| CE 18:0 | CE 18:0-d6 | 670.7 -> 369.3 | 12,016 | 1 | 10 | 5 |
| CE 18:1 | CE 18:0-d6 | 668.6 -> 369.3 | 11,831 | 1 | 10 | 5 |
| CE 18:2 | CE 18:0-d6 | 666.6 -> 369.3 | 11,561 | 1 | 10 | 5 |
| CE 18:3 | CE 18:0-d6 | 664.6 -> 369.3 | 11,382 | 1 | 10 | 5 |
| CE 20:1 | CE 18:0-d6 | 696.7 -> 369.3 | 12,014 | 1 | 10 | 5 |
| CE 20:2 | CE 18:0-d6 | 694.7 -> 369.3 | 11,84 | 1 | 10 | 5 |
| CE 20:3 | CE 18:0-d6 | 692.6 -> 369.3 | 11,611 | 1 | 10 | 5 |
| CE 20:4 | CE 18:0-d6 | 690.6 -> 369.3 | 11,444 | 1 | 10 | 5 |
| CE 20:5 | CE 18:0-d6 | 688.6 -> 369.3 | 11,203 | 1 | 10 | 5 |
| CE 22:0 | CE 18:0-d6 | 726.7 -> 369.3 | 12,336 | 1 | 10 | 5 |
| CE 22:1 | CE 18:0-d6 | 724.7 -> 369.3 | 12,165 | 1 | 10 | 5 |
| CE 22:4 | CE 18:0-d6 | 718.7 -> 369.3 | 11,674 | 1 | 10 | 5 |
| CE 22:5(a) | CE 18:0-d6 | 716.6 -> 369.3 | 11,443 | 1 | 10 | 5 |
| CE 22:5(b) | CE 18:0-d6 | 716.6 -> 369.3 | 11,57 | 1 | 10 | 5 |
| CE 22:6 | CE 18:0-d6 | 714.6 -> 369.3 | 11,317 | 1 | 10 | 5 |
| CE 24:0 | CE 18:0-d6 | 754.7 -> 369.3 | 12,432 | 1 | 10 | 5 |
| CE 24:1 | CE 18:0-d6 | 752.7 -> 369.3 | 12,324 | 1 | 10 | 5 |
| CE 24:4 | CE 18:0-d6 | 746.7 -> 369.3 | 11,91 | 1 | 10 | 5 |
| CE 24:5 | CE 18:0-d6 | 744.7 -> 369.3 | 11,714 | 1 | 10 | 5 |
| CE 24:6 | CE 18:0-d6 | 742.7 -> 369.3 | 11,527 | 1 | 10 | 5 |
| Cer(d16:1/16:0) | Cer 17:0 | 510.6 -> 236.3 | 6,69 | 1 | 29 | 5 |
| Cer(d16:1/18:0) | Cer 17:0 | 538.6 -> 236.3 | 7,832 | 1 | 29 | 5 |
| Cer(d16:1/20:0) | Cer 17:0 | 566.6 -> 236.3 | 9,099 | 1 | 29 | 5 |
| Cer(d16:1/22:0) | Cer 17:0 | 594.6 -> 236.3 | 10,045 | 1 | 29 | 5 |
| Cer(d16:1/23:0) | Cer 17:0 | 608.6 -> 236.3 | 10,112 | 1 | 29 | 5 |
| Cer(d16:1/24:0) | Cer 17:0 | 622.6 -> 236.3 | 10,25 | 1 | 29 | 5 |
| Cer(d16:1/24:1) | Cer 17:0 | 620.6 -> 236.3 | 10,043 | 1 | 29 | 5 |
| Cer(d17:1/16:0) | Cer 17:0 | 524.6 -> 250.3 | 7,173 | 1 | 29 | 5 |
| Cer(d17:1/18:0) | Cer 17:0 | 552.6 -> 250.3 | 8,44 | 1 | 29 | 5 |
| Cer(d17:1/20:0) | Cer 17:0 | 580.6 -> 250.3 | 9,74 | 1 | 29 | 5 |
| Cer(d17:1/22:0) | Cer 17:0 | 608.6 -> 250.3 | 10,135 | 1 | 29 | 5 |
| Cer(d17:1/23:0) | Cer 17:0 | 622.6 -> 250.3 | 10,226 | 1 | 29 | 5 |
| Cer(d17:1/24:0) | Cer 17:0 | 636.6 -> 250.3 | 10,295 | 1 | 29 | 5 |
| Cer(d17:1/24:1) | Cer 17:0 | 634.6 -> 250.3 | 10,144 | 1 | 29 | 5 |
| Cer(d18:1/14:0) | Cer 17:0 | 510.5 -> 264.3 | 6,702 | 1 | 29 | 5 |
| Cer(d18:1/16:0) | Cer 17:0 | 538.5 -> 264.3 | 7,785 | 1 | 29 | 5 |
| Cer(d18:1/18:0) | Cer 17:0 | 566.6 -> 264.3 | 9,056 | 1 | 29 | 5 |
| Cer(d18:1/19:0) | Cer 17:0 | 580.6 -> 264.3 | 9,707 | 1 | 29 | 5 |
| Cer(d18:1/20:0) | Cer 17:0 | 594.6 -> 264.3 | 10,022 | 1 | 29 | 5 |
| Cer(d18:1/21:0) | Cer 17:0 | 608.6 -> 264.3 | 10,124 | 1 | 29 | 5 |
| Cer(d18:1/22:0) | Cer 17:0 | 622.6 -> 264.3 | 10,226 | 1 | 29 | 5 |
| Cer(d18:1/23:0) | Cer 17:0 | 636.6 -> 264.3 | 10,318 | 1 | 29 | 5 |
| Cer(d18:1/24:0) | Cer 17:0 | 650.6 -> 264.3 | 10,42 | 1 | 29 | 5 |
| Cer(d18:1/24:1) | Cer 17:0 | 648.6 -> 264.3 | 10,224 | 1 | 29 | 5 |
| Cer(d18:1/26:0) | Cer 17:0 | 678.6 -> 264.3 | 10,574 | 1 | 29 | 5 |
| Cer(d18:1x/18:0) | dhCer 8:0 | 566.6 -> 282.3 | 9,73 | 1 | 31 | 4 |
| Cer(d18:1x/20:0) | dhCer 8:0 | 594.6 -> 282.3 | 10,299 | 1 | 31 | 4 |
| Cer(d18:1x/22:0) | dhCer 8:0 | 622.6 -> 282.3 | 10,48 | 1 | 31 | 4 |
| Cer(d18:1x/24:0) | dhCer 8:0 | 650.7 -> 282.3 | 10,644 | 1 | 31 | 4 |
| Cer(d18:1x/24:1) | dhCer 8:0 | 648.6 -> 282.3 | 10,501 | 1 | 31 | 4 |
| Cer(d18:2/14:0) | Cer 17:0 | 508.5 -> 262.3 | 5,9 | 1 | 23 | 5 |
| Cer(d18:2/16:0) | Cer 17:0 | 536.5 -> 262.3 | 6,951 | 1 | 23 | 5 |
| Cer(d18:2/17:0) | Cer 17:0 | 550.5 -> 262.3 | 7,494 | 1 | 23 | 5 |
| Cer(d18:2/18:0) | Cer 17:0 | 564.6 -> 262.3 | 8,105 | 1 | 23 | 5 |
| Cer(d18:2/20:0) | Cer 17:0 | 592.6 -> 262.3 | 9,347 | 1 | 23 | 5 |
| Cer(d18:2/21:0) | Cer 17:0 | 606.6 -> 262.3 | 9,929 | 1 | 29 | 5 |
| Cer(d18:2/22:0) | Cer 17:0 | 620.6 -> 262.3 | 10,054 | 1 | 23 | 5 |
| Cer(d18:2/23:0) | Cer 17:0 | 634.6 -> 262.3 | 10,214 | 1 | 29 | 5 |
| Cer(d18:2/24:0) | Cer 17:0 | 648.6 -> 262.3 | 10,259 | 1 | 23 | 5 |
| Cer(d18:2/24:1) | Cer 17:0 | 646.6 -> 262.3 | 10,063 | 1 | 23 | 5 |
| Cer(d18:2/26:0) | Cer 17:0 | 676.6 -> 262.3 | 10,395 | 1 | 23 | 5 |
| Cer(d19:1/16:0) | Cer 17:0 | 552.6 -> 278.3 | 8,195 | 1 | 29 | 5 |
| Cer(d19:1/18:0) | Cer 17:0 | 580.6 -> 278.3 | 9,348 | 1 | 29 | 5 |
| Cer(d19:1/20:0) | Cer 17:0 | 608.6 -> 278.3 | 10,09 | 1 | 29 | 5 |
| Cer(d19:1/22:0) | Cer 17:0 | 636.6 -> 278.3 | 10,272 | 1 | 29 | 5 |
| Cer(d19:1/23:0) | Cer 17:0 | 650.6 -> 278.3 | 10,351 | 1 | 29 | 5 |
| Cer(d19:1/24:0) | Cer 17:0 | 664.6 -> 278.3 | 10,407 | 1 | 29 | 5 |
| Cer(d19:1/24:1) | Cer 17:0 | 662.6 -> 278.3 | 10,28 | 1 | 29 | 5 |
| Cer(d19:1/26:0) | Cer 17:0 | 692.6 -> 278.3 | 10,595 | 1 | 29 | 5 |
| Cer(d20:1/22:0) | Cer 17:0 | 650.6 -> 292.3 | 10,374 | 1 | 29 | 5 |
| Cer(d20:1/23:0) | Cer 17:0 | 664.6 -> 292.3 | 10,441 | 1 | 29 | 5 |
| Cer(d20:1/24:0) | Cer 17:0 | 678.6 -> 292.3 | 10,519 | 1 | 29 | 5 |
| Cer(d20:1/24:1) | Cer 17:0 | 676.6 -> 292.3 | 10,372 | 1 | 29 | 5 |
| Cer(d20:1/26:0) | Cer 17:0 | 706.6 -> 292.3 | 10,737 | 1 | 29 | 5 |
| Cer1P(d18:1/16:0) | Cer 17:0 | 618.4 -> 264.3 | 5,521 | 1 | 29 | 4 |
| COH (161) | COH-d7 (161) | 369.4 -> 161.2 | 6,351 | 1 | 23 | 5 |
| Desmosterol 16:0 | CE 18:0-d6 | 640.8 -> 367.4 | 11,541 | 1 | 12 | 4 |
| Desmosterol 18:1 | CE 18:0-d6 | 666.8 -> 367.4 | 11,634 | 1 | 12 | 4 |
| Desmosterol 18:2 | CE 18:0-d6 | 664.8 -> 367.4 | 11,267 | 1 | 12 | 4 |
| Desmosterol 20:4 | CE 18:0-d6 | 688.8 -> 367.4 | 11,16 | 1 | 12 | 4 |
| Desmosterol 20:5 | CE 18:0-d6 | 686.8 -> 367.4 | 10,973 | 1 | 12 | 4 |
| Desmosterol 22:6 | CE 18:0-d6 | 712.8 -> 367.4 | 11,086 | 1 | 12 | 4 |
| DG 30:0 -(14:0) | DG 15:0 15:0 | 558.5 -> 313.3 | 8,751 | 1 | 21 | 5 |
| DG 32:0 -(16:0) | DG 15:0 15:0 | 586.5 -> 313.2 | 9,919 | 1 | 21 | 5 |
| DG 32:1 -(16:1) | DG 15:0 15:0 | 584.5 -> 313.2 | 8,958 | 1 | 21 | 5 |
| DG 32:2 -(18:2) | DG 15:0 15:0 | 582.5 -> 285.2 | 8,093 | 1 | 21 | 5 |
| DG 34:1 -(18:1) | DG 15:0 15:0 | 612.6 -> 313.3 | 10,02 | 1 | 21 | 5 |
| DG 34:2 -(16:1) | DG 15:0 15:0 | 610.5 -> 339.2 | 9,152 | 1 | 21 | 5 |
| DG 34:2 -(18:2) | DG 15:0 15:0 | 610.5 -> 313.2 | 9,314 | 1 | 21 | 5 |
| DG 36:1 -(18:1) | DG 15:0 15:0 | 640.6 -> 341.3 | 10,132 | 1 | 21 | 5 |
| DG 36:2 -(18:1) | DG 15:0 15:0 | 638.6 -> 339.3 | 10,063 | 1 | 21 | 5 |
| DG 36:2 -(18:2) | DG 15:0 15:0 | 638.6 -> 341.3 | 10,086 | 1 | 21 | 5 |
| DG 36:3 -(18:2) | DG 15:0 15:0 | 636.6 -> 339.3 | 9,53 | 1 | 21 | 5 |
| DG 36:4 -(18:2) | DG 15:0 15:0 | 634.5 -> 337.2 | 8,662 | 1 | 21 | 5 |
| DG 36:4 -(18:3) | DG 15:0 15:0 | 634.5 -> 339.2 | 8,88 | 1 | 21 | 5 |
| DG 36:4 -(20:4) | DG 15:0 15:0 | 634.5 -> 313.2 | 9,151 | 1 | 21 | 5 |
| DG 38:4 -(20:3) | DG 15:0 15:0 | 662.6 -> 339.3 | 9,879 | 1 | 21 | 5 |
| DG 38:4 -(20:4) | DG 15:0 15:0 | 662.6 -> 341.3 | 10,05 | 1 | 21 | 5 |
| DG 38:5 -(20:4) | DG 15:0 15:0 | 660.6 -> 339.3 | 9,333 | 1 | 21 | 5 |
| DG 38:5 -(22:5) | DG 15:0 15:0 | 660.6 -> 313.3 | 9,54 | 1 | 21 | 5 |
| DG 38:6 -(20:4) | DG 15:0 15:0 | 658.5 -> 337.2 | 8,517 | 1 | 21 | 5 |
| DG 38:6 -(22:6) | DG 15:0 15:0 | 658.5 -> 313.2 | 8,967 | 1 | 21 | 5 |
| dhCer 16:0 | dhCer 8:0 | 540.5 -> 284.3 | 8,3 | 1 | 30 | 5 |
| dhCer 18:0 | dhCer 8:0 | 568.6 -> 284.3 | 9,6 | 1 | 30 | 5 |
| dhCer 20:0 | dhCer 8:0 | 596.6 -> 284.3 | 10,033 | 1 | 30 | 5 |
| dhCer 22:0 | dhCer 8:0 | 624.6 -> 284.3 | 10,284 | 1 | 30 | 5 |
| dhCer 24:0 | dhCer 8:0 | 652.7 -> 284.3 | 10,453 | 1 | 30 | 5 |
| dhCer 24:1 | dhCer 8:0 | 650.6 -> 284.3 | 10,281 | 1 | 30 | 5 |
| GM1(d18:1/16:0) | Hex3Cer(d18:1/17:0) | 760.1 -> 366.2 | 4,692 | 1 | 9 | 3 |
| GM3(d18:1/16:0) | Hex3Cer(d18:1/17:0) | 1153.7 -> 264.3 | 4,849 | 1 | 57 | 5 |
| GM3(d18:1/18:0) | Hex3Cer(d18:1/17:0) | 1181.8 -> 264.3 | 5,458 | 1 | 57 | 5 |
| GM3(d18:1/20:0) | Hex3Cer(d18:1/17:0) | 1209.8 -> 264.3 | 6,237 | 1 | 57 | 5 |
| GM3(d18:1/22:0) | Hex3Cer(d18:1/17:0) | 1237.8 -> 264.3 | 7,645 | 1 | 57 | 5 |
| GM3(d18:1/24:0) | Hex3Cer(d18:1/17:0) | 1265.8 -> 264.3 | 8,106 | 1 | 57 | 5 |
| GM3(d18:1/24:1) | Hex3Cer(d18:1/17:0) | 1263.8 -> 264.3 | 7,161 | 1 | 57 | 5 |
| GM3(d18:2/24:1) | Hex3Cer(d18:1/17:0) | 1261.8 -> 262.3 | 6,49 | 1 | 57 | 5 |
| Hex1Cer(d16:1/18:0) | Hex2Cer(d18:1/16:0)d3 | 700.6 -> 236.3 | 6,419 | 1 | 33 | 5 |
| Hex1Cer(d16:1/20:0) | Hex2Cer(d18:1/16:0)d3 | 728.6 -> 236.3 | 7,412 | 1 | 33 | 5 |
| Hex1Cer(d16:1/22:0) | Hex2Cer(d18:1/16:0)d3 | 756.7 -> 236.3 | 8,802 | 1 | 33 | 5 |
| Hex1Cer(d16:1/24:0) | Hex2Cer(d18:1/16:0)d3 | 784.7 -> 236.3 | 9,878 | 1 | 33 | 5 |
| Hex1Cer(d18:1/16:0) | Hex2Cer(d18:1/16:0)d3 | 700.6 -> 264.3 | 6,396 | 1 | 33 | 5 |
| Hex1Cer(d18:1/18:0) | Hex2Cer(d18:1/16:0)d3 | 728.6 -> 264.3 | 7,515 | 1 | 33 | 5 |
| Hex1Cer(d18:1/20:0) | Hex2Cer(d18:1/16:0)d3 | 756.6 -> 264.3 | 8,56 | 1 | 33 | 5 |
| Hex1Cer(d18:1/22:0) | Hex2Cer(d18:1/16:0)d3 | 784.7 -> 264.3 | 9,923 | 1 | 33 | 5 |
| Hex1Cer(d18:1/24:0) | Hex2Cer(d18:1/16:0)d3 | 812.7 -> 264.3 | 10,139 | 1 | 33 | 5 |
| Hex1Cer(d18:1/24:1) | Hex2Cer(d18:1/16:0)d3 | 810.7 -> 264.3 | 9,945 | 1 | 33 | 5 |
| Hex1Cer(d18:2/18:0) | Hex2Cer(d18:1/16:0)d3 | 726.6 -> 262.3 | 6,712 | 1 | 33 | 5 |
| Hex1Cer(d18:2/20:0) | Hex2Cer(d18:1/16:0)d3 | 754.6 -> 262.3 | 7,651 | 1 | 33 | 5 |
| Hex1Cer(d18:2/22:0) | Hex2Cer(d18:1/16:0)d3 | 782.7 -> 262.3 | 8,964 | 1 | 33 | 5 |
| Hex1Cer(d18:2/24:0) | Hex2Cer(d18:1/16:0)d3 | 810.7 -> 262.3 | 9,956 | 1 | 33 | 5 |
| Hex2Cer(d16:1/16:0) | Hex2Cer(d18:1/16:0)d3 | 834.6 -> 236.3 | 5,161 | 1 | 53 | 5 |
| Hex2Cer(d16:1/24:1) | Hex2Cer(d18:1/16:0)d3 | 944.7 -> 236.3 | 7,993 | 1 | 53 | 5 |
| Hex2Cer(d18:1/16:0) | Hex2Cer(d18:1/16:0)d3 | 862.6 -> 264.3 | 5,867 | 1 | 53 | 5 |
| Hex2Cer(d18:1/18:0) | Hex2Cer(d18:1/16:0)d3 | 890.7 -> 264.3 | 7,553 | 1 | 53 | 5 |
| Hex2Cer(d18:1/20:0) | Hex2Cer(d18:1/16:0)d3 | 918.7 -> 264.3 | 7,879 | 1 | 53 | 5 |
| Hex2Cer(d18:1/22:0) | Hex2Cer(d18:1/16:0)d3 | 946.7 -> 264.3 | 9,08 | 1 | 53 | 5 |
| Hex2Cer(d18:1/24:0) | Hex2Cer(d18:1/16:0)d3 | 974.8 -> 264.3 | 9,989 | 1 | 53 | 5 |
| Hex2Cer(d18:1/24:1) | Hex2Cer(d18:1/16:0)d3 | 972.7 -> 264.3 | 9,111 | 1 | 53 | 5 |
| Hex2Cer(d18:2/16:0) | Hex2Cer(d18:1/16:0)d3 | 860.6 -> 262.3 | 5,321 | 1 | 53 | 5 |
| Hex2Cer(d18:2/24:1) | Hex2Cer(d18:1/16:0)d3 | 970.7 -> 262.3 | 8,229 | 1 | 53 | 5 |
| Hex3Cer(d18:1/16:0) | Hex3Cer(d18:1/17:0) | 1024.7 -> 264.3 | 5,588 | 1 | 57 | 5 |
| Hex3Cer(d18:1/18:0) | Hex3Cer(d18:1/17:0) | 1052.7 -> 264.3 | 6,421 | 1 | 57 | 5 |
| Hex3Cer(d18:1/20:0) | Hex3Cer(d18:1/17:0) | 1080.7 -> 264.3 | 7,438 | 1 | 57 | 5 |
| Hex3Cer(d18:1/22:0) | Hex3Cer(d18:1/17:0) | 1108.8 -> 264.3 | 8,576 | 1 | 57 | 5 |
| Hex3Cer(d18:1/24:0) | Hex3Cer(d18:1/17:0) | 1136.8 -> 264.3 | 9,785 | 1 | 57 | 5 |
| Hex3Cer(d18:1/24:1) | Hex3Cer(d18:1/17:0) | 1134.8 -> 264.3 | 8,62 | 1 | 57 | 5 |
| LPC 14:0(a) | LPC 13:0 | 468.3 -> 184.1 | 1,805 | 1 | 21 | 5 |
| LPC 14:0(b) | LPC 13:0 | 468.3 -> 184.1 | 2,009 | 1 | 21 | 5 |
| LPC 15:0(a) | LPC 13:0 | 482.3 -> 184.1 | 2,171 | 1 | 21 | 5 |
| LPC 15:0(b) | LPC 13:0 | 482.3 -> 184.1 | 2,355 | 1 | 21 | 5 |
| LPC 16:0(a) | LPC 13:0 | 496.3 -> 184.1 | 2,495 | 1 | 21 | 5 |
| LPC 16:0(b) | LPC 13:0 | 496.3 -> 184.1 | 2,71 | 1 | 21 | 5 |
| LPC 16:1(a) | LPC 13:0 | 494.3 -> 184.1 | 1,997 | 1 | 21 | 5 |
| LPC 16:1(b) | LPC 13:0 | 494.3 -> 184.1 | 2,235 | 1 | 21 | 5 |
| LPC 17:0(a) | LPC 13:0 | 510.4 -> 184.1 | 2,72 | 1 | 21 | 5 |
| LPC 17:0(b) | LPC 13:0 | 510.4 -> 184.1 | 2,895 | 1 | 21 | 5 |
| LPC 17:0(b) [104_sn1] | LPC 13:0 | 510.4 -> 104.1 | 2,862 | 1 | 21 | 5 |
| LPC 17:0(c) | LPC 13:0 | 510.4 -> 184.1 | 3,036 | 1 | 21 | 5 |
| LPC 17:1(a) | LPC 13:0 | 508.4 -> 184.1 | 2,343 | 1 | 21 | 5 |
| LPC 17:1(b) | LPC 13:0 | 508.4 -> 184.1 | 2,526 | 1 | 21 | 5 |
| LPC 17:1(b) [104_sn1] | LPC 13:0 | 508.4 -> 104.1 | 2,515 | 1 | 21 | 5 |
| LPC 17:1(c) | LPC 13:0 | 508.4 -> 184.1 | 2,731 | 1 | 21 | 5 |
| LPC 18:0(a) | LPC 13:0 | 524.4 -> 184.1 | 3,199 | 1 | 21 | 5 |
| LPC 18:0(b) | LPC 13:0 | 524.4 -> 184.1 | 3,38 | 1 | 21 | 5 |
| LPC 18:1(a) | LPC 13:0 | 522.4 -> 184.1 | 2,676 | 1 | 21 | 5 |
| LPC 18:1(b) | LPC 13:0 | 522.4 -> 184.1 | 2,873 | 1 | 21 | 5 |
| LPC 18:2(a) | LPC 13:0 | 520.3 -> 184.1 | 2,211 | 1 | 21 | 5 |
| LPC 18:2(b) | LPC 13:0 | 520.3 -> 184.1 | 2,417 | 1 | 21 | 5 |
| LPC 18:3(a) | LPC 13:0 | 518.3 -> 184.1 | 1,835 | 1 | 21 | 5 |
| LPC 18:3(b) | LPC 13:0 | 518.3 -> 184.1 | 1,952 | 1 | 21 | 5 |
| LPC 18:3(b) [104_sn1] | LPC 13:0 | 518.3 -> 104.1 | 1,931 | 1 | 21 | 5 |
| LPC 18:3(c) | LPC 13:0 | 518.3 -> 184.1 | 2,115 | 1 | 21 | 5 |
| LPC 19:0(a) | LPC 13:0 | 538.4 -> 184.1 | 3,389 | 1 | 21 | 5 |
| LPC 19:0(b) | LPC 13:0 | 538.4 -> 184.1 | 3,526 | 1 | 21 | 5 |
| LPC 19:0(b) [104_sn1] | LPC 13:0 | 538.4 -> 104.1 | 3,505 | 1 | 21 | 5 |
| LPC 19:0(c) | LPC 13:0 | 538.4 -> 184.1 | 3,695 | 1 | 21 | 5 |
| LPC 19:1(a) | LPC 13:0 | 536.4 -> 184.1 | 2,98 | 1 | 21 | 5 |
| LPC 19:1(b) | LPC 13:0 | 536.4 -> 184.1 | 3,198 | 1 | 21 | 5 |
| LPC 19:1(c) | LPC 13:0 | 536.4 -> 184.1 | 3,4 | 1 | 21 | 5 |
| LPC 20:0(a) | LPC 13:0 | 552.4 -> 184.1 | 3,758 | 1 | 21 | 5 |
| LPC 20:0(b) | LPC 13:0 | 552.4 -> 184.1 | 3,929 | 1 | 21 | 5 |
| LPC 20:1(a) | LPC 13:0 | 550.4 -> 184.1 | 3,303 | 1 | 21 | 5 |
| LPC 20:1(b) | LPC 13:0 | 550.4 -> 184.1 | 3,515 | 1 | 21 | 5 |
| LPC 20:2(a) | LPC 13:0 | 548.4 -> 184.1 | 2,87 | 1 | 21 | 5 |
| LPC 20:2(b) | LPC 13:0 | 548.4 -> 184.1 | 3,077 | 1 | 21 | 5 |
| LPC 20:3(a) | LPC 13:0 | 546.4 -> 184.1 | 2,491 | 1 | 21 | 5 |
| LPC 20:3(c) | LPC 13:0 | 546.4 -> 184.1 | 2,641 | 1 | 21 | 5 |
| LPC 20:4(a) | LPC 13:0 | 544.3 -> 184.1 | 2,21 | 1 | 21 | 5 |
| LPC 20:4(b) | LPC 13:0 | 544.3 -> 184.1 | 2,383 | 1 | 21 | 5 |
| LPC 20:5(a) | LPC 13:0 | 542.3 -> 184.1 | 1,823 | 1 | 21 | 5 |
| LPC 20:5(b) | LPC 13:0 | 542.3 -> 184.1 | 1,994 | 1 | 21 | 5 |
| LPC 22:0(a) | LPC 13:0 | 580.4 -> 184.1 | 4,272 | 1 | 21 | 5 |
| LPC 22:0(b) | LPC 13:0 | 580.4 -> 184.1 | 4,463 | 1 | 21 | 5 |
| LPC 22:1(a) | LPC 13:0 | 578.4 -> 184.1 | 3,821 | 1 | 21 | 5 |
| LPC 22:1(b) | LPC 13:0 | 578.4 -> 184.1 | 4,001 | 1 | 21 | 5 |
| LPC 22:4(a) | LPC 13:0 | 572.4 -> 184.1 | 2,76 | 1 | 21 | 5 |
| LPC 22:4(b) | LPC 13:0 | 572.4 -> 184.1 | 2,902 | 1 | 21 | 5 |
| LPC 22:5(a) | LPC 13:0 | 570.4 -> 184.1 | 2,371 | 1 | 21 | 5 |
| LPC 22:5(b) | LPC 13:0 | 570.4 -> 184.1 | 2,522 | 1 | 21 | 5 |
| LPC 22:5(b) [104_sn1] | LPC 13:0 | 570.4 -> 104.1 | 2,501 | 1 | 21 | 5 |
| LPC 22:5(c) | LPC 13:0 | 570.4 -> 184.1 | 2,706 | 1 | 21 | 5 |
| LPC 22:6(a) | LPC 13:0 | 568.3 -> 184.1 | 2,124 | 1 | 21 | 5 |
| LPC 22:6(b) | LPC 13:0 | 568.3 -> 184.1 | 2,307 | 1 | 21 | 5 |
| LPC 24:0(a) | LPC 13:0 | 608.5 -> 184.1 | 4,887 | 1 | 21 | 5 |
| LPC 24:0(b) | LPC 13:0 | 608.5 -> 184.1 | 5,123 | 1 | 21 | 5 |
| LPC 26:0(a) | LPC 13:0 | 636.5 -> 184.1 | 5,663 | 1 | 21 | 5 |
| LPC 26:0(b) | LPC 13:0 | 636.5 -> 184.1 | 5,942 | 1 | 21 | 5 |
| LPC(P-16:0) | LPC 13:0 | 480.3 -> 104.1 | 2,951 | 1 | 21 | 5 |
| LPC(P-17:0)(a) | LPC 13:0 | 494.3 -> 104.1 | 3,135 | 1 | 21 | 5 |
| LPC(P-17:0)(b) | LPC 13:0 | 494.3 -> 104.1 | 3,307 | 1 | 21 | 5 |
| LPC(P-18:0) | LPC 13:0 | 508.3 -> 104.1 | 3,602 | 1 | 21 | 5 |
| LPC(P-18:1) | LPC 13:0 | 506.3 -> 104.1 | 3,146 | 1 | 21 | 5 |
| LPC(P-20:0) | LPC 13:0 | 536.3 -> 104.1 | 4,099 | 1 | 21 | 5 |
| LPE 16:0(a) | LPE 14:0 | 454.3 -> 313.3 | 2,604 | 1 | 17 | 5 |
| LPE 16:0(b) | LPE 14:0 | 454.3 -> 313.3 | 2,822 | 1 | 17 | 5 |
| LPE 17:0(a) | LPE 14:0 | 468.3 -> 327.3 | 2,984 | 1 | 17 | 5 |
| LPE 17:0(b) | LPE 14:0 | 468.3 -> 327.3 | 3,148 | 1 | 17 | 5 |
| LPE 18:0(a) | LPE 14:0 | 482.3 -> 341.3 | 3,307 | 1 | 17 | 5 |
| LPE 18:0(b) | LPE 14:0 | 482.3 -> 341.3 | 3,487 | 1 | 17 | 5 |
| LPE 18:1(a) | LPE 14:0 | 480.3 -> 339.3 | 2,755 | 1 | 17 | 5 |
| LPE 18:1(b) | LPE 14:0 | 480.3 -> 339.3 | 2,973 | 1 | 17 | 5 |
| LPE 18:2(a) | LPE 14:0 | 478.3 -> 337.3 | 2,301 | 1 | 17 | 5 |
| LPE 18:2(b) | LPE 14:0 | 478.3 -> 337.3 | 2,517 | 1 | 17 | 5 |
| LPE 20:4(a) | LPE 14:0 | 502.3 -> 361.3 | 2,332 | 1 | 17 | 5 |
| LPE 20:4(b) | LPE 14:0 | 502.3 -> 361.3 | 2,472 | 1 | 17 | 5 |
| LPE 22:6(a) | LPE 14:0 | 526.3 -> 385.3 | 2,265 | 1 | 17 | 5 |
| LPE 22:6(b) | LPE 14:0 | 526.3 -> 385.3 | 2,405 | 1 | 17 | 5 |
| LPE(P-16:0) | LPE 14:0 | 438.3 -> 266.4 | 3,083 | 1 | 19 | 5 |
| LPE(P-18:0) | LPE 14:0 | 466.3 -> 294.4 | 3,71 | 1 | 19 | 5 |
| LPE(P-18:1) | LPE 14:0 | 464.3 -> 292.4 | 3,213 | 1 | 19 | 5 |
| LPE(P-20:0) | LPE 14:0 | 494.3 -> 322.4 | 4,226 | 1 | 19 | 5 |
| LPI 18:0(a) | LPE 14:0 | 618.3 -> 341.3 | 2,727 | 1 | 17 | 5 |
| LPI 18:0(b) | LPE 14:0 | 618.3 -> 341.3 | 2,88 | 1 | 17 | 5 |
| LPI 18:1(a) | LPE 14:0 | 616.3 -> 339.3 | 2,219 | 1 | 17 | 5 |
| LPI 18:1(b) | LPE 14:0 | 616.3 -> 339.3 | 2,338 | 1 | 17 | 5 |
| LPI 18:2(a) | LPE 14:0 | 614.3 -> 337.3 | 1,822 | 1 | 17 | 5 |
| LPI 18:2(b) | LPE 14:0 | 614.3 -> 337.3 | 1,971 | 1 | 17 | 5 |
| LPI 20:4(a) | LPE 14:0 | 638.3 -> 361.3 | 1,768 | 1 | 17 | 5 |
| LPI 20:4(b) | LPE 14:0 | 638.3 -> 361.3 | 1,971 | 1 | 17 | 5 |
| LPS(16:0) | PS 17:0 17:0 | 498.5 -> 313.5 | 2,645 | 2 | 20 | 5 |
| LPS(18:0) | PS 17:0 17:0 | 526.5 -> 341.5 | 3,326 | 2 | 20 | 5 |
| LPS(18:1) | PS 17:0 17:0 | 524.5 -> 339.5 | 2,806 | 2 | 20 | 5 |
| LPS(18:2) | PS 17:0 17:0 | 522.5 -> 337.5 | 2,352 | 2 | 20 | 5 |
| OH-Cholesterol 18:0 | CE 18:0-d6 | 686.8 -> 367.4 | 11,151 | 2 | 12 | 4 |
| OH-Cholesterol 18:1 (a) | CE 18:0-d6 | 684.8 -> 367.4 | 10,716 | 2 | 12 | 4 |
| OH-Cholesterol 18:1 (b) | CE 18:0-d6 | 684.8 -> 367.4 | 10,963 | 2 | 12 | 4 |
| OH-Cholesterol 18:2 (a) | CE 18:0-d6 | 682.8 -> 367.4 | 10,267 | 2 | 12 | 4 |
| OH-Cholesterol 18:2 (b) | CE 18:0-d6 | 682.8 -> 367.4 | 10,428 | 2 | 12 | 4 |
| OH-Cholesterol 18:2 (c) | CE 18:0-d6 | 682.8 -> 367.4 | 10,596 | 2 | 12 | 4 |
| OH-Cholesterol 18:2 (d) | CE 18:0-d6 | 682.8 -> 367.4 | 10,781 | 2 | 12 | 4 |
| OH-Cholesterol 18:2 (e) | CE 18:0-d6 | 682.8 -> 367.4 | 11,099 | 2 | 12 | 4 |
| OH-Cholesterol 20:4 (a) | CE 18:0-d6 | 706.8 -> 367.4 | 10,506 | 2 | 12 | 4 |
| OH-Cholesterol 20:4 (b) | CE 18:0-d6 | 706.8 -> 367.4 | 10,736 | 2 | 12 | 4 |
| oxCE 18:2 +2O NH4 | CE 18:0-d6 | 698.6 -> 369.4 | 10,405 | 1 | 10 | 5 |
| oxCE 18:2 +O NH4 | CE 18:0-d6 | 682.6 -> 369.4 | 10,585 | 1 | 10 | 5 |
| PC 28:0 | PC 13:0 13:0 | 678.5 -> 184.1 | 5,218 | 1 | 21 | 5 |
| PC 30:0 | PC 13:0 13:0 | 706.5 -> 184.1 | 6,009 | 1 | 21 | 5 |
| PC 31:0(a) | PC 13:0 13:0 | 720.6 -> 184.1 | 6,337 | 1 | 21 | 5 |
| PC 31:0(b) | PC 13:0 13:0 | 720.6 -> 184.1 | 6,337 | 1 | 21 | 5 |
| PC 32:0 | PC 13:0 13:0 | 734.6 -> 184.1 | 6,854 | 1 | 21 | 5 |
| PC 32:1 | PC 13:0 13:0 | 732.6 -> 184.1 | 6,176 | 1 | 21 | 5 |
| PC 32:2 | PC 13:0 13:0 | 730.5 -> 184.1 | 5,551 | 1 | 21 | 5 |
| PC 33:0(a) | PC 13:0 13:0 | 748.6 -> 184.1 | 7,307 | 1 | 21 | 5 |
| PC 33:0(b) | PC 13:0 13:0 | 748.6 -> 184.1 | 7,479 | 1 | 21 | 5 |
| PC 33:1 | PC 13:0 13:0 | 746.6 -> 184.1 | 6,662 | 1 | 21 | 5 |
| PC 33:2 | PC 13:0 13:0 | 744.6 -> 184.1 | 6,006 | 1 | 21 | 5 |
| PC 34:0 | PC 13:0 13:0 | 762.6 -> 184.1 | 7,918 | 1 | 21 | 5 |
| PC 34:1 | PC 13:0 13:0 | 760.6 -> 184.1 | 7,11 | 1 | 21 | 5 |
| PC 34:2 | PC 13:0 13:0 | 758.6 -> 184.1 | 6,45 | 1 | 21 | 5 |
| PC 34:3(a) | PC 13:0 13:0 | 756.6 -> 184.1 | 5,704 | 1 | 21 | 5 |
| PC 34:3(b) | PC 13:0 13:0 | 756.6 -> 184.1 | 5,871 | 1 | 21 | 5 |
| PC 34:3(c) | PC 13:0 13:0 | 756.6 -> 184.1 | 5,894 | 1 | 21 | 5 |
| PC 34:4 | PC 13:0 13:0 | 754.5 -> 184.1 | 5,54 | 1 | 21 | 5 |
| PC 34:5 | PC 13:0 13:0 | 752.5 -> 184.1 | 5,099 | 1 | 21 | 5 |
| PC 35:1(a) | PC 13:0 13:0 | 774.6 -> 184.1 | 7,373 | 1 | 21 | 5 |
| PC 35:1(b) | PC 13:0 13:0 | 774.6 -> 184.1 | 7,556 | 1 | 21 | 5 |
| PC 35:2(a) | PC 13:0 13:0 | 772.6 -> 184.1 | 6,839 | 1 | 21 | 5 |
| PC 35:2(b) | PC 13:0 13:0 | 772.6 -> 184.1 | 6,957 | 1 | 21 | 5 |
| PC 35:3(a) | PC 13:0 13:0 | 770.6 -> 184.1 | 5,96 | 1 | 21 | 5 |
| PC 35:3(b) | PC 13:0 13:0 | 770.6 -> 184.1 | 6,184 | 1 | 21 | 5 |
| PC 35:4 | PC 13:0 13:0 | 768.6 -> 184.1 | 5,96 | 1 | 21 | 5 |
| PC 35:5 | PC 13:0 13:0 | 766.5 -> 184.1 | 5,463 | 1 | 21 | 5 |
| PC 36:0 | PC 13:0 13:0 | 790.6 -> 184.1 | 9,526 | 1 | 21 | 5 |
| PC 36:1 | PC 13:0 13:0 | 788.6 -> 184.1 | 8,155 | 1 | 21 | 5 |
| PC 36:2(a) | PC 13:0 13:0 | 786.6 -> 184.1 | 7,245 | 1 | 21 | 5 |
| PC 36:2(b) | PC 13:0 13:0 | 786.6 -> 184.1 | 7,578 | 1 | 21 | 5 |
| PC 36:3(a) | PC 13:0 13:0 | 784.6 -> 184.1 | 6,39 | 1 | 21 | 5 |
| PC 36:3(b) | PC 13:0 13:0 | 784.6 -> 184.1 | 6,636 | 1 | 21 | 5 |
| PC 36:3(c) | PC 13:0 13:0 | 784.6 -> 184.1 | 7,002 | 1 | 21 | 5 |
| PC 36:4(a) | PC 13:0 13:0 | 782.6 -> 184.1 | 5,882 | 1 | 21 | 5 |
| PC 36:4(b) | PC 13:0 13:0 | 782.6 -> 184.1 | 6,39 | 1 | 21 | 5 |
| PC 36:5(a) | PC 13:0 13:0 | 780.6 -> 184.1 | 5,636 | 1 | 21 | 5 |
| PC 36:5(b) | PC 13:0 13:0 | 780.6 -> 184.1 | 5,904 | 1 | 21 | 5 |
| PC 36:6(a) | PC 13:0 13:0 | 778.5 -> 184.1 | 5,174 | 1 | 21 | 5 |
| PC 36:6(b) | PC 13:0 13:0 | 778.5 -> 184.1 | 5,409 | 1 | 21 | 5 |
| PC 37:4(a) | PC 13:0 13:0 | 796.6 -> 184.1 | 6,765 | 1 | 21 | 5 |
| PC 37:4(b) | PC 13:0 13:0 | 796.6 -> 184.1 | 6,765 | 1 | 21 | 5 |
| PC 37:6 | PC 13:0 13:0 | 792.6 -> 184.1 | 5,803 | 1 | 21 | 5 |
| PC 38:2 | PC 13:0 13:0 | 814.6 -> 184.1 | 8,454 | 1 | 21 | 5 |
| PC 38:3 | PC 13:0 13:0 | 812.6 -> 184.1 | 7,74 | 1 | 21 | 5 |
| PC 38:4(a) | PC 13:0 13:0 | 810.6 -> 184.1 | 6,966 | 1 | 21 | 5 |
| PC 38:4(b) | PC 13:0 13:0 | 810.6 -> 184.1 | 6,966 | 1 | 21 | 5 |
| PC 38:4(c) | PC 13:0 13:0 | 810.6 -> 184.1 | 7,382 | 1 | 21 | 5 |
| PC 38:5(a) | PC 13:0 13:0 | 808.6 -> 184.1 | 6,552 | 1 | 21 | 5 |
| PC 38:5(b) | PC 13:0 13:0 | 808.6 -> 184.1 | 6,813 | 1 | 21 | 5 |
| PC 38:6(a) | PC 13:0 13:0 | 806.6 -> 184.1 | 5,847 | 1 | 21 | 5 |
| PC 38:6(b) | PC 13:0 13:0 | 806.6 -> 184.1 | 6,195 | 1 | 21 | 5 |
| PC 38:7(a) | PC 13:0 13:0 | 804.6 -> 184.1 | 5,43 | 1 | 21 | 5 |
| PC 38:7(b) | PC 13:0 13:0 | 804.6 -> 184.1 | 5,571 | 1 | 21 | 5 |
| PC 38:7(c) | PC 13:0 13:0 | 804.6 -> 184.1 | 5,825 | 1 | 21 | 5 |
| PC 39:5(a) | PC 13:0 13:0 | 822.6 -> 184.1 | 6,8 | 1 | 21 | 5 |
| PC 39:5(b) | PC 13:0 13:0 | 822.6 -> 184.1 | 6,988 | 1 | 21 | 5 |
| PC 39:6(a) | PC 13:0 13:0 | 820.6 -> 184.1 | 6,469 | 1 | 21 | 5 |
| PC 39:6(b) | PC 13:0 13:0 | 820.6 -> 184.1 | 6,704 | 1 | 21 | 5 |
| PC 40:4(a) | PC 13:0 13:0 | 838.6 -> 184.1 | 8,141 | 1 | 21 | 5 |
| PC 40:4(b) | PC 13:0 13:0 | 838.6 -> 184.1 | 8,51 | 1 | 21 | 5 |
| PC 40:5(a) | PC 13:0 13:0 | 836.6 -> 184.1 | 7,473 | 1 | 21 | 5 |
| PC 40:5(b) | PC 13:0 13:0 | 836.6 -> 184.1 | 7,845 | 1 | 21 | 5 |
| PC 40:6 | PC 13:0 13:0 | 834.6 -> 184.1 | 7,209 | 1 | 21 | 5 |
| PC 40:7(a) | PC 13:0 13:0 | 832.6 -> 184.1 | 6,137 | 1 | 21 | 5 |
| PC 40:7(b) | PC 13:0 13:0 | 832.6 -> 184.1 | 6,376 | 1 | 21 | 5 |
| PC 40:7(c) | PC 13:0 13:0 | 832.6 -> 184.1 | 6,527 | 1 | 21 | 5 |
| PC 40:8 | PC 13:0 13:0 | 830.6 -> 184.1 | 5,756 | 1 | 21 | 5 |
| PC(O-16:0/0:0) | LPC 13:0 | 482.4 -> 104.1 | 2,983 | 1 | 21 | 5 |
| PC(O-18:0/0:0) | LPC 13:0 | 510.4 -> 104.1 | 3,644 | 1 | 21 | 5 |
| PC(O-18:1/0:0) | LPC 13:0 | 508.4 -> 104.1 | 3,146 | 1 | 21 | 5 |
| PC(O-20:0/0:0) | LPC 13:0 | 538.4 -> 104.1 | 4,171 | 1 | 21 | 5 |
| PC(O-20:1/0:0) | LPC 13:0 | 536.4 -> 104.1 | 3,738 | 1 | 21 | 5 |
| PC(O-22:0/0:0) | LPC 13:0 | 566.5 -> 104.1 | 4,793 | 2 | 21 | 5 |
| PC(O-22:1/0:0) | LPC 13:0 | 564.4 -> 104.1 | 4,242 | 2 | 21 | 5 |
| PC(O-24:0/0:0) | LPC 13:0 | 594.5 -> 104.1 | 5,575 | 2 | 21 | 5 |
| PC(O-24:1/0:0) | LPC 13:0 | 592.5 -> 104.1 | 4,898 | 2 | 21 | 5 |
| PC(O-24:2/0:0) | LPC 13:0 | 590.5 -> 104.1 | 4,356 | 2 | 21 | 5 |
| PC(O-32:0) | PC 13:0 13:0 | 720.6 -> 184.1 | 7,62 | 2 | 21 | 5 |
| PC(O-32:1) | PC 13:0 13:0 | 718.5 -> 184.1 | 6,7 | 2 | 21 | 5 |
| PC(O-32:2) | PC 13:0 13:0 | 716.6 -> 184.1 | 6,031 | 2 | 21 | 5 |
| PC(O-34:1) | PC 13:0 13:0 | 746.6 -> 184.1 | 7,805 | 2 | 21 | 5 |
| PC(O-34:2) | PC 13:0 13:0 | 744.6 -> 184.1 | 7,1 | 2 | 21 | 5 |
| PC(O-34:4) | PC 13:0 13:0 | 740.6 -> 184.1 | 5,984 | 2 | 21 | 5 |
| PC(O-35:4) | PC 13:0 13:0 | 754.5 -> 184.1 | 6,52 | 2 | 21 | 5 |
| PC(O-36:0) | PC 13:0 13:0 | 776.6 -> 184.1 | 9,946 | 2 | 21 | 5 |
| PC(O-36:1) | PC 13:0 13:0 | 774.6 -> 184.1 | 9,008 | 2 | 21 | 5 |
| PC(O-36:2)(a) | PC 13:0 13:0 | 772.6 -> 184.1 | 8,054 | 2 | 21 | 5 |
| PC(O-36:2)(b) | PC 13:0 13:0 | 772.6 -> 184.1 | 8,054 | 2 | 21 | 5 |
| PC(O-36:3)(a) | PC 13:0 13:0 | 770.6 -> 184.1 | 7,27 | 2 | 21 | 5 |
| PC(O-36:3)(b) | PC 13:0 13:0 | 770.6 -> 184.1 | 7,27 | 2 | 21 | 5 |
| PC(O-36:4) | PC 13:0 13:0 | 768.6 -> 184.1 | 6,993 | 2 | 21 | 5 |
| PC(O-36:5) | PC 13:0 13:0 | 766.5 -> 184.1 | 6,368 | 2 | 21 | 5 |
| PC(O-38:4) | PC 13:0 13:0 | 796.6 -> 184.1 | 8,154 | 2 | 21 | 5 |
| PC(O-38:5) | PC 13:0 13:0 | 794.6 -> 184.1 | 7,141 | 2 | 21 | 5 |
| PC(O-38:6) | PC 13:0 13:0 | 792.6 -> 184.1 | 6,837 | 2 | 21 | 5 |
| PC(O-40:5) | PC 13:0 13:0 | 822.6 -> 184.1 | 8,005 | 2 | 21 | 5 |
| PC(O-40:6) | PC 13:0 13:0 | 820.6 -> 184.1 | 7,926 | 2 | 21 | 5 |
| PC(O-40:7)(a) | PC 13:0 13:0 | 818.6 -> 184.1 | 6,988 | 2 | 21 | 5 |
| PC(O-40:7)(b) | PC 13:0 13:0 | 818.6 -> 184.1 | 7,74 | 2 | 21 | 5 |
| PC(P-30:0) | PC 13:0 13:0 | 690.4 -> 184.1 | 6,443 | 2 | 21 | 5 |
| PC(P-32:0) | PC 13:0 13:0 | 718.5 -> 184.1 | 7,448 | 2 | 21 | 5 |
| PC(P-32:1) | PC 13:0 13:0 | 716.6 -> 184.1 | 6,688 | 2 | 21 | 5 |
| PC(P-34:0) | PC 13:0 13:0 | 746.6 -> 184.1 | 8,605 | 2 | 21 | 5 |
| PC(P-34:1) | PC 13:0 13:0 | 744.6 -> 184.1 | 7,699 | 2 | 21 | 5 |
| PC(P-34:2) | PC 13:0 13:0 | 742.5 -> 184.1 | 6,948 | 2 | 21 | 5 |
| PC(P-34:3) | PC 13:0 13:0 | 740.6 -> 184.1 | 6,347 | 2 | 21 | 5 |
| PC(P-35:2)(a) | PC 13:0 13:0 | 756.6 -> 184.1 | 7,26 | 2 | 21 | 5 |
| PC(P-35:2)(b) | PC 13:0 13:0 | 756.6 -> 184.1 | 7,558 | 2 | 21 | 5 |
| PC(P-35:4)(a) | PC 13:0 13:0 | 752.6 -> 184.1 | 6,221 | 2 | 21 | 5 |
| PC(P-35:4)(b) | PC 13:0 13:0 | 752.6 -> 184.1 | 6,369 | 2 | 21 | 5 |
| PC(P-36:2)(a) | PC 13:0 13:0 | 770.6 -> 184.1 | 7,767 | 2 | 21 | 5 |
| PC(P-36:2)(b) | PC 13:0 13:0 | 770.6 -> 184.1 | 8,066 | 2 | 21 | 5 |
| PC(P-36:3) | PC 13:0 13:0 | 768.5 -> 184.1 | 7,259 | 2 | 21 | 5 |
| PC(P-36:4) | PC 13:0 13:0 | 766.5 -> 184.1 | 6,828 | 2 | 21 | 5 |
| PC(P-36:5) | PC 13:0 13:0 | 764.6 -> 184.1 | 6,265 | 2 | 21 | 5 |
| PC(P-37:4)(a) | PC 13:0 13:0 | 780.5 -> 184.1 | 7,234 | 2 | 21 | 5 |
| PC(P-37:4)(b) | PC 13:0 13:0 | 780.5 -> 184.1 | 7,395 | 2 | 21 | 5 |
| PC(P-38:4) | PC 13:0 13:0 | 794.6 -> 184.1 | 7,927 | 2 | 21 | 5 |
| PC(P-38:5)(a) | PC 13:0 13:0 | 792.6 -> 184.1 | 7,025 | 2 | 21 | 5 |
| PC(P-38:5)(b) | PC 13:0 13:0 | 792.6 -> 184.1 | 7,349 | 2 | 21 | 5 |
| PC(P-38:6) | PC 13:0 13:0 | 790.6 -> 184.1 | 6,611 | 2 | 21 | 5 |
| PC(P-40:4) | PC 13:0 13:0 | 822.6 -> 184.1 | 9,081 | 2 | 21 | 5 |
| PC(P-40:5) | PC 13:0 13:0 | 820.6 -> 184.1 | 7,926 | 2 | 21 | 5 |
| PC(P-40:6) | PC 13:0 13:0 | 818.6 -> 184.1 | 7,67 | 2 | 21 | 5 |
| PC(P-40:7) | PC 13:0 13:0 | 816.6 -> 184.1 | 6,894 | 2 | 21 | 5 |
| PE 32:0 | PE 17:0/17:0 | 692.5 -> 551.5 | 7,172 | 2 | 17 | 5 |
| PE 32:1 | PE 17:0/17:0 | 690.5 -> 549.5 | 6,454 | 2 | 17 | 5 |
| PE 34:1 | PE 17:0/17:0 | 718.5 -> 577.5 | 7,402 | 2 | 17 | 5 |
| PE 34:2 | PE 17:0/17:0 | 716.5 -> 575.5 | 6,689 | 2 | 17 | 5 |
| PE 34:3(a) | PE 17:0/17:0 | 714.5 -> 573.5 | 6,043 | 2 | 17 | 5 |
| PE 34:3(b) | PE 17:0/17:0 | 714.5 -> 573.5 | 6,043 | 2 | 17 | 5 |
| PE 34:3(c) | PE 17:0/17:0 | 714.5 -> 573.5 | 6,177 | 2 | 17 | 5 |
| PE 35:1(a) | PE 17:0/17:0 | 732.6 -> 591.5 | 7,677 | 2 | 17 | 5 |
| PE 35:1(b) | PE 17:0/17:0 | 732.6 -> 591.5 | 7,876 | 2 | 17 | 5 |
| PE 35:2(a) | PE 17:0/17:0 | 730.5 -> 589.5 | 7,08 | 2 | 17 | 5 |
| PE 35:2(b) | PE 17:0/17:0 | 730.5 -> 589.5 | 7,28 | 2 | 17 | 5 |
| PE 36:0 | PE 17:0/17:0 | 748.6 -> 607.6 | 9,539 | 2 | 17 | 5 |
| PE 36:1 | PE 17:0/17:0 | 746.6 -> 605.6 | 8,56 | 2 | 17 | 5 |
| PE 36:2(a) | PE 17:0/17:0 | 744.6 -> 603.5 | 7,536 | 2 | 17 | 5 |
| PE 36:2(b) | PE 17:0/17:0 | 744.6 -> 603.5 | 7,875 | 2 | 17 | 5 |
| PE 36:3(a) | PE 17:0/17:0 | 742.5 -> 601.5 | 6,972 | 2 | 17 | 5 |
| PE 36:3(b) | PE 17:0/17:0 | 742.5 -> 601.5 | 6,972 | 2 | 17 | 5 |
| PE 36:4 | PE 17:0/17:0 | 740.5 -> 599.5 | 6,628 | 2 | 17 | 5 |
| PE 36:5(a) | PE 17:0/17:0 | 738.5 -> 597.5 | 5,839 | 2 | 17 | 5 |
| PE 36:5(b) | PE 17:0/17:0 | 738.5 -> 597.5 | 5,984 | 2 | 17 | 5 |
| PE 37:4 (a) | PE 17:0/17:0 | 754.6 -> 613.5 | 6,959 | 2 | 17 | 5 |
| PE 37:4 (b) | PE 17:0/17:0 | 754.6 -> 613.5 | 7,249 | 2 | 17 | 5 |
| PE 38:3(a) | PE 17:0/17:0 | 770.6 -> 629.6 | 8,246 | 2 | 17 | 5 |
| PE 38:3(b) | PE 17:0/17:0 | 770.6 -> 629.6 | 8,592 | 2 | 17 | 5 |
| PE 38:4 | PE 17:0/17:0 | 768.6 -> 627.5 | 7,696 | 2 | 17 | 5 |
| PE 38:5(a) | PE 17:0/17:0 | 766.5 -> 625.5 | 6,768 | 2 | 17 | 5 |
| PE 38:5(b) | PE 17:0/17:0 | 766.5 -> 625.5 | 7,075 | 2 | 17 | 5 |
| PE 38:6 | PE 17:0/17:0 | 764.5 -> 623.5 | 6,438 | 2 | 17 | 5 |
| PE 39:6(a) | PE 17:0/17:0 | 778.5 -> 637.5 | 6,755 | 2 | 17 | 5 |
| PE 39:6(b) | PE 17:0/17:0 | 778.5 -> 637.5 | 7,05 | 2 | 17 | 5 |
| PE 40:4(a) | PE 17:0/17:0 | 796.6 -> 655.6 | 8,655 | 2 | 17 | 5 |
| PE 40:4(b) | PE 17:0/17:0 | 796.6 -> 655.6 | 8,655 | 2 | 17 | 5 |
| PE 40:5(a) | PE 17:0/17:0 | 794.6 -> 653.6 | 7,916 | 2 | 17 | 5 |
| PE 40:5(b) | PE 17:0/17:0 | 794.6 -> 653.6 | 8,321 | 2 | 17 | 5 |
| PE 40:6 | PE 17:0/17:0 | 792.6 -> 651.5 | 7,498 | 2 | 17 | 5 |
| PE 40:7(a) | PE 17:0/17:0 | 790.5 -> 649.5 | 6,565 | 2 | 17 | 5 |
| PE 40:7(b) | PE 17:0/17:0 | 790.5 -> 649.5 | 6,802 | 2 | 17 | 5 |
| PE(O-34:1) | PE 17:0/17:0 | 704.6 -> 563.5 | 8,171 | 2 | 17 | 5 |
| PE(O-34:2) | PE 17:0/17:0 | 702.5 -> 561.5 | 7,368 | 2 | 17 | 5 |
| PE(O-36:3)(a) | PE 17:0/17:0 | 728.6 -> 587.5 | 7,65 | 2 | 17 | 5 |
| PE(O-36:3)(b) | PE 17:0/17:0 | 728.6 -> 587.5 | 7,689 | 2 | 17 | 5 |
| PE(O-36:4) | PE 17:0/17:0 | 726.5 -> 585.5 | 7,424 | 2 | 17 | 5 |
| PE(O-36:5) | PE 17:0/17:0 | 724.5 -> 583.5 | 6,75 | 2 | 17 | 5 |
| PE(O-38:4)(a) | PE 17:0/17:0 | 754.6 -> 613.6 | 8,258 | 2 | 17 | 5 |
| PE(O-38:4)(b) | PE 17:0/17:0 | 754.6 -> 613.6 | 8,615 | 2 | 17 | 5 |
| PE(O-38:5)(a) | PE 17:0/17:0 | 752.6 -> 611.5 | 7,387 | 2 | 17 | 5 |
| PE(O-38:5)(b) | PE 17:0/17:0 | 752.6 -> 611.5 | 7,885 | 2 | 17 | 5 |
| PE(O-38:6) | PE 17:0/17:0 | 750.6 -> 609.5 | 7,076 | 2 | 17 | 5 |
| PE(O-40:5) | PE 17:0/17:0 | 780.6 -> 639.6 | 8,711 | 2 | 17 | 5 |
| PE(O-40:6) | PE 17:0/17:0 | 778.5 -> 637.5 | 8,367 | 2 | 17 | 5 |
| PE(O-40:7) | PE 17:0/17:0 | 776.6 -> 635.5 | 7,35 | 2 | 17 | 5 |
| PE(P-15:0/20:4)(a) | PE 17:0/17:0 | 710.5 -> 361.3 | 6,54 | 2 | 17 | 5 |
| PE(P-15:0/20:4)(b) | PE 17:0/17:0 | 710.5 -> 361.3 | 6,7 | 2 | 17 | 5 |
| PE(P-15:0/22:6)(a) | PE 17:0/17:0 | 734.5 -> 385.3 | 6,35 | 2 | 17 | 5 |
| PE(P-15:0/22:6)(b) | PE 17:0/17:0 | 734.5 -> 385.3 | 6,55 | 2 | 17 | 5 |
| PE(P-16:0/18:1) | PE 17:0/17:0 | 702.5 -> 339.3 | 7,98 | 2 | 17 | 5 |
| PE(P-16:0/18:2) | PE 17:0/17:0 | 700.5 -> 337.3 | 7,241 | 2 | 17 | 5 |
| PE(P-16:0/18:3) | PE 17:0/17:0 | 698.5 -> 335.3 | 6,678 | 2 | 17 | 5 |
| PE(P-16:0/20:3)(a) | PE 17:0/17:0 | 726.5 -> 363.3 | 7,67 | 2 | 17 | 5 |
| PE(P-16:0/20:3)(b) | PE 17:0/17:0 | 726.5 -> 363.3 | 7,8 | 2 | 17 | 5 |
| PE(P-16:0/20:4) | PE 17:0/17:0 | 724.5 -> 361.3 | 7,148 | 2 | 17 | 5 |
| PE(P-16:0/20:5) | PE 17:0/17:0 | 722.5 -> 359.3 | 6,617 | 2 | 17 | 5 |
| PE(P-16:0/22:4) | PE 17:0/17:0 | 752.6 -> 389.3 | 8,1 | 2 | 17 | 5 |
| PE(P-16:0/22:5)(a) | PE 17:0/17:0 | 750.5 -> 387.3 | 7,41 | 2 | 17 | 5 |
| PE(P-16:0/22:5)(b) | PE 17:0/17:0 | 750.5 -> 387.3 | 7,399 | 2 | 17 | 5 |
| PE(P-16:0/22:6) | PE 17:0/17:0 | 748.5 -> 385.3 | 6,924 | 2 | 17 | 5 |
| PE(P-17:0/20:4)(a) | PE 17:0/17:0 | 738.6 -> 361.3 | 7,653 | 2 | 17 | 5 |
| PE(P-17:0/20:4)(b) | PE 17:0/17:0 | 738.6 -> 361.3 | 7,653 | 2 | 17 | 5 |
| PE(P-17:0/22:6)(a) | PE 17:0/17:0 | 762.6 -> 385.3 | 7,443 | 2 | 17 | 5 |
| PE(P-17:0/22:6)(b) | PE 17:0/17:0 | 762.6 -> 385.3 | 7,443 | 2 | 17 | 5 |
| PE(P-18:0/18:1) | PE 17:0/17:0 | 730.6 -> 339.3 | 9,214 | 2 | 17 | 5 |
| PE(P-18:0/18:2) | PE 17:0/17:0 | 728.6 -> 337.3 | 8,315 | 2 | 17 | 5 |
| PE(P-18:0/18:3) | PE 17:0/17:0 | 726.5 -> 335.3 | 7,527 | 2 | 17 | 5 |
| PE(P-18:0/20:3)(a) | PE 17:0/17:0 | 754.5 -> 363.3 | 8,748 | 2 | 17 | 5 |
| PE(P-18:0/20:3)(b) | PE 17:0/17:0 | 754.5 -> 363.3 | 8,911 | 2 | 17 | 5 |
| PE(P-18:0/20:4) | PE 17:0/17:0 | 752.6 -> 361.3 | 8,224 | 2 | 17 | 5 |
| PE(P-18:0/20:5) | PE 17:0/17:0 | 750.5 -> 359.3 | 7,535 | 2 | 17 | 5 |
| PE(P-18:0/22:4) | PE 17:0/17:0 | 780.6 -> 389.3 | 9,147 | 2 | 17 | 5 |
| PE(P-18:0/22:5)(a) | PE 17:0/17:0 | 778.5 -> 387.3 | 8,2 | 2 | 17 | 5 |
| PE(P-18:0/22:5)(b) | PE 17:0/17:0 | 778.5 -> 387.3 | 8,524 | 2 | 17 | 5 |
| PE(P-18:0/22:6) | PE 17:0/17:0 | 776.6 -> 385.3 | 7,974 | 2 | 17 | 5 |
| PE(P-18:1/18:1)(a) | PE 17:0/17:0 | 728.6 -> 339.3 | 8,182 | 2 | 17 | 5 |
| PE(P-18:1/18:1)(b) | PE 17:0/17:0 | 728.6 -> 339.3 | 8,438 | 2 | 17 | 5 |
| PE(P-18:1/18:2)(a) | PE 17:0/17:0 | 726.5 -> 337.3 | 7,55 | 2 | 17 | 5 |
| PE(P-18:1/18:2)(b) | PE 17:0/17:0 | 726.5 -> 337.3 | 7,725 | 2 | 17 | 5 |
| PE(P-18:1/18:3) | PE 17:0/17:0 | 724.5 -> 335.3 | 6,748 | 2 | 17 | 5 |
| PE(P-18:1/20:3)(a) | PE 17:0/17:0 | 752.5 -> 363.3 | 7,908 | 2 | 17 | 5 |
| PE(P-18:1/20:3)(b) | PE 17:0/17:0 | 752.5 -> 363.3 | 8,022 | 2 | 17 | 5 |
| PE(P-18:1/20:4)(a) | PE 17:0/17:0 | 750.5 -> 361.3 | 7,192 | 2 | 17 | 5 |
| PE(P-18:1/20:4)(b) | PE 17:0/17:0 | 750.5 -> 361.3 | 7,581 | 2 | 17 | 5 |
| PE(P-18:1/20:5)(a) | PE 17:0/17:0 | 748.5 -> 359.3 | 6,794 | 2 | 17 | 5 |
| PE(P-18:1/20:5)(b) | PE 17:0/17:0 | 748.5 -> 359.3 | 6,9 | 2 | 17 | 5 |
| PE(P-18:1/22:4) | PE 17:0/17:0 | 778.5 -> 389.3 | 8,233 | 2 | 17 | 5 |
| PE(P-18:1/22:5)(a) | PE 17:0/17:0 | 776.6 -> 387.3 | 7,556 | 2 | 17 | 5 |
| PE(P-18:1/22:5)(b) | PE 17:0/17:0 | 776.6 -> 387.3 | 7,718 | 2 | 17 | 5 |
| PE(P-18:1/22:6)(a) | PE 17:0/17:0 | 774.5 -> 385.3 | 7,235 | 2 | 17 | 5 |
| PE(P-18:1/22:6)(b) | PE 17:0/17:0 | 774.5 -> 385.3 | 7,293 | 2 | 17 | 5 |
| PE(P-19:0/20:4)(a) | PE 17:0/17:0 | 766.6 -> 361.3 | 8,834 | 2 | 17 | 5 |
| PE(P-19:0/20:4)(b) | PE 17:0/17:0 | 766.6 -> 361.3 | 9,03 | 2 | 17 | 5 |
| PE(P-20:0/18:1) | PE 17:0/17:0 | 758.6 -> 339.3 | 10,026 | 2 | 17 | 5 |
| PE(P-20:0/18:2) | PE 17:0/17:0 | 756.6 -> 337.3 | 9,636 | 2 | 17 | 5 |
| PE(P-20:0/20:4) | PE 17:0/17:0 | 780.6 -> 361.3 | 9,451 | 2 | 17 | 5 |
| PE(P-20:0/22:6) | PE 17:0/17:0 | 804.6 -> 385.3 | 9,287 | 2 | 17 | 5 |
| PE(P-20:1/20:4) | PE 17:0/17:0 | 778.5 -> 361.3 | 8,311 | 2 | 17 | 5 |
| PE(P-20:1/22:6)(a) | PE 17:0/17:0 | 802.6 -> 385.3 | 8,187 | 2 | 17 | 5 |
| PE(P-20:1/22:6)(b) | PE 17:0/17:0 | 802.6 -> 385.3 | 8,309 | 2 | 17 | 5 |
| PG 16:0 18:1 | PG 17:0 17:0 | 766.6 -> 577.5 | 6,106 | 2 | 21 | 5 |
| PG 18:0 18:1 | PG 17:0 17:0 | 794.6 -> 605.6 | 7,013 | 2 | 21 | 5 |
| PG 18:1 18:1 | PG 17:0 17:0 | 792.6 -> 603.5 | 6,389 | 2 | 21 | 5 |
| PG 34:2 | PG 17:0 17:0 | 764.6 -> 575.5 | 5,604 | 2 | 21 | 5 |
| PI 32:0 | PE 17:0/17:0 | 828.6 -> 551.6 | 5,712 | 2 | 17 | 5 |
| PI 32:1 | PE 17:0/17:0 | 826.5 -> 549.5 | 5,172 | 2 | 17 | 5 |
| PI 34:0 | PE 17:0/17:0 | 856.6 -> 579.6 | 6,503 | 2 | 17 | 5 |
| PI 34:1 | PE 17:0/17:0 | 854.6 -> 577.6 | 5,845 | 2 | 17 | 5 |
| PI 35:1 | PE 17:0/17:0 | 868.6 -> 591.6 | 6,25 | 2 | 17 | 5 |
| PI 35:2 | PE 17:0/17:0 | 866.6 -> 589.6 | 5,7 | 2 | 17 | 5 |
| PI 36:1 | PE 17:0/17:0 | 882.6 -> 605.6 | 6,787 | 2 | 17 | 5 |
| PI 36:2 | PE 17:0/17:0 | 880.6 -> 603.6 | 6,079 | 2 | 17 | 5 |
| PI 36:3(a) | PE 17:0/17:0 | 878.6 -> 601.6 | 5,4 | 2 | 17 | 5 |
| PI 36:3(b) | PE 17:0/17:0 | 878.6 -> 601.6 | 5,52 | 2 | 17 | 5 |
| PI 36:3(c) | PE 17:0/17:0 | 878.6 -> 601.6 | 5,844 | 2 | 17 | 5 |
| PI 36:4 | PE 17:0/17:0 | 876.6 -> 599.6 | 5,342 | 2 | 17 | 5 |
| PI 37:4 | PE 17:0/17:0 | 890.6 -> 613.6 | 5,644 | 2 | 17 | 5 |
| PI 37:6 | PE 17:0/17:0 | 886.6 -> 609.6 | 5,611 | 2 | 17 | 5 |
| PI 38:2 | PE 17:0/17:0 | 908.6 -> 631.6 | 6,964 | 2 | 17 | 5 |
| PI 38:3(a) | PE 17:0/17:0 | 906.6 -> 629.6 | 6,135 | 2 | 17 | 5 |
| PI 38:3(b) | PE 17:0/17:0 | 906.6 -> 629.6 | 6,467 | 2 | 17 | 5 |
| PI 38:4 | PE 17:0/17:0 | 904.6 -> 627.6 | 6,09 | 2 | 17 | 5 |
| PI 38:5(a) | PE 17:0/17:0 | 902.6 -> 625.6 | 5,469 | 2 | 17 | 5 |
| PI 38:5(b) | PE 17:0/17:0 | 902.6 -> 625.6 | 5,577 | 2 | 17 | 5 |
| PI 38:6 | PE 17:0/17:0 | 900.6 -> 623.6 | 5,213 | 2 | 17 | 5 |
| PI 39:6 | PE 17:0/17:0 | 914.6 -> 637.6 | 5,523 | 2 | 17 | 5 |
| PI 40:4(a) | PE 17:0/17:0 | 932.6 -> 655.6 | 6,787 | 2 | 17 | 5 |
| PI 40:4(b) | PE 17:0/17:0 | 932.6 -> 655.6 | 6,787 | 2 | 17 | 5 |
| PI 40:5(a) | PE 17:0/17:0 | 930.6 -> 653.6 | 6,226 | 2 | 17 | 5 |
| PI 40:5(b) | PE 17:0/17:0 | 930.6 -> 653.6 | 6,526 | 2 | 17 | 5 |
| PI 40:6 | PE 17:0/17:0 | 928.6 -> 651.6 | 5,955 | 2 | 17 | 5 |
| PS 36:1 | PS 17:0 17:0 | 790.6 -> 605.6 | 6,978 | 2 | 25 | 5 |
| PS 36:2 | PS 17:0 17:0 | 788.5 -> 603.5 | 6,378 | 2 | 25 | 5 |
| PS 38:3 | PS 17:0 17:0 | 814.6 -> 629.6 | 6,575 | 2 | 25 | 5 |
| PS 38:4 | PS 17:0 17:0 | 812.5 -> 627.5 | 6,366 | 2 | 25 | 5 |
| PS 38:5 | PS 17:0 17:0 | 810.5 -> 625.5 | 5,658 | 2 | 25 | 5 |
| PS 40:5 | PS 17:0 17:0 | 838.6 -> 653.6 | 6,319 | 2 | 25 | 5 |
| PS 40:6 | PS 17:0 17:0 | 836.5 -> 651.5 | 6,182 | 2 | 25 | 5 |
| S1P(d16:1) | Sph(d17:1) | 352.2 -> 236.3 | 1,566 | 3 | 16 | 4 |
| S1P(d17:1) | Sph(d17:1) | 366.2 -> 250.3 | 1,969 | 3 | 16 | 4 |
| S1P(d18:0) | Sph(d17:1) | 382.2 -> 284.3 | 2,735 | 3 | 11 | 4 |
| S1P(d18:1) | Sph(d17:1) | 380.2 -> 264.3 | 2,293 | 3 | 16 | 4 |
| S1P(d18:2) | Sph(d17:1) | 378.2 -> 262.3 | 1,765 | 3 | 16 | 4 |
| SM 31:1 | SM 30:1 | 661.5 -> 184.1 | 4,789 | 2 | 25 | 5 |
| SM 32:0 | SM 30:1 | 677.6 -> 184.1 | 5,402 | 2 | 25 | 5 |
| SM 32:1 | SM 30:1 | 675.5 -> 184.1 | 5,133 | 2 | 25 | 5 |
| SM 32:2 | SM 30:1 | 673.5 -> 184.1 | 4,661 | 2 | 25 | 5 |
| SM 33:1 | SM 30:1 | 689.6 -> 184.1 | 5,465 | 2 | 25 | 5 |
| SM 34:0 | SM 30:1 | 705.6 -> 184.1 | 6,223 | 2 | 25 | 5 |
| SM 34:1 | SM 30:1 | 703.6 -> 184.1 | 5,874 | 2 | 25 | 5 |
| SM 34:2 | SM 30:1 | 701.6 -> 184.1 | 5,293 | 2 | 25 | 5 |
| SM 34:3 | SM 30:1 | 699.5 -> 184.1 | 4,885 | 2 | 25 | 5 |
| SM 35:1(a) | SM 30:1 | 717.6 -> 184.1 | 6,121 | 2 | 25 | 5 |
| SM 35:1(b) | SM 30:1 | 717.6 -> 184.1 | 6,199 | 2 | 25 | 5 |
| SM 35:2(a) | SM 30:1 | 715.6 -> 184.1 | 5,684 | 2 | 25 | 5 |
| SM 35:2(b) | SM 30:1 | 715.6 -> 184.1 | 5,907 | 2 | 25 | 5 |
| SM 36:1 | SM 30:1 | 731.6 -> 184.1 | 6,82 | 2 | 25 | 5 |
| SM 36:2 | SM 30:1 | 729.6 -> 184.1 | 6,086 | 2 | 25 | 5 |
| SM 36:3 | SM 30:1 | 727.6 -> 184.1 | 5,496 | 2 | 25 | 5 |
| SM 37:1 | SM 30:1 | 745.6 -> 184.1 | 7,088 | 3 | 25 | 5 |
| SM 37:2 | SM 30:1 | 743.5 -> 184.1 | 6,533 | 2 | 25 | 5 |
| SM 38:1 | SM 30:1 | 759.6 -> 184.1 | 7,953 | 2 | 25 | 5 |
| SM 38:2 | SM 30:1 | 757.6 -> 184.1 | 7,04 | 2 | 25 | 5 |
| SM 38:3(a) | SM 30:1 | 755.6 -> 184.1 | 6,255 | 3 | 25 | 5 |
| SM 38:3(b) | SM 30:1 | 755.6 -> 184.1 | 6,358 | 3 | 25 | 5 |
| SM 39:1 | SM 30:1 | 773.7 -> 184.1 | 8,525 | 2 | 25 | 5 |
| SM 40:0 | SM 30:1 | 789.7 -> 184.1 | 9,548 | 2 | 25 | 5 |
| SM 40:1 | SM 30:1 | 787.7 -> 184.1 | 9,114 | 2 | 25 | 5 |
| SM 40:2(a) | SM 30:1 | 785.7 -> 184.1 | 8,076 | 2 | 25 | 5 |
| SM 40:2(b) | SM 30:1 | 785.7 -> 184.1 | 8,277 | 2 | 25 | 5 |
| SM 40:3(a) | SM 30:1 | 783.6 -> 184.1 | 7,223 | 3 | 25 | 5 |
| SM 40:3(b) | SM 30:1 | 783.6 -> 184.1 | 7,223 | 3 | 25 | 5 |
| SM 41:0 | SM 30:1 | 803.7 -> 184.1 | 9,968 | 3 | 25 | 5 |
| SM 41:1(a) | SM 30:1 | 801.7 -> 184.1 | 9,911 | 2 | 25 | 5 |
| SM 41:1(b) | SM 30:1 | 801.7 -> 184.1 | 9,8 | 2 | 25 | 5 |
| SM 41:2(a) | SM 30:1 | 799.7 -> 184.1 | 8,644 | 2 | 25 | 5 |
| SM 41:2(b) | SM 30:1 | 799.7 -> 184.1 | 8,886 | 2 | 25 | 5 |
| SM 42:1 | SM 30:1 | 815.7 -> 184.1 | 10,024 | 2 | 25 | 5 |
| SM 42:2(a) | SM 30:1 | 813.7 -> 184.1 | 9,221 | 2 | 25 | 5 |
| SM 42:2(b) | SM 30:1 | 813.7 -> 184.1 | 9,221 | 2 | 25 | 5 |
| SM 43:1 | SM 30:1 | 829.7 -> 184.1 | 10,15 | 3 | 25 | 5 |
| SM 43:2(a) | SM 30:1 | 827.7 -> 184.1 | 9,633 | 3 | 25 | 5 |
| SM 43:2(b) | SM 30:1 | 827.7 -> 184.1 | 9,633 | 3 | 25 | 5 |
| SM 43:2(c) | SM 30:1 | 827.7 -> 184.1 | 10,024 | 3 | 25 | 5 |
| SM 44:1 | SM 30:1 | 843.6 -> 184.1 | 10,265 | 3 | 25 | 5 |
| SM 44:2 | SM 30:1 | 841.6 -> 184.1 | 10,092 | 3 | 25 | 5 |
| SM 44:3 | SM 30:1 | 839.6 -> 184.1 | 9,275 | 3 | 25 | 5 |
| Sph(d16:1) | Sph(d17:1) | 272.3 -> 254.3 | 3,734 | 3 | 8 | 4 |
| Sph(d18:1) | Sph(d17:1) | 300.3 -> 282.3 | 2,402 | 3 | 8 | 4 |
| Sph(d18:2) | Sph(d17:1) | 298.3 -> 280.3 | 1,693 | 3 | 8 | 4 |
| Sulfatide (d18:1:/16:0(OH)) | Hex1Cer(d18:1/16:0)d3 | 796.8 -> 264.3 | 4,926 | 2 | 56 | 5 |
| Sulfatide (d18:1:/16:0) | Hex1Cer(d18:1/16:0)d3 | 780.8 -> 264.3 | 5,055 | 2 | 56 | 5 |
| Sulfatide (d18:1:/24:0(OH)) | Hex1Cer(d18:1/16:0)d3 | 908.8 -> 264.3 | 8,364 | 2 | 56 | 5 |
| Sulfatide (d18:1:/24:0) | Hex1Cer(d18:1/16:0)d3 | 892.8 -> 264.3 | 8,731 | 2 | 56 | 5 |
| Sulfatide (d18:1:/24:1(OH)) | Hex1Cer(d18:1/16:0)d3 | 906.8 -> 264.3 | 7,335 | 2 | 56 | 5 |
| Sulfatide (d18:1:/24:1) | Hex1Cer(d18:1/16:0)d3 | 890.8 -> 264.3 | 7,669 | 2 | 56 | 5 |
| TG 14:0 16:0 18:2 | TG 17:0 17:0 17:0 | 820.8 -> 547.5 | 11,021 | 2 | 21 | 5 |
| TG 14:0 16:1 18:1 | TG 17:0 17:0 17:0 | 820.8 -> 521.5 | 11,021 | 2 | 21 | 5 |
| TG 14:0 16:1 18:2 | TG 17:0 17:0 17:0 | 818.8 -> 521.5 | 10,875 | 2 | 21 | 5 |
| TG 14:0 18:0 18:1 | TG 17:0 17:0 17:0 | 850.8 -> 605.6 | 11,451 | 2 | 21 | 5 |
| TG 14:0 18:2 18:2 | TG 17:0 17:0 17:0 | 844.8 -> 599.5 | 10,926 | 2 | 21 | 5 |
| TG 14:1 16:0 18:1 | TG 17:0 17:0 17:0 | 820.8 -> 577.6 | 11,031 | 2 | 21 | 5 |
| TG 14:1 16:1 18:0 | TG 17:0 17:0 17:0 | 820.8 -> 549.5 | 11,042 | 2 | 21 | 5 |
| TG 14:1 18:0 18:2 | TG 17:0 17:0 17:0 | 846.8 -> 603.6 | 11,061 | 2 | 21 | 5 |
| TG 14:1 18:1 18:1 | TG 17:0 17:0 17:0 | 846.8 -> 547.5 | 11,061 | 2 | 21 | 5 |
| TG 15:0 18:1 16:0 | TG 17:0 17:0 17:0 | 836.8 -> 577.5 | 11,283 | 2 | 21 | 5 |
| TG 15:0 18:1 18:1 | TG 17:0 17:0 17:0 | 862.8 -> 603.6 | 11,28 | 2 | 21 | 5 |
| TG 16:0 16:0 16:0 | TG 17:0 17:0 17:0 | 824.8 -> 551.5 | 11,378 | 2 | 21 | 5 |
| TG 16:0 16:0 18:0 | TG 17:0 17:0 17:0 | 852.8 -> 551.5 | 11,587 | 2 | 21 | 5 |
| TG 16:0 16:0 18:1 | TG 17:0 17:0 17:0 | 850.8 -> 551.5 | 11,387 | 2 | 21 | 5 |
| TG 16:0 16:0 18:2 | TG 17:0 17:0 17:0 | 848.8 -> 551.5 | 11,229 | 2 | 21 | 5 |
| TG 16:0 16:1 18:1 | TG 17:0 17:0 17:0 | 848.8 -> 549.5 | 11,208 | 2 | 21 | 5 |
| TG 16:0 18:0 18:1 | TG 17:0 17:0 17:0 | 878.8 -> 577.5 | 11,606 | 2 | 21 | 5 |
| TG 16:0 18:1 18:1 | TG 17:0 17:0 17:0 | 876.8 -> 603.6 | 11,417 | 2 | 21 | 5 |
| TG 16:0 18:1 18:2 | TG 17:0 17:0 17:0 | 874.8 -> 577.6 | 11,237 | 2 | 21 | 5 |
| TG 16:0 18:2 18:2 | TG 17:0 17:0 17:0 | 872.8 -> 599.6 | 11,113 | 2 | 21 | 5 |
| TG 16:1 16:1 16:1 | TG 17:0 17:0 17:0 | 818.8 -> 547.5 | 10,918 | 2 | 21 | 5 |
| TG 16:1 16:1 18:0 | TG 17:0 17:0 17:0 | 848.8 -> 547.5 | 11,281 | 2 | 21 | 5 |
| TG 16:1 16:1 18:1 | TG 17:0 17:0 17:0 | 846.8 -> 575.6 | 11,061 | 2 | 21 | 5 |
| TG 16:1 18:1 18:1 | TG 17:0 17:0 17:0 | 874.8 -> 603.6 | 11,248 | 3 | 21 | 5 |
| TG 16:1 18:1 18:2 | TG 17:0 17:0 17:0 | 872.8 -> 573.6 | 11,092 | 3 | 21 | 5 |
| TG 17:0 16:0 16:1 | TG 17:0 17:0 17:0 | 836.8 -> 563.5 | 11,294 | 3 | 21 | 5 |
| TG 17:0 16:0 18:0 | TG 17:0 17:0 17:0 | 866.8 -> 593.6 | 11,753 | 3 | 21 | 5 |
| TG 17:0 18:1 14:0 | TG 17:0 17:0 17:0 | 836.8 -> 537.5 | 11,294 | 3 | 21 | 5 |
| TG 17:0 18:1 16:0 | TG 17:0 17:0 17:0 | 864.8 -> 565.5 | 11,576 | 3 | 21 | 5 |
| TG 17:0 18:1 16:1 | TG 17:0 17:0 17:0 | 862.8 -> 563.5 | 11,323 | 3 | 21 | 5 |
| TG 17:0 18:1 18:1 | TG 17:0 17:0 17:0 | 890.8 -> 603.6 | 11,523 | 3 | 21 | 5 |
| TG 17:0 18:2 16:0 | TG 17:0 17:0 17:0 | 862.8 -> 589.6 | 11,27 | 3 | 21 | 5 |
| TG 18:0 18:0 18:0 | TG 17:0 17:0 17:0 | 908.9 -> 607.6 | 12,039 | 3 | 21 | 5 |
| TG 18:0 18:0 18:1 | TG 17:0 17:0 17:0 | 906.9 -> 607.6 | 11,917 | 3 | 21 | 5 |
| TG 18:0 18:1 18:1 | TG 17:0 17:0 17:0 | 904.9 -> 603.6 | 11,721 | 3 | 21 | 5 |
| TG 18:0 18:2 18:2 | TG 17:0 17:0 17:0 | 900.8 -> 599.5 | 11,363 | 3 | 21 | 5 |
| TG 18:1 14:0 16:0 | TG 17:0 17:0 17:0 | 822.8 -> 523.5 | 11,169 | 3 | 21 | 5 |
| TG 18:1 18:1 18:1 | TG 17:0 17:0 17:0 | 902.9 -> 603.6 | 11,459 | 3 | 21 | 5 |
| TG 18:1 18:1 18:2 | TG 17:0 17:0 17:0 | 900.9 -> 603.9 | 11,342 | 3 | 21 | 5 |
| TG 18:1 18:1 20:4 | TG 17:0 17:0 17:0 | 924.9 -> 603.6 | 11,278 | 3 | 21 | 5 |
| TG 18:1 18:1 22:6 | TG 17:0 17:0 17:0 | 948.9 -> 603.7 | 11,215 | 3 | 21 | 5 |
| TG 18:1 18:2 18:2 | TG 17:0 17:0 17:0 | 898.9 -> 599.6 | 11,133 | 3 | 21 | 5 |
| TG 18:2 18:2 18:2 | TG 17:0 17:0 17:0 | 896.9 -> 599.6 | 11,007 | 3 | 21 | 5 |
| TG 18:2 18:2 20:4 | TG 17:0 17:0 17:0 | 920.9 -> 599.6 | 10,944 | 3 | 21 | 5 |
| TG(O-50:1) | TG 17:0 17:0 17:0 | 836.8 -> 563.5 | 11,692 | 3 | 21 | 5 |
| TG(O-52:0) | TG 17:0 17:0 17:0 | 866.8 -> 593.6 | 11,999 | 3 | 21 | 5 |
| TG(O-52:2) | TG 17:0 17:0 17:0 | 862.8 -> 589.6 | 11,65 | 3 | 21 | 5 |
| Ubiquinone | Hex3Cer(d18:1/17:0) | 880.7 -> 197.0 | 10,882 | 3 | 17 | 5 |
