# Supplementary material for: Lipidomics Reveals a Tissue-Specific Fingerprint
Source: Front Physiol. 2018 Aug 28;9:1165. doi: 10.3389/fphys.2018.01165 (PMC6121266; doi:10.3389/fphys.2018.01165)
Supplement: Supplementary file 3 [file Table_3.docx]

Supplementary Material

**Lipidomics reveals tissue-specific organization of lipids**

Irene Pradas,^1^ Kevin Huynh,^2^ Rosanna Cabré,^1^ Victòria Ayala,^1^ Peter J Meikle,^2^ Mariona Jové,^1^* and Reinald Pamplona^1^*

^1^Department of Experimental Medicine, University of Lleida-Institute for Research in Biomedicine of Lleida (UdL-IRBLleida), E-25198 Lleida, Spain

^2^Baker Heart and Diabetes Institute, Melbourne VIC 3004, Australia

*** Correspondence:**Dr. Mariona Jové, Departament de Medicina Experimental, Universitat de Lleida-Institut de Recerca Biomedica de Lleida (IRBLleida), Edifici Biomedicina 1, Av. Alcalde Rovira Roure-80, Lleida 25198, Catalonia, Spain. Phone: (+34)973702442

[mariona.jove@udl.cat](mailto:mariona.jove@udl.cat)

Prof. Dr. Reinald Pamplona, Departament de Medicina Experimental, Universitat de Lleida-Institut de Recerca Biomedica de Lleida (IRBLleida), Edifici Biomedicina 1, Av. Alcalde Rovira Roure-80, Lleida 25198, Catalonia, Spain. Phone: (+34)973702442

[reinald.pamplona@mex.udl.cat](mailto:reinald.pamplona@mex.udl.cat)

# Supplementary Tables

**Table S3.** Clusters analysis of lipid profiles. Definition of the clusters chosen to be analyzed in the heat map of Pearson correlation matrix of figure 9.

| Cluster | Cluster a | Cluster b | Cluster c | Cluster d | Cluster e | Cluster f |
| --- | --- | --- | --- | --- | --- | --- |
| Nº of lipid species | 34 | 27 | 22 | 32 | 40 | 29 |
| Nº of lipid classes | 3 | 9 | 7 | 8 | 9 | 10 |
| Most abundant lipid class | TAG | PE(P-) | SM | Cer | SM | SM |
| List of Lipids in each cluster | DG(16:0/16:1) | PC(P-38:5) (a) | LPC(20:2) [sn1] | LPE(18:2) [sn2] | LPC(18:0) [sn2] | LPC(24:0) [sn2] |
|  | DG(16:1/18:1) | PC(P-38:5) (b) | PE(16:1_18:2) | LPE(18:2) [sn1] | LPC(19:0) [sn2] (a) | LPC(24:0) [sn1] |
|  | DG(18:1/18:2) | PE(18:0_22:5) (n6) | PE(16:0_18:3) (a) | LPE(20:4) [sn2] | LPC(20:0) [sn2] | LPC(26:0) [sn2] |
|  | TG(14:0/16:0/18:2) | PE(18:0_22:6) | PE(16:0_20:4) | LPE(20:4) [sn1] | LPC(20:0) [sn1] | LPC(26:0) [sn1] |
|  | TG(14:0/16:1/18:1) | PE(O-16:0/22:6) | PE(16:0_20:5) | LPI(18:2) [sn2] | LPC(22:0) [sn2] | LPC(O-16:0) |
|  | TG(14:0/16:1/18:2) | PE(O-18:0/22:5) (a) | PE(15-MHDA_20:4) | LPI(18:2) [sn1] | LPC(22:0) [sn1] | PC(31:0) (b) |
|  | TG(14:0/18:0/18:1) | PE(O-18:0/22:6) | PI(16:0/20:3) (a) | LPI(20:4) [sn2] | LPC(22:1) [sn2] | PC(16:0_16:0) |
|  | TG(14:0/18:2/18:2) | PE(O-18:1/22:6) | PI(16:0_20:4) | LPI(20:4) [sn1] | LPC(22:1) [sn1] | PE(16:0_16:0) |
|  | TG(14:1/16:0/18:1) | PE(P-15:0/22:6) (b) | PI(20:0_20:4) | PC(16:1_18:2) | LPC(O-18:0) | PE(O-16:0/18:2) |
|  | TG(14:1/16:1/18:0) | PE(P-16:0/22:5) (n3) | Cer(d17:1/16:0) | PC(16:0_18:3) (b) | LPC(O-18:1) | PE(O-18:1/18:2) |
|  | TG(14:1/18:0/18:2) | PE(P-16:0/22:5) (n6) | Cer(d17:1/24:0) | PC(34:5) | LPC(O-20:0) | PE(O-16:0/20:4) |
|  | TG(14:1/18:1/18:1) | PE(P-16:0/22:6) | Cer(d18:1/22:0) (a) | PC(35:5) | LPC(O-22:0) | PE(O-18:0/20:4) |
|  | TG(15:0/16:0/18:1) | PE(P-17:0/22:6) (b) | Cer(d18:1/24:0) (a) | PE(16:0_18:2) | LPE(18:0) [sn1] | PE(P-15:0/20:4) (b) |
|  | TG(15:0/18:1/18:1) | PE(P-18:0/22:5) (n6) | Cer(d18:1/24:1) (a) | PE(16:0_18:3) (b) | LPE(18:1) [sn1] | PE(P-16:0/18:3) |
|  | TG(16:0/16:0/16:0) | PE(P-18:1/22:5) (a) | Cer(d19:1/24:0) | PE(15-MHDA_18:2) | PC(O-16:0/16:0) | PI(16:0/16:0) |
|  | TG(16:0/16:0/18:1) | PE(P-18:1/22:5) (b) | Cer(d18:0/24:1) | PI(17:0_18:2) (a+b) | PC(O-35:4) | PI(34:0) |
|  | TG(16:0/16:0/18:2) | PE(P-18:1/22:6) (a) | Cer(d18:0/16:0) | PI(16:0/20:3) (b) | PC(O-36:0) | PS(36:1) |
|  | TG(16:0/16:1/18:1) | PE(P-18:1/22:6) (b) | Cer(d18:0/22:0) | PI(17:0_20:4) (a+b) | PC(O-18:0/18:2) | Sph(d18:2) |
|  | TG(16:1/16:1/16:1) | PE(P-20:0/22:6) | Cer(d18:0/24:0) | Cer(d16:1/24:0) | PC(O-18:1/18:2) | SM(d18:1/16:0) |
|  | TG(16:1/16:1/18:0) | PE(P-20:1/22:6) (a) | SM(41:0) | Cer(d16:1/24:1) | PC(O-16:0/20:3) | SM(d18:2/16:0) |
|  | TG(16:1/16:1/18:1) | PG(34:1) | SM(43:1) | Cer(d17:1/22:0) | PC(O-16:0/20:4) | SM(34:3) |
|  | TG(16:1/18:1/18:1) | PG(36:2) | CE(18:3) | Cer(d17:1/23:0) | PC(O-18:0/20:4) | SM(d18:1/17:0)/  SM(d17:1/18:0) |
|  | TG(16:1/18:1/18:2) | PS(40:5) |  | Cer(d18:1/14:0) | PC(O-38:5) | SM(d18:2/17:0) |
|  | TG(16:0/17:0/18:1) | PS(40:6) |  | Cer(d18:1/23:0) | PC(P-20:0/20:4) | SM(35:2) (b) |
|  | TG(16:1/17:0/18:1) | SM(d18:2/20:0) |  | Cer(d19:1/22:0) | PI(16:0_16:1) | SM(d18:2/22:0) |
|  | TG(17:0/18:1/18:1) | Hex2Cer(d18:1/20:0) |  | Cer(d19:1/23:0) | PS(38:3) | SM(d18:2/24:0) |
|  | TG(16:0/17:0/18:2) | GM3(d18:1/20:0) |  | Desmosterol(18:2) | PS(38:4) | SM(43:2) (c) |
|  | TG(18:0/18:2/18:2) |  |  | CE(16:1) | SM(d18:1/14:0)/  SM(d16:1/16:0) | SM(44:1) |
|  | TG(14:0/16:0/18:1) |  |  | CE(17:0) | SM(d18:2/14:0) | SM(44:3) |
|  | TG(18:1/18:1/18:1) |  |  | CE(17:1) | SM(d17:1/16:0) |  |
|  | TG(18:1/18:1/18:2) |  |  | CE(18:1) | SM(d18:0/16:0) |  |
|  | TG(18:1/18:2/18:2) |  |  | CE(18:2) | SM(d16:1/19:0) |  |
|  | TG(18:2/18:2/18:2) |  |  |  | SM(d18:2/18:1) |  |
|  | oxCE (18:2) [+2O] |  |  |  | SM(38:3) (b) |  |
|  |  |  |  |  | SM(d18:1/22:0)/  SM(d16:1/24:0) |  |
|  |  |  |  |  | SM(d18:1/24:0) |  |
|  |  |  |  |  | SM(d18:1/24:1) |  |
|  |  |  |  |  | SM(43:2) (b) |  |
|  |  |  |  |  | SM(44:2) |  |
|  |  |  |  |  | GM3(d18:1/16:0) |  |
