# Supplementary material for: Lipidomics Reveals a Tissue-Specific Fingerprint
Source: Front Physiol. 2018 Aug 28;9:1165. doi: 10.3389/fphys.2018.01165 (PMC6121266; doi:10.3389/fphys.2018.01165)

Supplementary Material

**Lipidomics reveals tissue-specific organization of lipids**

Irene Pradas,^1^ Kevin Huynh,^2^ Rosanna Cabré,^1^ Victòria Ayala,^1^ Peter J Meikle,^2^ Mariona Jové,^1^* and Reinald Pamplona^1^*

^1^Department of Experimental Medicine, University of Lleida-Institute for Research in Biomedicine of Lleida (UdL-IRBLleida), E-25198 Lleida, Spain

^2^Baker Heart and Diabetes Institute, Melbourne VIC 3004, Australia

*** Correspondence:**Dr. Mariona Jové, Departament de Medicina Experimental, Universitat de Lleida-Institut de Recerca Biomedica de Lleida (IRBLleida), Edifici Biomedicina 1, Av. Alcalde Rovira Roure-80, Lleida 25198, Catalonia, Spain. Phone: (+34)973702442

[mariona.jove@udl.cat](mailto:mariona.jove@udl.cat)

Prof. Dr. Reinald Pamplona, Departament de Medicina Experimental, Universitat de Lleida-Institut de Recerca Biomedica de Lleida (IRBLleida), Edifici Biomedicina 1, Av. Alcalde Rovira Roure-80, Lleida 25198, Catalonia, Spain. Phone: (+34)973702442

[reinald.pamplona@mex.udl.cat](mailto:reinald.pamplona@mex.udl.cat)

**Supplementary Figure 1.** Extracted chromatogram of PE(36:2) from a pooled plasma sample run in with polarity switching in positive and negative ion mode. Black trace: Positive ion mode examining the neutral loss of the head group, 141 Da, corresponding to PE(36:2). Coloured trace - Negative ion mode was used to confirm isomer identity of peak at RT 7.74, PE(18:1/18:1) and RT 7.88, PE(18:0_18:2) with product ions corresponding to each fatty acid.


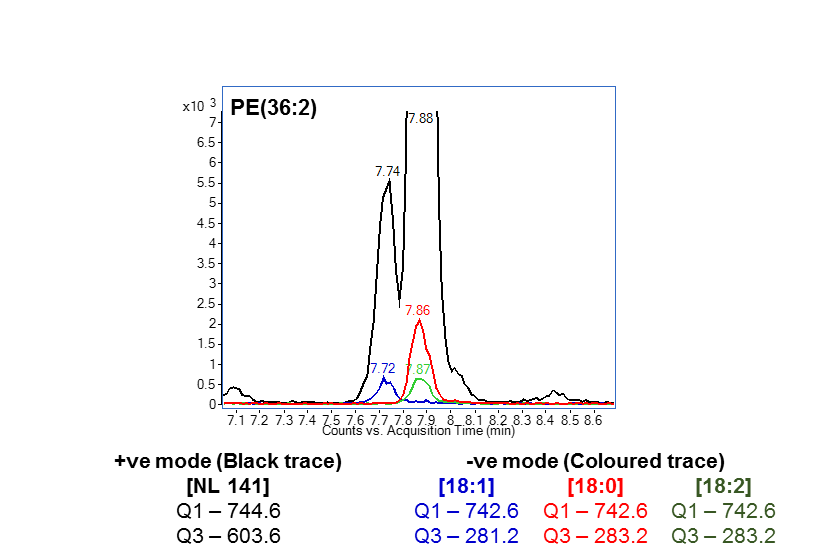

Supplement: Supplementary file 4 [file Table_4.docx]
