# Supplementary material for: Lipidomics Reveals a Tissue-Specific Fingerprint
Source: Front Physiol. 2018 Aug 28;9:1165. doi: 10.3389/fphys.2018.01165 (PMC6121266; doi:10.3389/fphys.2018.01165)
Supplement: Supplementary file 5 [file Table_5.docx]

Supplementary Material

**Lipidomics reveals tissue-specific organization of lipids**

Irene Pradas,^1^ Kevin Huynh,^2^ Rosanna Cabré,^1^ Victòria Ayala,^1^ Peter J Meikle,^2^ Mariona Jové,^1^* and Reinald Pamplona^1^*

^1^Department of Experimental Medicine, University of Lleida-Institute for Research in Biomedicine of Lleida (UdL-IRBLleida), E-25198 Lleida, Spain

^2^Baker Heart and Diabetes Institute, Melbourne VIC 3004, Australia

*** Correspondence:**Dr. Mariona Jové, Departament de Medicina Experimental, Universitat de Lleida-Institut de Recerca Biomedica de Lleida (IRBLleida), Edifici Biomedicina 1, Av. Alcalde Rovira Roure-80, Lleida 25198, Catalonia, Spain. Phone: (+34)973702442

[mariona.jove@udl.cat](mailto:mariona.jove@udl.cat)

Prof. Dr. Reinald Pamplona, Departament de Medicina Experimental, Universitat de Lleida-Institut de Recerca Biomedica de Lleida (IRBLleida), Edifici Biomedicina 1, Av. Alcalde Rovira Roure-80, Lleida 25198, Catalonia, Spain. Phone: (+34)973702442

[reinald.pamplona@mex.udl.cat](mailto:reinald.pamplona@mex.udl.cat)

**Supplementary Figure 2.** Extracted chromatogram of 770.4 / 184.1 corresponding to PC(35:3), PC(O-36:3) and PC(P-36:2). Sequential examination of normal pooled plasma lipid extracts (Black trace) and acid treated lipid extracts (red trace) was used to extensively determine plasmalogen species from other isomer and isobaric compounds. Identify of each species was further characterised in a combination of other fragmentation experiments.


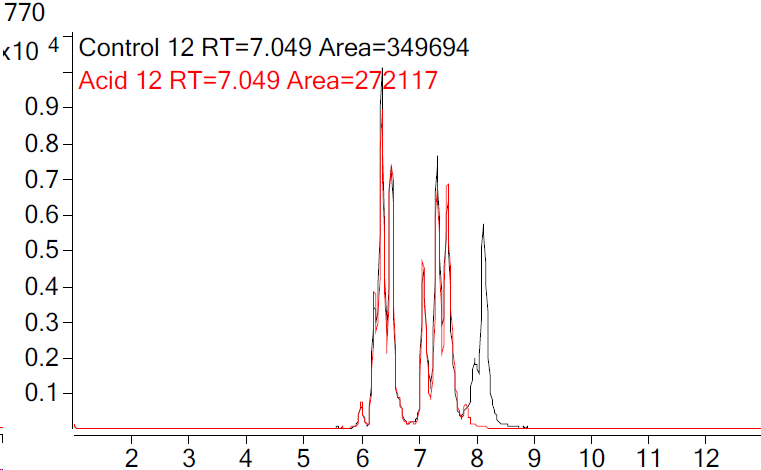


Retention time (minute)

**Q1 – 770.4**

**Q3 – 184.1**

Control QC sample

Acid treated QC sample

PC(35:3)

PC(O-36:3)

PC(P-36:2)

Intensity

a

b

c

d

e

f

g

h

**Subsequent identification**

a) PC(17:1_18:2)

b) PC(15:0_20:3)

c) +1 isotope of SM(39:3) + other

d) +2 isotope of PC(O-36:4)

e) PC(O-18:1/18:2)

f ) PC(O-16:0/20:3)

g) PC(P-18:1/18:1)

h) PC(P-18:0/18:2)
